# Rational design of iron catalysts for C**–**X bond activation

**DOI:** 10.1002/jcc.26818

**Published:** 2022-02-08

**Authors:** Xiaobo Sun, Thomas Hansen, Jordi Poater, Trevor A. Hamlin, Friedrich Matthias Bickelhaupt

**Affiliations:** ^1^ Department of Theoretical Chemistry and Amsterdam Center for Multiscale Modeling (ACMM) Vrije Universiteit Amsterdam Amsterdam The Netherlands; ^2^ Departament de Química Inorgànica i Orgànica & IQTCUB Universitat de Barcelona Barcelona Spain; ^3^ Leiden Institute of Chemistry Leiden University Leiden The Netherlands; ^4^ ICREA Barcelona Spain; ^5^ Institute for Molecules and Materials (IMM) Radboud University Nijmegen The Netherlands

**Keywords:** activation strain model, bond activation, earth‐abundant metal catalysis, iron, rational design

## Abstract

We have quantum chemically studied the iron‐mediated C—X bond activation (X = H, Cl, CH_3_) by d^8^‐FeL_4_ complexes using relativistic density functional theory at ZORA‐OPBE/TZ2P. We find that by either modulating the electronic effects of a generic iron‐catalyst by a set of ligands, that is, CO, BF, PH_3_, BN(CH_3_)_2_, or by manipulating structural effects through the introduction of bidentate ligands, that is, PH_2_(CH_2_)_*n*_PH_2_ with *n* = 6–1, one can significantly decrease the reaction barrier for the C—X bond activation. The combination of both tuning handles causes a decrease of the C—H activation barrier from 10.4 to 4.6 kcal mol^−1^. Our activation strain and Kohn‐Sham molecular orbital analyses reveal that the electronic tuning works via optimizing the catalyst–substrate interaction by introducing a strong second backdonation interaction (i.e., “ligand‐assisted” interaction), while the mechanism for structural tuning is mainly caused by the reduction of the required activation strain because of the pre‐distortion of the catalyst. In all, we present design principles for iron‐based catalysts that mimic the favorable behavior of their well‐known palladium analogs in the bond‐activation step of cross‐coupling reactions.

## Graphical abstract

**Polishing iron!** We uncover how, and why, electronic and structural tuning‐handles of iron‐based catalysts for C—X bond activation can match, but also differ from, those of the better‐known palladium‐based analogs using quantum chemical calculations.

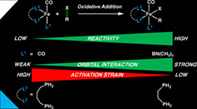

## 1 INTRODUCTION

**SCHEME 1:**
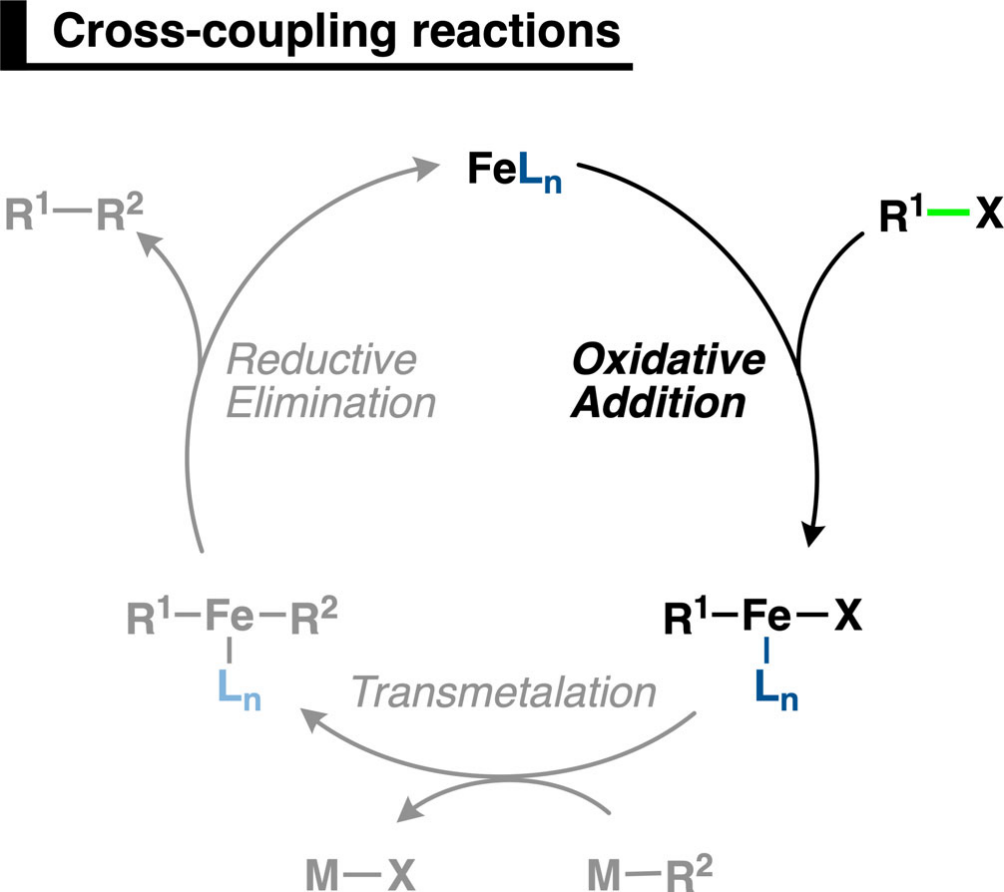
General catalytic cycle of iron‐catalyzed cross‐coupling reactions

The formation of carbon–carbon bonds by transition‐metal‐catalyzed cross‐coupling reactions is one of the pillars of modern synthetic chemistry (Scheme 1).^1^, ^2^, ^3^, ^4^ Palladium can efficiently catalyze this class of reactions with remarkable activity.^5^, ^6^ However, it also comes with several major drawbacks, for example, it is expensive, non‐sustainable, and toxic.^7^, ^8^ To overcome these disadvantages, researchers have turned to Earth‐abundant transition‐metals, such as the cheap, readily available, sustainable, and non‐toxic element, iron.^9^, ^10^

Over the years, iron‐based catalysts have drawn considerable attention from numerous research groups,^11^, ^12^, ^13^, ^14^, ^15^, ^16^, ^17^, ^18^, ^19^, ^20^, ^21^, ^22^, ^23^, ^24^, ^25^, ^26^, ^27^, ^28^, ^29^, ^30^, ^31^ nonetheless, their applicability in (synthetic) chemistry^32^ is still eclipsed by Pd‐based catalysts.^33^, ^34^, ^35^, ^36^, ^37^, ^38^, ^39^, ^40^ Recently, we have conducted a proof‐of‐concept study,^41^ in which we analyzed the complete catalytic cycle for both iron‐ and palladium‐based model catalysts. In all cases, we found that Fe‐catalysts follow a significantly lower reaction barrier for the oxidative addition. Hence, iron‐based catalysts can potentially mimic the favorable behavior of their well‐known palladium analogs in the bond‐activation step of cross‐coupling reactions.

The design of efficient catalysts usually proceeds via a trial‐and‐error approach by systematically varying the metal, ligands, reaction conditions, and solvent, in the hope of finding an optimal combination.^2^, ^42^, ^43^, ^44^, ^45^, ^46^, ^47^, ^48^, ^49^ With the ever‐increasing power of quantum‐chemical methods, chemists can now, not only, quickly compute a large set of molecular systems and chemical processes with sufficient accuracy,^50^, ^51^ but also obtain quantitative insights into the factors controlling the reactivity in these chemical systems.^41^, ^52^, ^53^ Here, we provide a quantum chemical protocol for rationally tuning the activity of iron‐d^8^‐based ^1^FeL_4_ catalysts for C—X bond activation in cross‐coupling reactions. To demonstrate our approach, we have developed a new arsenal of iron‐based catalysts that mimic the behavior of their well‐known palladium analogs.

**SCHEME 2:**
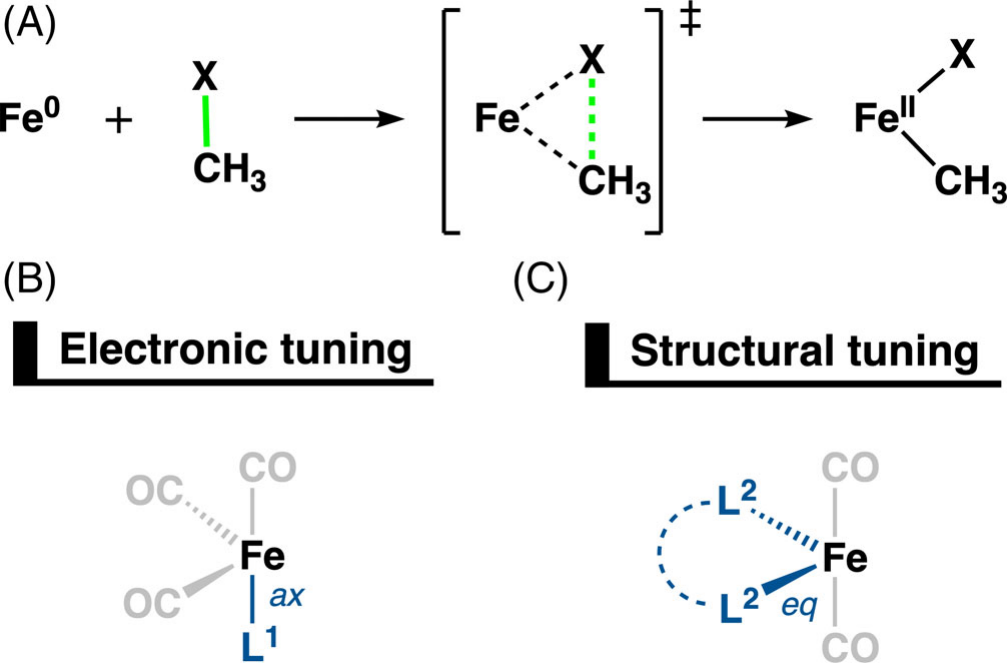
(A) Generic oxidative addition reactions of H_3_C—X (X = H, Cl, CH_3_) with singlet iron‐based catalysts. (B) Electronic tuning of ^1^Fe(CO)_3_L^1^ with L^1^ = CO, BF, PH_3_, BN(CH_3_)_2_. (C) Structural tuning of ^1^Fe(CO)_2_L^2^ with L^2^ = PH_2_(CH_2_)_*n*_PH_2_ using *n* = 6–1

To achieve this, we have explored the electronic and structural effects for the iron‐mediated C—X bond activation of model system FeCO_4_ + H_3_C—X (X = H, Cl, CH_3_) using relativistic density functional theory at ZORA‐OPBE/TZ2P. Note that the active Fe(CO)_4_ catalyst will form from the more stable but not active pre‐catalyst Fe(CO)_5_ by dissociation of one of the CO ligands. This archetypal iron catalyst is a perfect model system to derive the underlying physical factors of their properties and facilitate comparison with their palladium catalyst analogs. We have focused on the activation of the C—X bond via oxidative addition because this is an all‐important step for Pd‐based catalysts in the catalytic cycle of cross‐coupling reactions (Scheme 2A). Note that in our previous work, we found that a model iron catalyst Fe(PH_3_)_4_ goes with a higher reaction barrier for the reductive elimination step than the model palladium catalyst Pd(PH_3_)_2_ (ΔΔ*E* ^‡^ = +15.9 kcal mol^−1^ for Fe with respect to Pd at ZORA‐OPBE/TZ2P),^41^ however, the primary goal of this study is to demonstrate the tunability of the oxidative addition step and rationalize the differences between model Fe‐based and Pd‐based catalysts in cross‐coupling reactions. Ultimately, all steps of the catalytic cycle should be considered when optimizing the overall performance of a catalyst.

Electronic effects are modulated by the exchange of CO with ligands of suitable electronic properties to enhance the stabilizing interactions between the iron catalyst and substrate. Thus, we computed a series of C—X bond activations for Fe(CO)_3_L^1^ with L^1^ = CO, BF, PH_3_, BN(CH_3_)_2_ (Scheme 2B). In parallel, the structural effects of the iron‐d^8^ catalysts are tuned by the incorporation of molecular scaffolds, that is, bidentate ligands PH_2_(CH_2_)_*n*_PH_2_, denoted as P_*n*_P, with *n* = 6–1 in Fe(CO)_2_L^2^ (Scheme 2C). By combining these two tuning handles, we are able to develop highly efficient iron catalysts for C—X bond activation.

To reveal the underlying physical factors of the uncovered tuning handles, we have employed the activation strain model (ASM)^54^ in combination with Kohn‐Sham molecular orbital (KS‐MO)^55^ theory and the matching energy decomposition analysis (EDA).^56^, ^57^ This methodological approach allows the analysis of the potential energy surface and, more importantly, the activation barrier, by decomposing the total energy of the system into chemically intuitive and easily interpretable terms, proving to be valuable for providing insight into chemical reactivity.^58^, ^59^, ^60^, ^61^, ^62^, ^63^, ^64^

## 2 METHODS

### 2.1 Computational details

All density functional theory (DFT) calculations were performed using the Amsterdam Density Functional (ADF2017) software package^65^, ^66^, ^67^ and the quantum‐regions interconnected by local descriptions (QUILD) programs^68^ using relativistic density functional theory at ZORA‐OPBE/TZ2P level^69^, ^70^, ^71^, ^72^, ^73^, ^74^, ^75^, ^76^ with the frozen core approximation, set to small. Our early work^41^ and extensive benchmarking^77^, ^78^ have shown this approach to be well suited for the systems of interest. Relativistic effects were accounted for by using the zeroth‐order regular approximation (ZORA). The basis set used, denoted TZ2P, is of triple‐*ζ* quality for all atoms and has been improved by two sets of polarization functions.^77^ The accuracies of the fit scheme (Zlm fit) and the integration grid (Becke grid) were, for all calculations, set to VERYGOOD.^79^, ^80^ Geometries were optimized without any symmetry constraint. All stationary points were confirmed by vibrational analysis.^81^, ^82^, ^83^ For equilibrium structures, all normal modes have real frequencies, whereas transition states have one normal mode with an imaginary frequency. The character of the normal mode associated with the imaginary frequency was analyzed to ensure that the correct transition state was found. The potential energy surfaces (PES) of the studied reactions were obtained by performing intrinsic reaction coordinate (IRC) calculations,^84^, ^85^, ^86^ which, in turn, were analyzed using the PyFrag program.^87^, ^88^ We focused in this study on the electronic energies of the molecular systems, as our previous study showed that trends in Gibbs free activation barriers remain unchanged compared with trends in electronic energies.^41^ The optimized structures were illustrated using CYLview.^89^

### 2.2 Activation strain and energy decomposition analysis

The activation strain model (ASM) of chemical reactivity,^54^ also known as the distortion/interaction model,^90^, ^91^ is a fragment‐based approach in which the PES can be described with respect to, and understood in terms of the characteristics of the reactants. It considers the rigidity of the reactants and to which extent they need to deform during the reaction, plus their capability to interact with each other as the reaction proceeds. Using this model, one can decompose the total energy, Δ*E*(*ζ*), into the strain and interaction energy, Δ*E*_strain_(*ζ*) and Δ*E*_int_(*ζ*), respectively, and project these values onto the reaction coordinate *ζ* (Equation (1)).

(1) $$\Delta E\left(\zeta \right)=\text{∆}E_{\text{strain}}\left(\zeta \right)+\text{∆}E_{\mathrm{int}}\left(\zeta \right)$$

In this equation, the strain energy, Δ*E*_strain_(*ζ*), is the penalty that needs to be paid in order to deform the reactants from their equilibrium to the geometry they adopt during the reaction at the point *ζ* of the reaction coordinate. On the other hand, the interaction energy, Δ*E*_int_(*ζ*), accounts for all the chemical interactions that occur between these two deformed reactants along the reaction coordinate. The total strain energy can, in turn, be further decomposed into the strain energies corresponding to the deformation of the substrate, Δ*E*_strain,sub_(*ζ*), and the catalyst, Δ*E*_strain,cat_(*ζ*) (Equation (2)).

(2) $$\Delta E_{\text{strain}}\left(\zeta \right)=\text{∆}E_{\text{strain},\mathrm{sub}}\left(\zeta \right)+\text{∆}E_{\text{strain},\mathrm{cat}}\left(\zeta \right)$$

The interaction energy between the deformed reactants can be further analyzed in terms of quantitative Kohn‐Sham molecular orbital (KS‐MO)^55^ theory together with a canonical energy decomposition analysis (EDA).^56^, ^57^ The EDA decomposes the Δ*E*_int_(*ζ*) into the following three energy terms (Equation (3)):

(3) $$\Delta E_{\mathrm{int}}=\Delta V_{\text{elstat}}+\Delta E_{\text{Pauli}}+\Delta E_{\mathrm{oi}}$$

Herein, Δ*V*_elstat_(*ζ*) is the classical electrostatic interaction between the unperturbed charge distributions of the (deformed) reactants, and is usually attractive. The Pauli repulsion, Δ*E*_Pauli_(*ζ*), includes the destabilizing interaction between the fully occupied orbitals of both fragments due to the Pauli principle. The orbital interaction energy, Δ*E*_oi_(*ζ*), accounts for, among others, charge transfer between the fragments, such as HOMO–LUMO interactions.

In the herein presented activation strain and accompanied energy decomposition diagrams, the intrinsic reaction coordinate (IRC) is projected onto the C—X bond stretch. This critical reaction coordinate undergoes a well‐defined change during the reaction from the reactant (complex) via the transition state to the product and is shown to be a valid reaction coordinate for studying C—X activation reactions.^41^, ^68^, ^92^

## 3 RESULTS AND DISCUSSION

### 3.1 Ligand tuning

**FIGURE 1:**
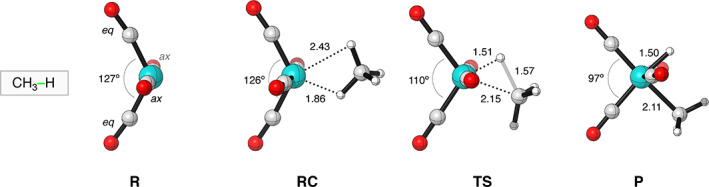
Structures and key distances (in Å) and dihedral angles (in °) of stationary points of the C—H bond activation by Fe(CO)_4_ computed at ZORA‐OPBE/TZ2P (C = gray, H = white, O = red, Fe = cyan)

First, we have investigated the influence of the electronic effects of the Fe‐catalyst through ligand variation. Table 1 and Figure 1 summarize the computed potential energy surfaces of the C—X (X = H, Cl, CH_3_) bond activation of H_3_C—X substrates by the model catalyst Fe(CO)_3_L^1^ with L^1^ = CO, BF, PH_3_, BN(CH_3_)_2_ and the previously obtained data^41^ for the Pd(CO)_2_ catalyst (see Figure S1 and Table S2 in the Supporting Information for structural information and coordinates of all computed systems). In line with previous findings,^93^, ^94^ most ligands considered, that is, L^1^ = CO, PH_3_, and BN(CH_3_)_2_, can only occupy an axial position in Fe(CO)_3_L^1^. In contrast, BF can only adopt an equatorial position.^95^ In general, the bond activation proceeds via a reactant complex (RC), followed by a transition state (TS), and a final product (P). Note, that in the gas phase, the overall reaction barrier, Δ*E* ^‡^, corresponds to the energy difference between the TS and the infinitely separated reactants.^96^ Several apparent trends can be found by analyzing the reaction profiles (Table 1). In the first place, the reaction barriers for all studied Fe‐catalysts for the C—X bond activation decrease along with the series C—C > C—Cl > C—H. For instance, for reference Fe(CO)_4_, the barrier decreases from 48.0, to 25.5, to 10.4 kcal mol^−1^ for C—C, C—Cl, and C—H, respectively. This reactivity trend is in line with other metals (e.g., palladium).^97^, ^98^, ^99^, ^100^, ^101^ Secondly, among the different ligands, BN(CH_3_)_2_ presents the lowest reaction barrier for all bonds. The largest difference is obtained for the C—H activation, going from 10.4 to 5.6 kcal mol^−1^ for L^1^ = CO and L^1^ = BN(CH_3_)_2_, respectively.

**TABLE 1:** Energies (in kcal mol^−1^) relative to reactants of the stationary points of H_3_C—X bond activation (X = H, Cl, CH_3_) by Fe(CO)_3_L^1^ with L^1^ = CO, BF, PH_3_, BN(CH_3_)_2_ ^a^

Activated bond	Fe(CO)_3_L^1^ catalyst	RC	TS	P
C—H	Fe(CO)_3_CO	−1.3	10.4	0.8
	Fe(CO)_3_BF	−2.2	11.4	5.5
	Fe(CO)_3_PH_3_	−1.3	11.5	1.1
	Fe(CO)_3_BN(CH_3_)_2_	−1.2	5.6	−8.0
	Pd(CO)_2_ ^c^	^b^	33.4	31.7
C—Cl	Fe(CO)_3_CO	−9.0	25.5	−15.1
	Fe(CO)_3_BF	−9.9	27.8	−24.8
	Fe(CO)_3_PH_3_	−6.4	25.8	−21.0
	Fe(CO)_3_BN(CH_3_)_2_	−7.6	24.4	−37.7
	Pd(CO)_2_ ^c^	^b^	34.7	5.1
C—C	Fe(CO)_3_CO	^b^	48.0	10.2
	Fe(CO)_3_BF	^b^	48.3	12.0
	Fe(CO)_3_PH_3_	^b^	50.2	10.5
	Fe(CO)_3_BN(CH_3_)_2_	^b^	47.9	−17.2
	Pd(CO)_2_ ^c^	^b^	53.9	32.8

^a^ Electronic energies computed at ZORA‐OPBE/TZ2P.

^b^ Nonexistent.

^c^ Data taken from our previous work in Sun et al.^41^

**FIGURE 2:**
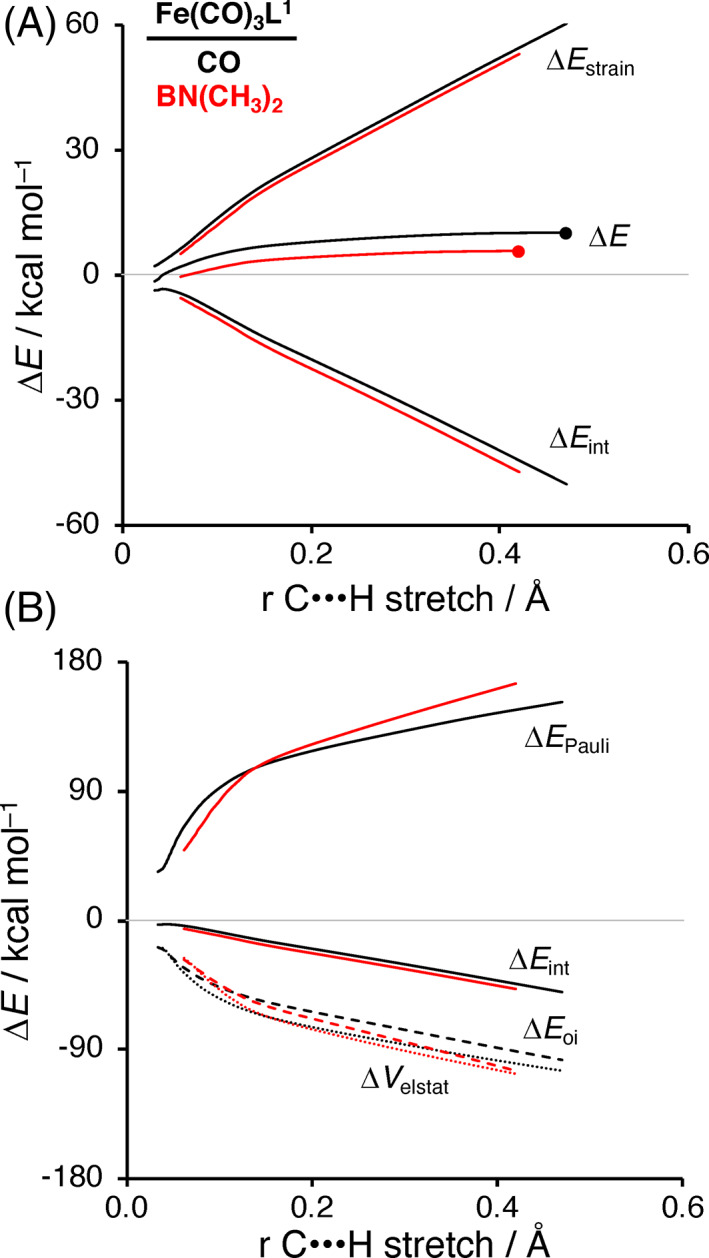
(A) Activation strain analysis and (B) energy decomposition analysis, where Δ*V*_elstat_ = dotted lines, Δ*E*_Pauli_ = solid lines, and Δ*E*_oi_ = dashed lines, for the C—H activation with Fe(CO)_3_L^1^, in which L^1^ = CO (black) and BN(CH_3_)_2_ (red), where the energy values are plotted from the reactant complex to the transition state along the IRC projected on the C**⋯**H bond stretch. Transition states are indicated with dots. Computed at ZORA‐OPBE/TZ2P

Next, we turn to the activation strain model (ASM)^54^ to gain quantitative insights into the factors controlling the reactivity of these iron‐d^8^ catalysts. The ASM decomposes the total electronic energy (Δ*E*), along the reaction coordinate, into two distinct energy terms, that is, the strain energy (Δ*E*_strain_) and the interaction energy (Δ*E*_int_). Herein, the reaction coordinate is defined as the IRC projected onto the reaction critical coordinate of the stretch in the C**⋯**X bond relative to the equilibrium distance of this bond in the original substrate.^102^ In Figure 2, we focus on the C—H bond activation by Fe(CO)_4_ as our parent model Fe‐catalyst (black curves) and Fe(CO)_3_BN(CH_3_)_2_ (red curves) as our most efficient catalyst by electronic tuning. The ASM results of the other ligands show the same characteristics and are depicted in Figure S2 and Table S1 in the Supporting Information. As can be seen in Figure 2A, the reaction barrier is significantly lower for Fe(CO)_3_BN(CH_3_)_2_ (TS is at the red dot on the red ∆*E* curve) than for Fe(CO)_4_ (TS is at the black dot on black ∆*E* curve). The lower reaction barrier for Fe(CO)_3_BN(CH_3_)_2_ mainly originates from a significantly more stabilizing interaction energy ∆*E*_int_. The required activation strain ∆*E*_strain_ is also slightly more favorable for Fe(CO)_3_BN(CH_3_)_2_, and thus, reinforces the trend that is set by ∆*E*_int_, but the difference in strain curves is less pronounced.

To get insight into why Fe(CO)_3_BN(CH_3_)_2_ achieves a more stabilizing interaction energy with the substrate than Fe(CO)_4_, we employ an energy decomposition analysis (EDA).^56^ The EDA decomposes the Δ*E*_int_ into the following three chemically intuitive energy terms: Δ*V*_elstat_, Δ*E*_Pauli_, and Δ*E*_oi_. Our canonical EDA analysis shows that Fe(CO)_3_BN(CH_3_)_2_ engages both a more stabilizing electrostatic (Δ*V*_elstat_) and orbital interaction (Δ*E*_oi_) with the substrate, in which the latter one is more important (Figure 2B). The origin of the more stabilizing orbital interaction for Fe(CO)_3_BN(CH_3_)_2_ with the substrate is further explored by performing a Kohn‐Sham molecular orbital analysis.^55^ In line with our previous work,^41^ we find that in general, Fe‐complexes feature a relatively small HOMO–LUMO gap due to the incomplete d^8^ shell of iron, which goes with both, a high‐energy d_π_ HOMO and a low‐energy d_σ_ LUMO, that can participate in both a strong π‐backdonation and σ‐donation (Figure 4B,C). This is a characteristic difference compared with, for instance, Pd‐complexes, which in general have a filled d‐shell providing also a high‐energy HOMO that can be deployed for strong π‐backdonation. However, the LUMO of Pd‐complex is a higher‐energy Pd‐5*s* derived orbital which is less capable of entering into a favorable σ‐donation interaction.^41^, ^42^

**FIGURE 3:**
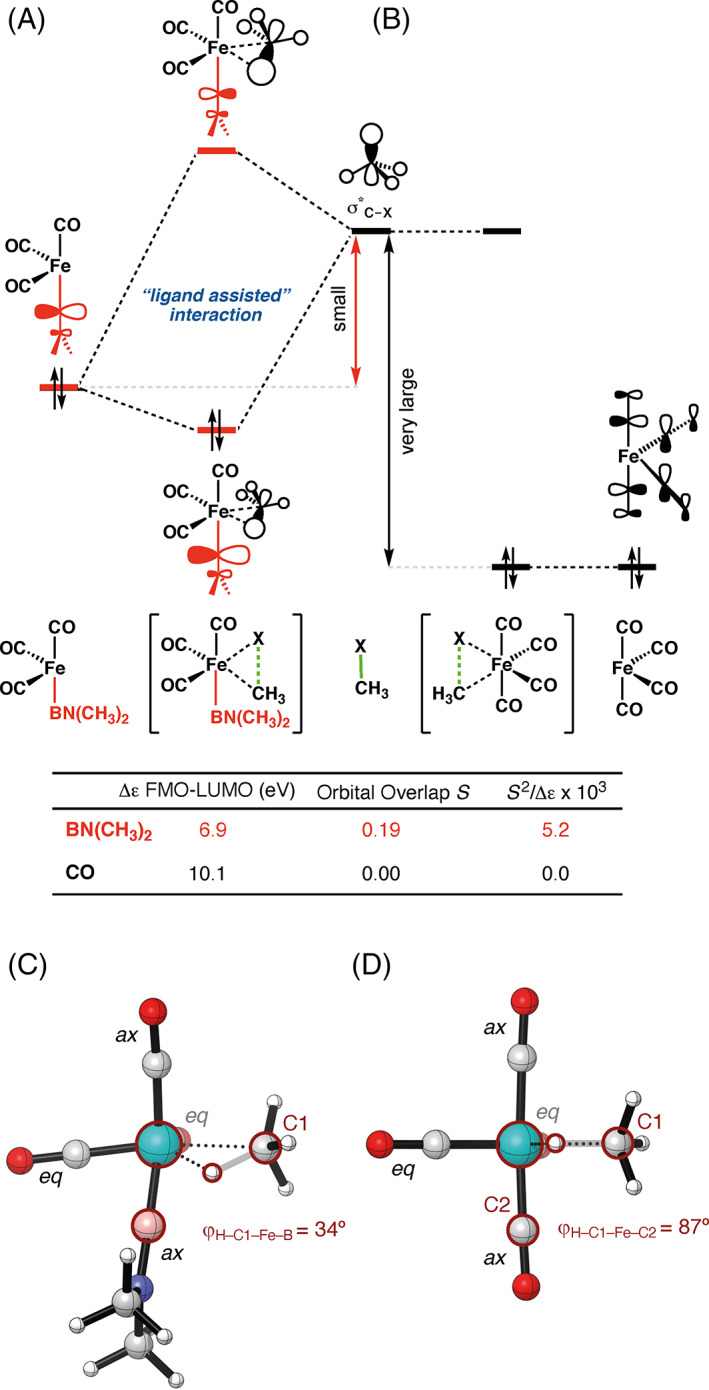
FMO diagram showing the “ligand‐assisted” interaction, which is possible for (A) L^1^ = BN(CH_3_)_2_ (red; HOMO–4) and not possible for (B) L^1^ = CO (black; HOMO–4). Computed orbital energy gaps and overlaps of the FMO of the catalyst and the LUMO of the substrate with a C—H bond stretch of 0.43 Å. Structures and key distances (in Å) and dihedral angles (in °) of Fe(CO)_3_L^1^ with (C) L^1^ = BN(CH_3_)_2_ and (D) L^1^ = CO at consistent geometries along the reaction coordinate with a C—H bond stretch of 0.43 Å. Computed at ZORA‐OPBE/TZ2P (C = gray, H = white, O = red, N = blue, B = pink, Fe = cyan)

In‐depth analyses of the catalyst–substrate interactions revealed that the Fe(CO)_3_BN(CH_3_)_2_ engages, via orbital amplitude on the BN(CH_3_)_2_ ligand, in a more stabilizing interaction with the substrate than the parent model catalyst Fe(CO)_4_. This catalyst–substrate interaction is exclusively feasible for the catalyst with the ligand BN(CH_3_)_2_ because of its high‐lying occupied orbitals (see Figure S3a). The frontier molecular orbital (FMO) of the catalyst, mainly located on the BN(CH_3_)_2_ (Figure 3A) consisting of a combination of the 2*p*_B_ and 2*p*_N_, can interact with a relatively small energy gap (Δ*ε* = 6.9 eV) with the σ*_C—X_ LUMO of the substrate. The small energy gap and the significant overlap (*S* = 0.19) are sufficient to induce a second strong backdonation from the FMO of catalyst to LUMO of the substrate, which is comparable to the classical backdonation from the 3d_π_ HOMO of the catalyst to the σ*_C—X_ LUMO of the substrate (s Table S1 for the detailed comparison). This catalyst–substrate interaction induces a skewed approach of the substrate bending it slightly toward the BN(CH_3_)_2_ ligand instead of solely to the catalytic Fe‐center (Figure 3C,D). In contrast, this “ligand‐assisted” interaction is not possible for other ligands (L^1^ = CO, BF, PH_3_), due to the substantially larger FMO_cat_–LUMO_sub_ energy gap (> 8.5 eV; Figure 3B).

**FIGURE 4:**
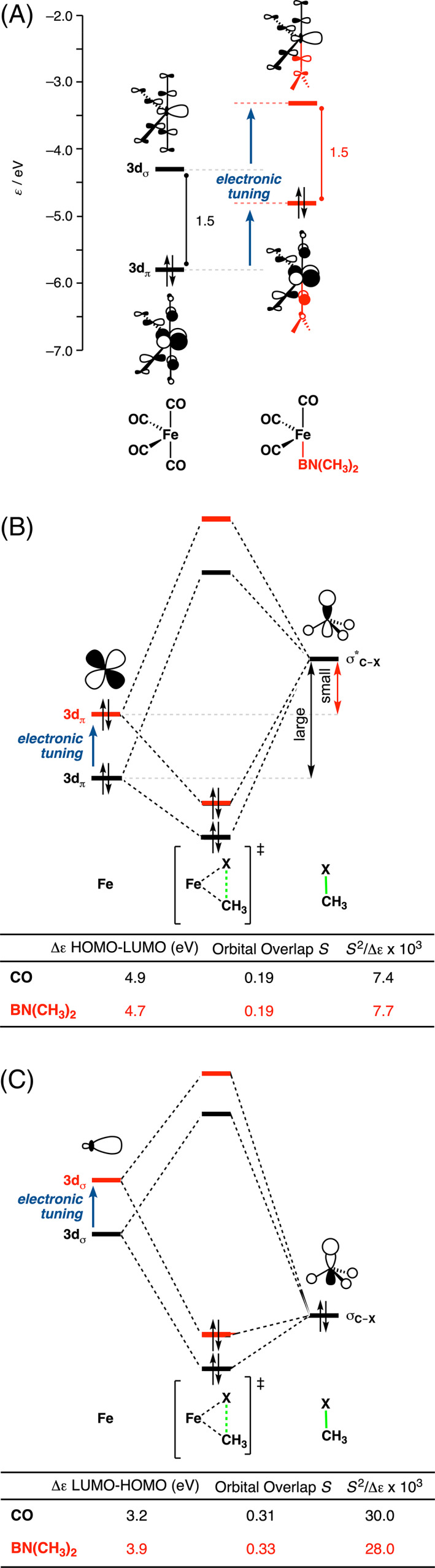
(A) FMO's of Fe(CO)_3_L^1^ with L^1^ = CO (black) and L^1^ = BN(CH_3_)_2_ (red). Catalyst–substrate FMO interaction diagrams with computed orbital energy gaps and overlaps of (B) the π‐backdonation (HOMO_cat_–LUMO_sub_) and (C) the σ‐donation (LUMO_cat_–HOMO_sub_) at consistent geometries with a C—H bond stretch of 0.43 Å. Computed at ZORA‐OPBE/TZ2P

Aside from the “ligand‐assisted” interaction, the HOMO–LUMO gap of the Fe‐catalyst is not tuned by the BN(CH_3_)_2_ ligand, which one could possibly expect due to its favorable electronic properties. Both the HOMO and the LUMO of the catalyst partake in key interactions with the substrate, that is (i) π‐backdonation from the 3d_π_ HOMO of the catalyst to the σ*_C—X_ LUMO of the substrate and (ii) σ‐donation from the σ_C—X_ HOMO of the substrate (in case of X = Cl, donation from the halogen lone‐pair also plays an important role) to the 3d_σ_ LUMO of the catalyst. However, analysis of the frontier molecular orbitals of Fe(CO)_3_L^1^ with L^1^ = CO and BN(CH_3_)_2_ show that the HOMO–LUMO gap of the Fe‐catalyst remains notably constant (Figure 4A), which is the case for all studied ligands (see Figure S3b and Table S1 for all studied catalysts). Indeed, as expected, the more electron‐donating ligand BN(CH_3_)_2_ (see Figure S3a) pushes up the orbitals more, but this electronic mechanism affects both the filled and empty orbitals (Figure 3A). This results in an enhanced π‐backdonation (Figure 4B) due to the higher‐lying HOMO orbital of FeCO_3_BN(CH_3_)_2_. In contrast, the σ‐donation (Figure 4C) becomes substantially less stabilizing due to the higher‐lying LUMO of FeCO_3_BN(CH_3_)_2_. The weaker σ‐donation cancels the enhanced π‐backdonation and, therefore, these two key interactions cannot explain the higher reactivity found for FeCO_3_BN(CH_3_)_2_.

### 3.2 Structural tuning

Next, the influence of the structural effects was systematically investigated for our iron‐d^8^ catalysts. These structural effects are dictated to a large extent by the ligand–metal–ligand angle, that is, the bite angle. In general, a smaller bite angle pre‐distorts the catalyst and reduces the required deformation of the catalyst to react, which lowers the reaction barrier. In this way, the ligands do not need to be significantly bent away from the approaching substrate, a process that occurs in linear complexes to relieve (steric) Pauli repulsion between substrate and ligands.

**FIGURE 5:**
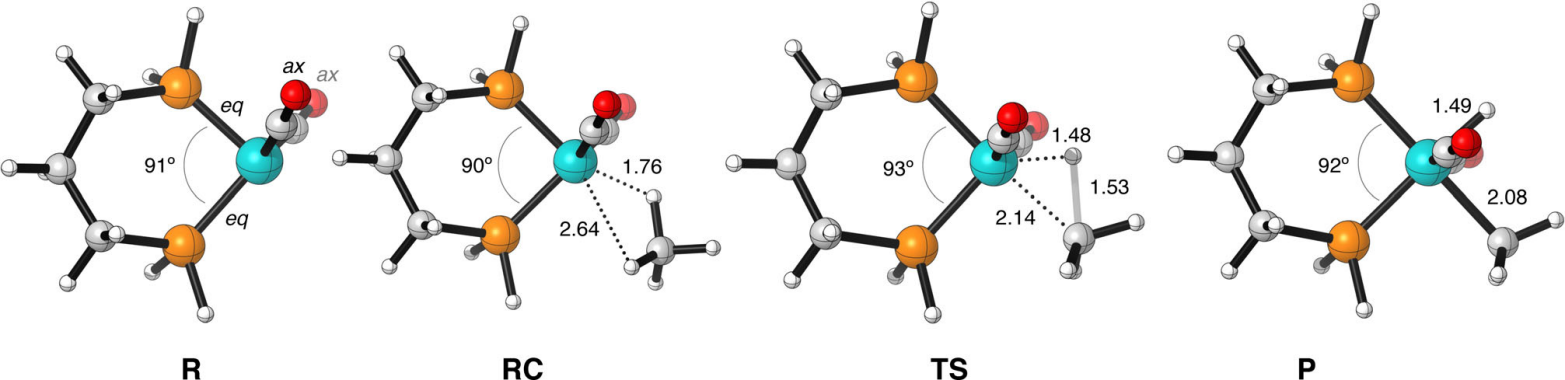
Structures and key distances (in Å) and dihedral angles (in °) of stationary points of the C—H bond activation by FeCO_2_P_3_P computed at ZORA‐OPBE/TZ2P (C = gray, H = white, O = red, Fe = cyan)

Table 2 and Figure 5 summarize the computed potential energy surfaces for C—X bond activation by d^8^‐Fe(CO)_2_L^2^ with bidentate ligands L^2^ = PH_2_(CH_2_)_*n*_PH_2_ and *n* = 6–1. These bidentate ligands adopt an equatorial position as a result of the tethered nature of these ligands. The bite angle of the parent model catalyst Fe(CO)_4_ is 127° and decreases gradually from 111° to 76° by the introduction of increasingly smaller rings in PH_2_(CH_2_)_*n*_PH_2_ from *n* = 6–1. Decreasing the *n* in PH_2_(CH_2_)_*n*_PH_2_ directly leads to a short tethered chain that pulls the two ligands closer and decreases the bite angle and effectively pre‐distorts the catalyst. Similarly with Pd catalysts, this increase of the pre‐distortion of the catalyst as the bite angle of Fe(CO)_2_P_*n*_P decreases from 111° to 91° going from *n* = 6 to *n* = 3, causes a decrease of the reaction barrier for C—H activation from 14.4 to 5.2 kcal mol^−1^, respectively. However, if the bite angle is reduced to very small values in our computed iron‐d^8^ catalysts, as in the case for Fe(CO)_2_P_2_P (87°) and Fe(CO)_2_P_1_P (76°), the reaction barrier for C—H activation does not continue to decrease, but actually begins to increase going from 5.2 to 5.4 to 10.2 kcal mol^−1^ along *n* = 3, 2, 1 (see Table 2). This remarkable finding is in sharp contrast to Pd‐based catalysts of the type d^10^‐PdL_2_, where a smaller bite angle, in general, leads to lower barriers.^103^ These reactivity trends are also found for C—Cl and C—C activation.

**TABLE 2:** Electronic energies (in kcal mol^−1^) relative to reactants for the oxidative addition by Fe(CO)_2_L^2^ (L^2^ = PH_2_(CH_2_)_*n*_PH_2_; *n* = 6–1) to C—X bonds (X = H, Cl, and CH_3_)

Activation bond	Fe‐catalyst (bite angle)^a^	RC	TS	P
C—H	Fe(CO)_2_P_6_P (111°)	−0.5	14.4	−3.8
	Fe(CO)_2_P_5_P (102°)	−0.2	8.2	−6.5
	Fe(CO)_2_P_4_P (91°)	−0.5	6.1	−6.7
	Fe(CO)_2_P_3_P (91°)	−2.7	5.2	−5.8
	Fe(CO)_2_P_2_P (87°)	−4.2	5.4	−6.3
	Fe(CO)_2_P_1_P (76°)	−3.8	10.2	−1.4
C—Cl	Fe(CO)_2_P_6_P (111°)	−6.7	27.1	−23.2
	Fe(CO)_2_P_5_P (102°)	−7.5	24.4	−26.4
	Fe(CO)_2_P_4_P (91°)	−9.0	20.5	−28.3
	Fe(CO)_2_P_3_P (91°)	−11.3	19.6	−29.4
	Fe(CO)_2_P_2_P (87°)	−12.7	21.7	−29.5
	Fe(CO)_2_P_1_P (76°)	−10.4	22.1	−24.8
C—C	Fe(CO)_2_P_6_P (111°)	−0.1	52.9	8.0
	Fe(CO)_2_P_5_P (102°)	−0.5	50.1	5.1
	Fe(CO)_2_P_4_P (91°)	−1.1	45.4	4.3
	Fe(CO)_2_P_3_P (91°)	−1.1	44.5	10.3
	Fe(CO)_2_P_2_P (87°)	−0.8	45.4	4.3
	Fe(CO)_2_P_1_P (76°)	−2.6	47.3	1.7

^a^ Computed at ZORA‐OPBE/TZ2P. P_*n*_P = PH_2_(CH_2_)_*n*_PH_2_.

**FIGURE 6:**
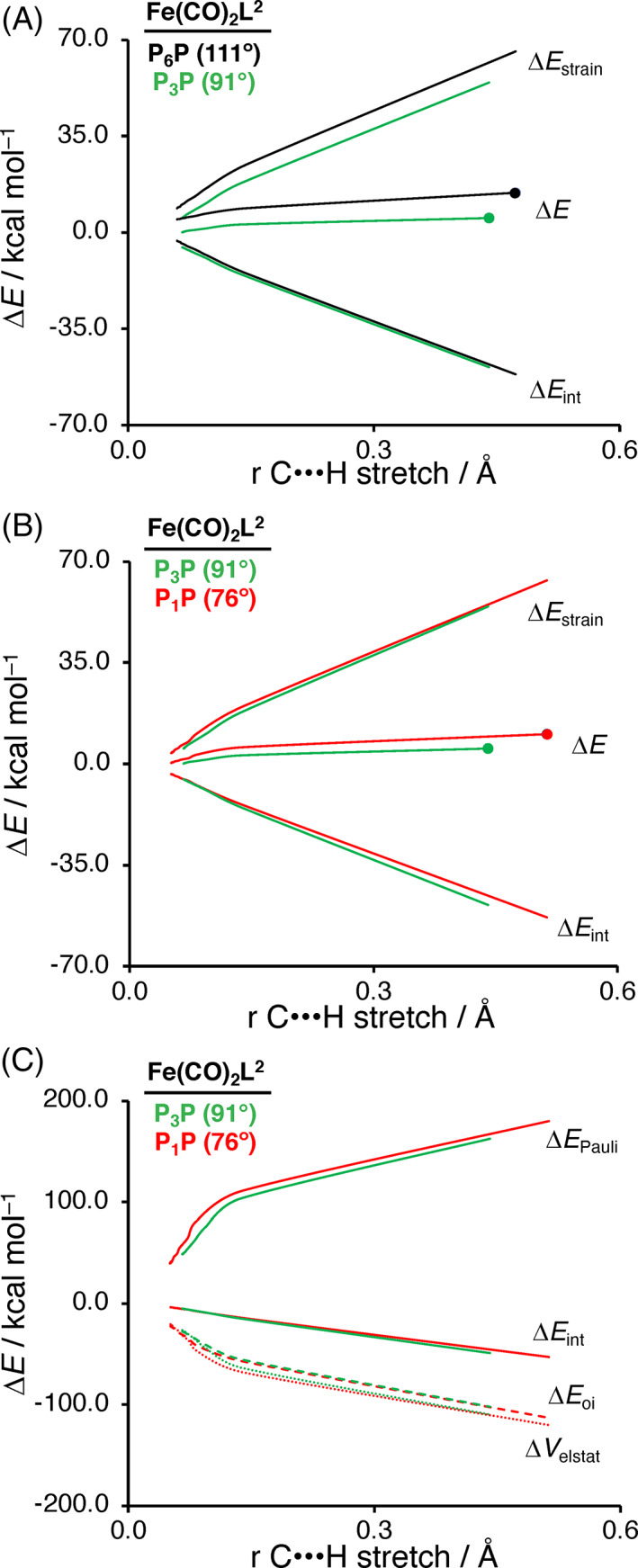
Activation strain analysis of C—H bond activation along the reaction coordinate projected onto the C**⋯**H stretch (A) by Fe(CO)_2_P_6_P (black) and Fe(CO)P_3_P (green); and (B) by Fe(CO)_2_P_3_P (green) and Fe(CO)P_1_P (red). Transition states are indicated with dots. (C) Energy decomposition analysis for the C—H bond activation by Fe(CO)P_3_P (green) and Fe(CO)P_1_P (red). Computed at ZORA‐OPBE/TZ2P

In order to understand the effects at play when decreasing the bite angle in our iron‐d^8^ catalysts, we further analyzed the PES by performing activation strain analyses (Figure 6). Figure 6A shows the ASM results for Fe(CO)_2_P_6_P with a bite angle of 111° (black) and Fe(CO)_2_P_3_P (green) with a bite angle of 91°. The ASM results for model catalysts Fe(CO)_2_P_5_P and Fe(CO)_2_P_4_P with intermediate bite angles show the same characteristics and are depicted in Figure S4 (activation strain diagrams) and Figure S5 (structures).

The reduction in reaction barrier for Fe(CO)_2_P_3_P can be solely ascribed to the less destabilizing strain, which is a direct result of the pre‐distortion of the catalyst. The pre‐distortion of the catalyst results in less deformation along the reaction path, and thus, causes a less destabilizing strain energy. In contrast, very small bite angles for our computed iron‐d^8^ catalysts do not always lead to lower barriers (Figure 6B). This seemingly counterintuitive finding can be understood by analyzing the ASM/EDA results for Fe(CO)_2_P_3_P (green) with a bite angle of 91° and Fe(CO)_2_P_1_P (red) with a bite angle of 67°. We find that for these systems the reduction in destabilizing strain energy going to smaller bite angles, that is, Fe(CO)_2_P_3_P to Fe(CO)_2_P_1_P is not present. In contrast, Fe(CO)_2_P_1_P, with the smaller bite angle, does engage in a weaker interaction with the substrate compared to Fe(CO)_2_P_3_P. The energy decomposition analysis (EDA) in Figure 6C shows that the weaker interaction energy can be traced back to the more destabilizing (steric) Pauli repulsion between the catalyst and the substrate. To verify if the EDA results are not skewed by the distance between the catalyst and the substrate, we performed an additional ASM/EDA, in which we artificially constrained the Fe**⋯**H and Fe**⋯**C bond lengths of Fe(CO)_2_P_1_P + H_3_C—H to that of Fe(CO)_2_P_3_P + H_3_C—H, while keeping the C**⋯**H bond stretch constant (see Table 3). This reinforces that, indeed, the more destabilizing (steric) Pauli repulsion between Fe(CO)_2_P_1_P and the substrate causes the weaker interaction energy compared to Fe(CO)_2_P_3_P. This can be traced back to the two CO ligands, which cannot efficiently bend away in Fe(CO)_2_P_1_P from the substrate because the bidentate ligand blocks space in the area to which they have to bend, which is illustrated by the distance between the hydrogen of the activated C—H bond and the carbon of the CO (P_3_P = 2.06 Å; P_1_P = 2.02 Å).

**TABLE 3:** Activation strain and energy decomposition analyses (in kcal mol^−1^) for the C—H bond activation by Fe(CO)_2_PH_2_(CH_2_)_1_PH_2_ and Fe(CO)_2_PH_2_(CH_2_)_3_PH_2_ ^a^

Fe‐catalyst (bite angle)	ΔE*	Δ*E*_strain_	Δ*E*_int_	Δ*V*_elstat_	Δ*E*_Pauli_	Δ*E*_oi_
Fe(CO)_2_P_3_P (91°)	3.1	49.2	−46.1	−106.7	159.0	−98.4
Fe(CO)_2_P_1_P (76°)	7.3	49.2	−41.9	−106.4	161.8	−97.3

^a^ Analyses at consistent TS‐like geometries (i.e., ΔE*) with a C**⋯**H bond stretch of 0.42 Å, a Fe**⋯**H distance of 1.49 Å, and a Fe**⋯**C distance of 2.14 Å. Computed at ZORA‐OPBE/TZ2P.

### 3.3 Rational tuning of Fe catalysts

Lastly, we combine both design handles (i.e., electronic and structural properties) for rationally optimizing the activity of a novel Fe‐catalyst for C—X bond activation. To this end, we turn to the optimal ligands identified in our analyses outlined above, in terms of barrier lowering capacity, namely BN(CH_3_)_2_ and P_3_P. Table 4 compares all results, those found above and those for the novel Fe‐catalyst, that is, Fe(CO)(BN(CH_3_)_2_)(P_3_P). This Fe‐catalyst surpasses the other model catalysts, showing that combining the favorable properties of these ligands can indeed further lower the reaction barrier, and generate a highly active iron‐d^8^ catalyst. In particular, for C—H activation, Fe(CO)BN(CH_3_)_2_(P_3_P) presents a barrier of 4.6 kcal mol^−1^, even lower than that found for either Fe(CO)_3_BN(CH_3_)_2_ (Δ*E* ^‡^ = 5.6 kcal mol^−1^) or Fe(CO)_2_(P_3_P) (Δ*E* ^‡^ = 5.2 kcal mol^−1^).

**TABLE 4:** Electronic energies (in kcal mol^−1^) relative to reactants for the oxidative insertion of d^8^‐FeL_4_ iron model catalysts into H_3_C—X bonds (X = H, Cl, CH_3_)^a^

Activation bond	Fe‐catalyst	RC	TS	P
C—H	Fe(CO)_4_	−1.3	10.4	0.8
	Fe(CO)_3_BN(CH_3_)_2_	−1.2	5.6	−8.0
	Fe(CO)_2_(P_3_P)	−2.7	5.2	−5.8
	Fe(CO)BN(CH_3_)_2_(P_3_P)	−8.4	4.6	−17.8
C—Cl	Fe(CO)_4_	−9.0	25.5	−15.1
	Fe(CO)_3_BN(CH_3_)_2_	−7.6	24.4	−37.7
	Fe(CO)_2_(P_3_P)	−11.3	19.6	−29.4
	Fe(CO)BN(CH_3_)_2_(P_3_P)	−9.6	19.0	−45.6
C—C	Fe(CO)_4_	^b^	48.0	−10.2
	Fe(CO)_3_BN(CH_3_)_2_	^b^	47.9	−17.2
	Fe(CO)_2_(P_3_P)	^b^	44.5	10.3
	Fe(CO)BN(CH_3_)(P_3_P)	^b^	44.4	−15.3

^a^ Computed at ZORA‐OPBE/TZ2P.

^b^ Nonexistent.

## 4 CONCLUSIONS

Iron‐mediated C—H, C—C, and C—Cl bond activation by our parent model iron catalyst, d^8^‐Fe(CO)_4_, can be tuned to adopt a high activity, in similar ways as the better‐known palladium‐mediated bond activation by d^10^‐Pd derivatives. However, there are also characteristic differences as follows from our relativistic DFT computations. Electronic tuning induced by the replacement of a CO ligand of d^8^‐Fe(CO)_4_ by BF, PH_3_, or BN(CH_3_)_2_ reduces the reaction barrier for C—H activation via oxidative addition from 10.4 to 5.2 kcal mol^−1^. Structural tuning through the replacement of two CO ligands by bidentate ligands PH_2_(CH_2_)_*n*_PH_2_ (*n* = 6–1) can effectively constrain the bite angle and reduce the barrier for C—H activation from 10.4 to 5.4 kcal mol^−1^. Importantly, the combination of both of these reactivity handles leads to a highly reactive catalyst that goes with a C—H activation barrier of only 4.6 kcal mol^−1^.

Our activation strain analyses reveal that electronic tuning, using our most efficient ligand, BN(CH_3_)_2_, has its origin in a “ligand‐assisted” catalyst–substrate interaction. The BN(CH_3_)_2_ ligand has a high‐energy π‐HOMO that directly contributes to a high‐energy occupied orbital in the Fe(CO)_3_BN(CH_3_)_2_ catalyst complex, still mainly localized on the BN(CH_3_)_2_ ligand, which reinforces the catalyst–substrate interaction via direct overlap and interaction with the σ*_C—X_ acceptor orbital of the substrate. This “ligand‐assisted” interaction is exclusively feasible for catalysts containing ligands with high‐lying occupied orbitals that can feature a favorably small FMO–LUMO gap with the substrate. Note that the HOMO–LUMO gap of our iron model catalysts is not tuned by the studied set of ligands. The electron‐donating ligands, that is, BF, PH_3_, and BN(CH_3_)_2_ push up both the HOMO and LUMO to a similar extent, thus keeping the HOMO–LUMO gap unchanged. This results in an enhanced π‐backdonation (HOMO_cat_–LUMO_sub_) and a weaker σ‐donation (LUMO_cat_–HOMO_sub_) for all catalysts, which practically cancel each other. Note that this “ligand‐assisted” interaction is maximized for the C—H bond activation due to the reduced steric demand of this process compared to the C—C and C—Cl bond activation.

The lowering of the reaction barrier by reducing the bite angle of the chelate complex Fe(CO)_2_(PH_2_(CH_2_)_*n*_PH_2_) catalyst works in a similar way as in the case of palladium chelate complexes: In the case of smaller bite angles, that is, as the polymethylene bridge gets shorter, ligands do not need to bend away so much to make room for the substrate during the reaction because the catalyst is already accordingly pre‐distorted. Thus, less catalyst deformation strain is building up which results in a lower barrier. Interestingly, and in contrast to the behavior of Pd‐based analogs, we find that overly bending the bite angle, to very small values, does not necessarily lead to a further lowering of C—X bond‐activation barriers for our iron‐based model catalyst. This reveals the existence of a hitherto undiscovered “sweet‐spot” in terms of bite angle for the studied iron‐based catalysts. The increase in reaction barrier upon “overbending” is the result of a more destabilizing steric (Pauli) repulsion between catalyst and substrate. This steric repulsion in the case of a too small bite angle of Fe(CO)_2_(PH_2_(CH_2_)_*n*_PH_2_) (*n* < 3) originates from the two CO ligands which can no longer efficiently bend away from the substrate because the bidentate ligand blocks space in the area to which they would otherwise bend.

## Supporting information


**Appendix S1**: Supporting Information.

## Data Availability

Additional computational results; and Cartesian coordinates, energies, and number of imaginary frequencies of all stationary points are available in the Supporting Information.
