# Supplementary material for: Rational design of iron catalysts for C–X bond activation
Source: J Comput Chem. 2022 Feb 8;44(4):495–505. doi: 10.1002/jcc.26818 (PMC10078697; doi:10.1002/jcc.26818)
Supplement: Supplementary file 1 — Appendix S1: Supporting Information. [file JCC-44-495-s001.docx]

**Contents**

**Figure S1.** Stationary points along the reaction coordinate for the oxidative insertion of Fe(CO)_3_L^1^ with L^1^ = CO, PH_3_, BF, BN(CH_3_)_2_ with key bond lengths (in Å) and angles (in º), computed at ZORA-OPBE/TZ2P.

**Figure S2.** Activation strain analysis; and energy decomposition analysis for FeCO_3_L^1^ with L = CO (black), PH_3_ (green), BF (blue), BN(CH_3_)_2_ (red) + C–H (a,b) and C–Cl (c,d) and C–C (e,f), along the IRC projected on the C•••H bond stretch. Computed at ZORA-OPBE/TZ2P (Δ*E*_Pauli_ = solid lines, Δ*E*_oi_ = dashed lines, Δ*V*_elstat_ = dotted lines).

**Figure S3.** Schematic frontier molecular orbital diagram (a) of CO, BF, PH_3_, and BN(CH_3_)_2_; and (b) of Fe(CO)_3_L^1^ with L^1^ = CO, BF, PH_3_, and BN(CH_3_)_2_. Computed at ZORA-OPBE/TZ2P.

**Figure S4.** Activation strain analysis; and energy decomposition analysis, where Δ*E*_Pauli_ = solid lines, Δ*E*_oi_ = dashed lines, Δ*V*_elstat_ = dotted lines, of the steric-tuning for FeCO_2_L^2^ with L^2^ = P_1_P (black), P_2_P (red), P_3_P (green), P_4_P (dark blue), P_5_P (light blue), P_6_P (orange) + C–H (a,b) and C–Cl (c,d) and C–C (e,f), along the IRC projected on the C•••H bond stretch. Computed at ZORA-OPBE/TZ2P.

**Figure S5.** Key stationary points (catalyst and transition state) along the reaction coordinate for the oxidative insertion of Fe(CO)_2_L^2^ with L^2^ = PH_2_(CH_2_)*_n_*PH_2_, denoted as P*_n_*P, with *n* = 1–6 with key bond angles (in °) and lengths (in Å), computed at ZORA-OPBE/TZ2P.

**Table S1.** Activation strain and energy decomposition analysis (in kcal mol^–1^) of oxidative addition of Fe(CO)_3_L^1^ with L^1^ = CO, BF, PH_3_, BN(CH_3_)_2_ to H_3_C–H.^[a]^

**Table S2.** Cartesian coordinates (in Å), energies (*E*, *H* and *G*, in kcal mol^–1^), and number of imaginary vibrational frequencies (*N*_imag_) of all stationary points and transition states in the gas phase, computed at ZORA-OPBE/TZ2P.


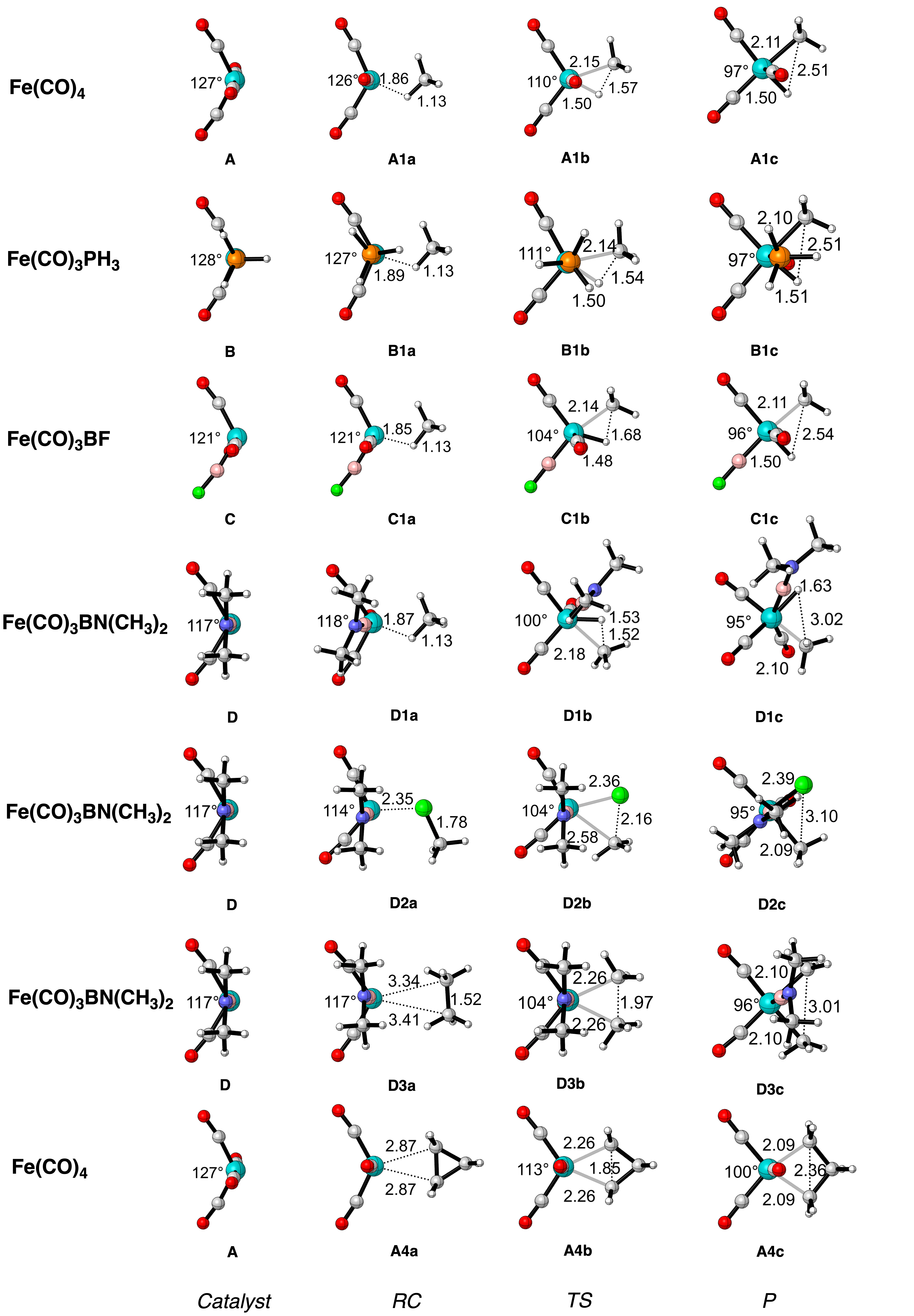


**Figure S1.** Stationary points along the reaction coordinate for the oxidative insertion of Fe(CO)_3_L^1^ with L^1^ = CO, PH_3_, BF, BN(CH_3_)_2_ with key bond lengths (in Å) and angles (in º), computed at ZORA-OPBE/TZ2P.

**
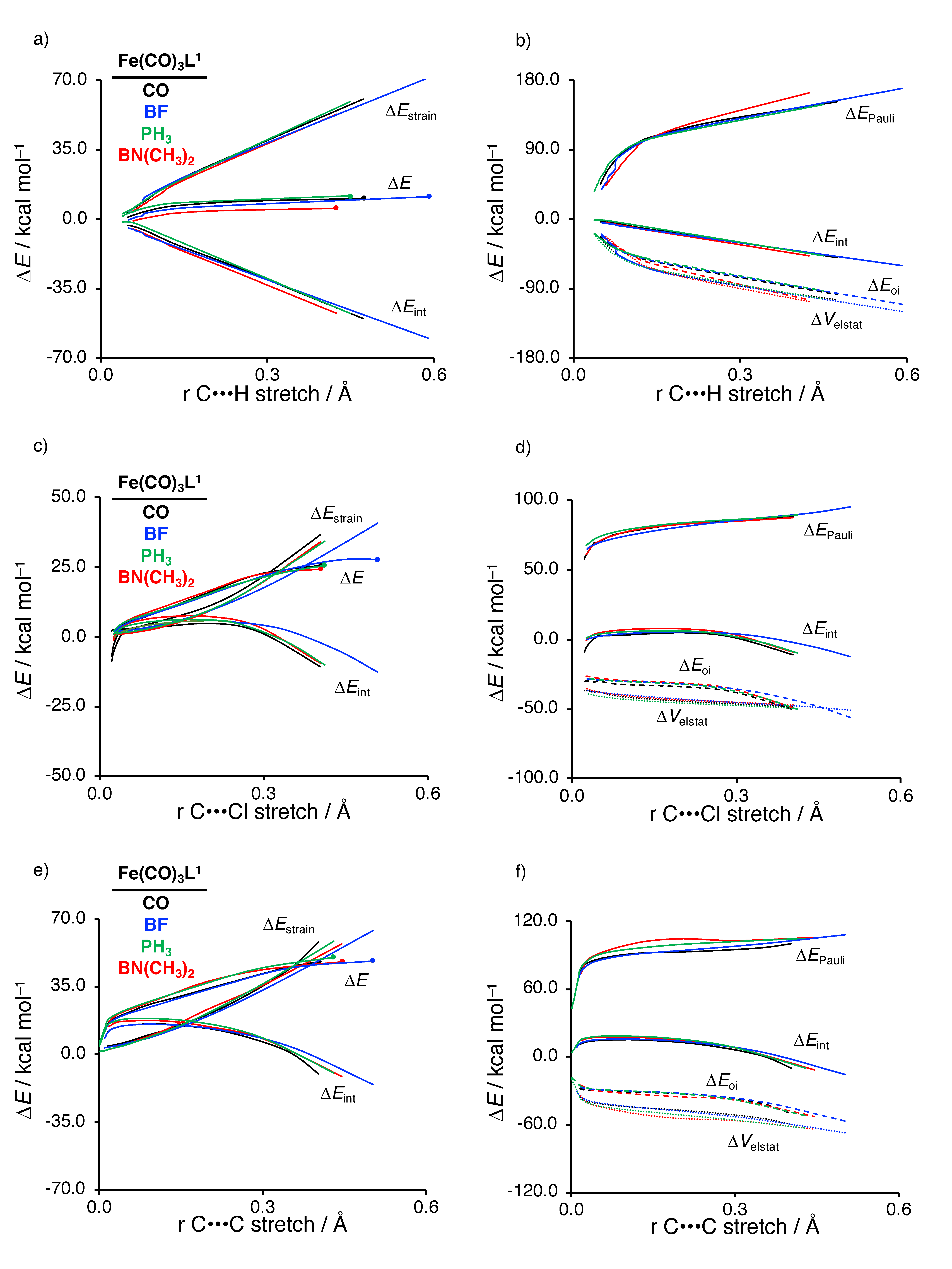
**

**Figure S2.** Activation strain analysis; and energy decomposition analysis for FeCO_3_L^1^ with L^1^ = CO (black), PH_3_ (green), BF (blue), BN(CH_3_)_2_ (red) + C–H (a,b) and C–Cl (c,d) and C–C (e,f), along the IRC projected on the C•••H bond stretch. Computed at ZORA-OPBE/TZ2P (Δ*E*_Pauli_ = solid lines, Δ*E*_oi_ = dashed lines, Δ*V*_elstat_ = dotted lines).


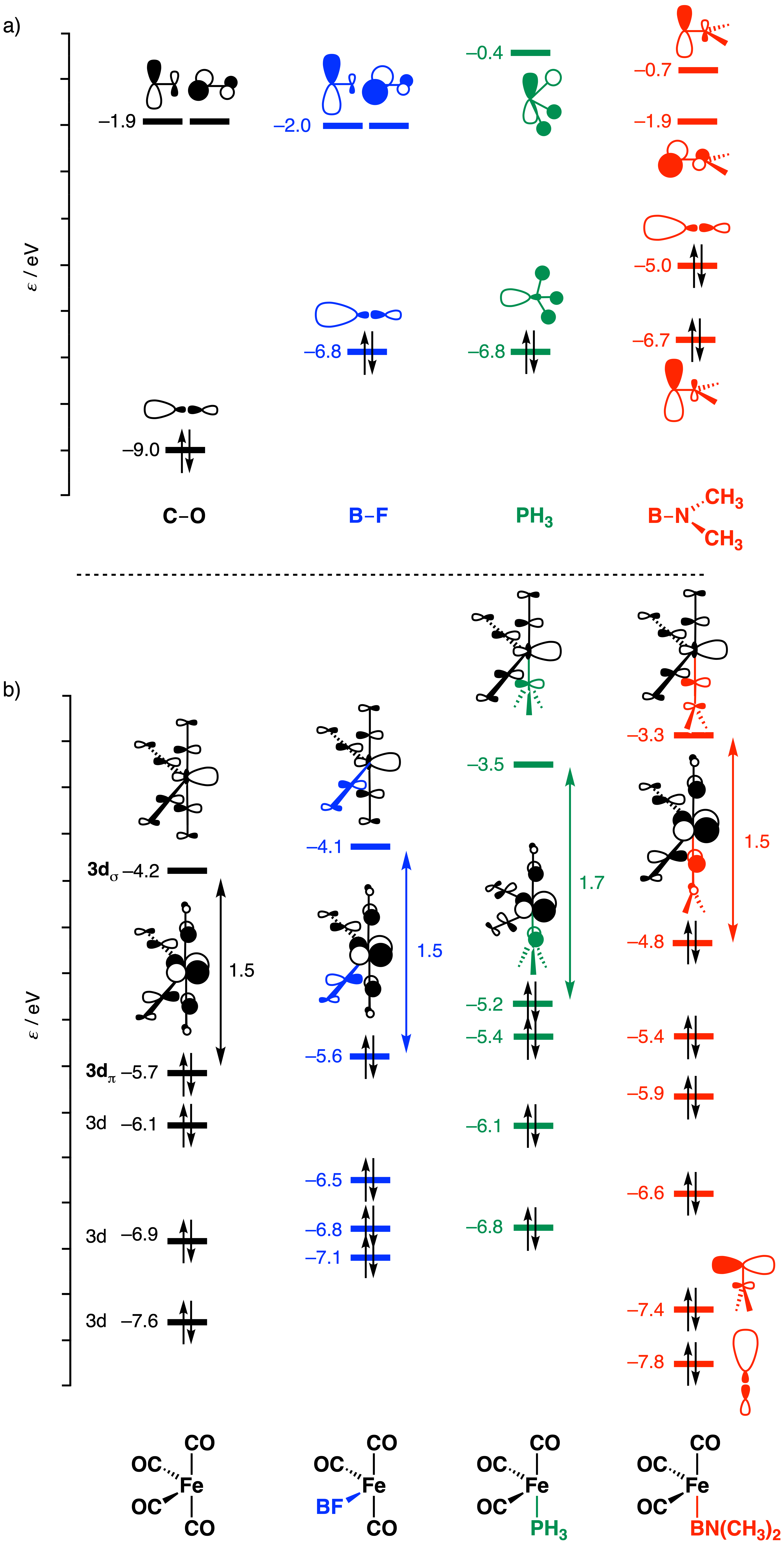


**Figure S3.** Schematic frontier molecular orbital diagram (a) of CO, BF, PH_3_, and BN(CH_3_)_2_; and (b) of Fe(CO)_3_L^1^ with L^1^ = CO, BF, PH_3_, and BN(CH_3_)_2_. Computed at ZORA-OPBE/TZ2P.

**
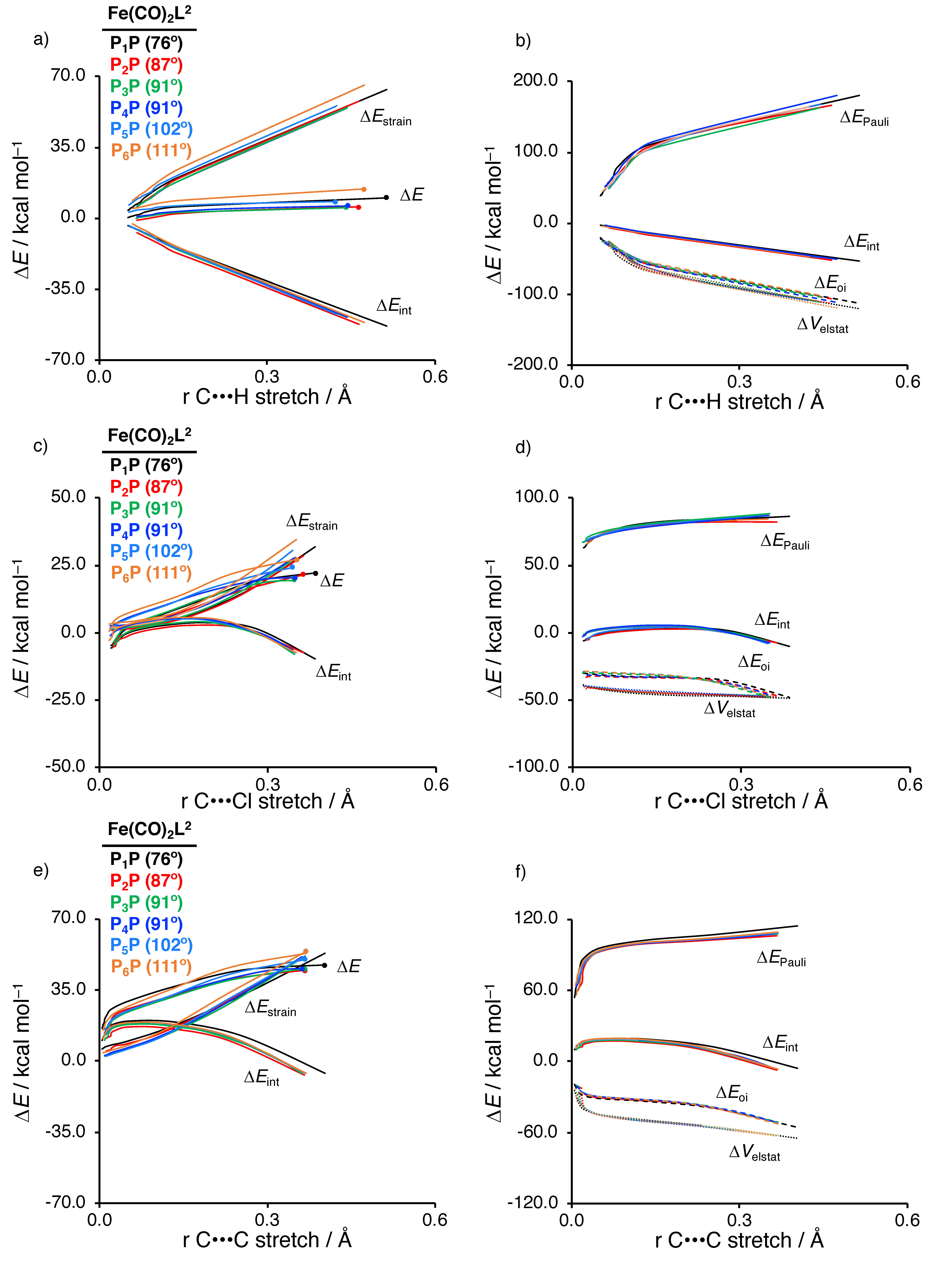
**

**Figure S4.** Activation strain analysis; and energy decomposition analysis, where Δ*E*_Pauli_ = solid lines, Δ*E*_oi_ = dashed lines, Δ*V*_elstat_ = dotted lines, of the steric-tuning for FeCO_2_L^2^ with L^2^ = P_1_P (black), P_2_P (red), P_3_P (green), P_4_P (dark blue), P_5_P (light blue), P_6_P (orange) + C–H (a,b) and C–Cl (c,d) and C–C (e,f), along the IRC projected on the C•••H bond stretch. Computed at ZORA-OPBE/TZ2P.


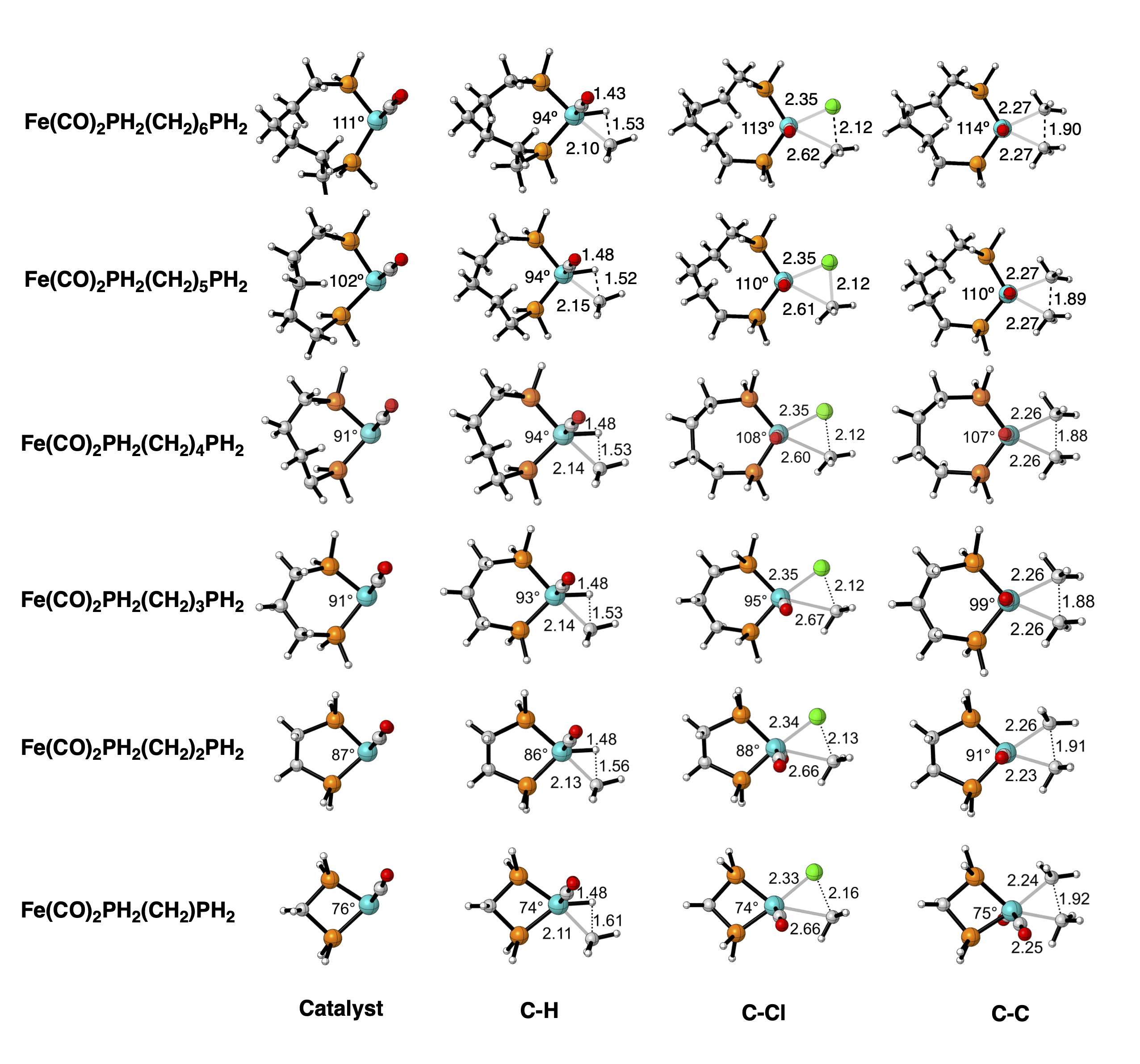


**Figure S5.** Key stationary points (catalyst and transition state) along the reaction coordinate for the oxidative insertion of Fe(CO)_2_L^2^ with L^2^ = PH_2_(CH_2_)*_n_*PH_2_, denoted as P*_n_*P, with *n* = 6–1 with key bond angles (in º) and lengths (in Å), computed at ZORA-OPBE/TZ2P.

**Table S1.** Activation strain and energy decomposition analysis (in kcal mol^–1^) of oxidative addition of Fe(CO)_3_L^1^ with L^1^ = CO, BF, PH_3_, BN(CH_3_)_2_ to H_3_C–H.^[a]^

| **Fe(CO)_3_L^1^** | **CO** | **BF** | **PH_3_** | **BN(CH_3_)_2_** |
| --- | --- | --- | --- | --- |
| Δ*E** | 9.0 | 7.7 | 8.2 | 4.9 |
| ∆*E*_strain_ | 34.4 | 32.1 | 30.7 | 32.5 |
| ∆*E*_strain,cat_ | 5.3 | 4.1 | 3.9 | 4.4 |
| ∆*E*_strain,sub_ | 29.1 | 28.0 | 26.8 | 28.1 |
| Δ*E*_int_ | –25.4 | –24.4 | –22.5 | –27.6 |
| Δ*V*_elstat_ | –81.9 | –80.0 | –77.9 | –83.5 |
| Δ*E*_oi_ | –70.2 | –67.4 | –65.2 | –76.2 |
| Δ*E*_Pauli_ | 126.7 | 123.0 | 120.6 | 131.1 |
| ***FMO energy (in eV)*** |  |  |  |  |
| LUMO_cat_ (d_σ_) | –4.6 | –4.4 | –3.8 | –3.9 |
| HOMO_sub_ (σ_C–H_) | –7.8 | –7.8 | –8.0 | –7.8 |
| Δ*ε*_σ_*_-_*_donation_ | 3.2 | 3.4 | 4.2 | 3.9 |
| HOMO_cat_ (d_π_) | –5.3 | –5.3 | –4.6 | –5.1 |
| LUMO_sub_ (σ^*^_C–H_) | –0.4 | –0.3 | –0.4 | –0.4 |
| Δ*ε*_π-backdonation_ | 4.9 | 5.0 | 4.2 | 4.7 |
| FMO_cat_ (p_π_) | –10.5 | –9.6 | –8.9 | –7.3 |
| LUMO_sub_ (σ^*^_C–H_) | –0.4 | –0.3 | –0.4 | –0.4 |
| Δ*ε*_π-backdonation_ | 10.1 | 9.3 | 8.5 | 6.9 |
| ***FMO overlap*** |  |  |  |  |
| < Fe: d_σ_ \| CH_4_: σ_C–H_ > | 0.31 | 0.32 | 0.31 | 0.33 |
| < Fe: d_π_ \| CH_4_: σ^*^_C–H_ > | 0.19 | 0.20 | 0.17 | 0.19 |
| < Fe: p_π_ \| CH_4_: σ^*^_C–H_ > | 0.00 | 0.03 | 0.01 | 0.19 |
| ***S^2^/Δε x 10^3^*** |  |  |  |  |
| < Fe: d_σ_ \| CH_4_: σ_C–H_ > | 30.0 | 30.1 | 22.9 | 28.0 |
| < Fe: d_π_ \| CH_4_: σ^*^_C–H_ > | 7.4 | 8.0 | 6.9 | 7.7 |
| < Fe: p_π_ \| CH_4_: σ^*^_C–H_ > | 0.0 | 0.0 | 0.0 | 5.2 |

[a] Analyses at consistent geometries with a C–H bond stretch of 0.43 Å. Computed at ZORA-OPBE/TZ2P.

**Table S2.** Cartesian coordinates (in Å), energies (*E*, *H* and *G*, in kcal mol^–1^), and number of imaginary vibrational frequencies (*N*_imag_) of all stationary points and transition states in the gas phase, computed at ZORA-OPBE/TZ2P.

**R: Fe(CO)_4_**

***E*** = -1655.71

**H** = -1627.96

**G** = -1657.03

***N*_imag_** = 0

Fe 0.00000000 0.00000000 0.00266480

O -2.92700731 0.00000000 0.08271546

C -1.77694389 0.00000000 0.02975038

O 0.00000000 2.48259819 1.46467953

O 2.92700731 0.00000000 0.08271546

O 0.00000000 -2.48259819 1.46467953

C 0.00000000 -1.54757822 0.78082679

C 1.77694389 0.00000000 0.02975038

C 0.00000000 1.54757822 0.78082679

**R: Fe(CO)_3_PH_3_**

***E*** = -1657.65

**H** = -1619.39

**G** = -1649.40

***N*_imag_** = 0

Fe -0.04538500 0.05723600 -0.17538900

O -0.11405800 -2.82151800 -0.22689000

C -0.07562000 -1.66174200 -0.32069800

O -2.93770000 0.10068800 -0.26601300

C -1.78057400 0.08930800 -0.23953900

O -0.12522000 1.47583100 2.32970300

C -0.08359200 1.00477600 1.26585300

H 2.72973900 0.74295000 -1.30379100

P 2.14035500 0.07507100 -0.18852400

H 2.89956400 0.71320300 0.82843800

H 2.90911400 -1.11716300 -0.26257700

**R: Fe(CO)_3_BF**

***E*** = -1543.14

**H** = -1516.45

**G** = -1546.09

***N*_imag_** = 0

Fe 0.05493700 0.00532000 0.03276400

O -1.15353900 -2.62437300 -0.02043800

C -0.55823900 -1.63240900 0.00713000

O -0.15677800 0.55551000 -2.82413500

F -1.95896700 2.24303100 -0.06476700

O -0.40302900 0.56628600 2.85916700

C -0.17167200 0.32680200 1.74897800

B -1.01679100 1.36215200 -0.01856700

C -0.02197000 0.32048100 -1.69713900

**R: Fe(CO)_3_BN(CH_3_)_2_**

***E*** = -2546.23

**H** = -2468.89

**G** = -2502.70

***N*_imag_** = 0

Fe 0.55094900 -0.00366400 0.17098000

C 0.32561800 -1.11067300 -1.11004700

B -1.26683000 -0.00252300 0.08975800

O 0.16709800 -1.71761400 -2.09561600

O 3.49737300 -0.00284500 0.02660200

O 0.22395700 2.73861700 -0.60730200

C 0.36451000 1.65973400 -0.18475300

C 2.34590900 -0.01506900 0.12241100

N -2.63200200 0.01721500 -0.05192700

H -2.69490900 -1.95978300 -0.74803600

H -4.13692300 0.95236600 1.08310600

C -3.41692700 1.19018000 0.28890700

H -2.76346200 1.99257200 0.63822000

H -3.96886300 1.54779100 -0.59022800

C -3.37618300 -1.13562700 -0.52645600

H -4.09553500 -1.46764600 0.23360500

H -3.92528400 -0.88225800 -1.44277400

**R: Fe(CO)_2_(PH_2_CH_2_PH_2_)**

***E*** = -1857.89

**H** = -1802.26

**G** = -1832.44

***N*_imag_** = 0

C -2.35287926 -0.88718470 0.19730642

P -1.11221732 -0.49817727 1.53905298

P -1.05247294 -0.61143392 -1.05700454

O 0.06084693 2.74295517 -1.17743399

H -1.14662742 -1.70363991 2.31347519

Fe 0.42206611 0.17415210 0.13702017

C 1.60420840 -0.90482724 -0.55633005

O 2.33824285 -1.61904303 -1.11611237

H -1.65848180 0.11025149 -2.12944935

H -1.89350643 0.24246117 2.48668164

H -0.90496113 -1.85951646 -1.73047615

C 0.22902566 1.75405192 -0.58085921

H -2.79776718 -1.88689329 0.23240562

H -3.13340154 -0.12161017 0.08608688

**R: Fe(CO)_2_(PH_2_CH_2_CH_2_PH_2_)**

***E*** = -2247.63

**H** = -2173.06

**G** = -2204.83

***N*_imag_** = 0

Fe 6.81788611 9.46583731 3.19940718

P 5.20453260 9.28914101 1.93470011

P 8.04893402 8.77882226 1.55075650

O 6.18848166 12.30515997 3.37509308

H 4.01074067 8.63685785 2.35427952

H 7.45574751 8.02305634 -0.72945560

H 7.06966391 9.72981011 -0.43443863

O 5.48445674 7.51186078 4.89557565

H 9.21185181 9.43775640 1.03883117

H 4.59220568 10.46883864 1.41843586

H 8.64932073 7.47815341 1.49843987

C 5.60561313 8.35998180 0.38542121

H 5.52709697 7.29481324 0.63587136

H 4.87300565 8.58220702 -0.40012459

C 7.02950434 8.72252861 -0.00289349

C 6.07039941 8.27382301 4.23116840

C 6.50074654 11.18156121 3.31485025

**R: Fe(CO)_2_(PH_2_CH_2_CH_2_CH_2_PH_2_)**

***E*** = -2627.73

**H** = -2534.68

**G** = -2568.05

***N*_imag_** = 0

H -2.41190673 1.76619436 -0.76498607

P -0.28869666 -0.01365380 1.65414371

P -1.16234606 -0.22966812 -1.20453770

O 0.81329237 2.58576268 -1.65426764

H -0.67086976 -1.21536559 2.33385553

H -3.88509571 0.98660418 1.11645379

H -3.17340250 -0.61104811 1.03495277

O 1.50240366 -2.42186683 -1.62144610

H -1.27170746 0.05965389 -2.59721244

H 0.43116101 0.52342246 2.76979869

H -1.71060991 -1.54636395 -1.25286281

C -2.95219725 0.45506371 0.88324540

H -2.23838527 0.77018584 2.90187228

H -1.64844656 1.96674125 1.73419456

C -2.61827590 0.70398533 -0.58403944

C 1.19421153 -1.45465613 -1.04185030

C 0.77395874 1.58185583 -1.05389762

Fe 0.65631955 0.02327439 -0.28069248

C -1.88046375 0.90366378 1.87457678

H -3.46614406 0.41995351 -1.22200355

**R: Fe(CO)_2_(PH_2_CH_2_CH_2_CH_2_CH_2_PH_2_)**

***E*** = -3005.35

**H** = -2891.37

**G** = -2926.85

***N*_imag_** = 0

H -2.31694400 1.52219900 -1.00138200

P 0.08069300 -0.34829400 1.89381700

P -0.97652500 -0.45591500 -0.91227400

O 0.73508800 2.55934900 -1.47961900

H -0.55945900 -1.59697200 2.15116100

H -3.95555900 1.10227700 0.84076000

H -3.10584900 -0.34212700 1.31599400

O 1.87907500 -2.29613200 -1.53907300

H -1.06926200 -0.49939600 -2.33216600

H 1.02412700 -0.48607000 2.95853300

H -1.47523500 -1.76702100 -0.65820500

C -2.95398600 0.65475400 0.88033900

H -2.72376100 2.14351500 2.42195000

H -1.48528900 2.21162400 1.18620000

C -2.49063500 0.52813700 -0.57138300

C 1.49845100 -1.38544500 -0.91213100

C 0.79411900 1.56367800 -0.86655100

Fe 0.85220800 0.00474100 -0.08457000

C -2.07307100 1.51732100 1.79747400

H -3.27756300 0.05352500 -1.17551500

C -1.13007800 0.77708000 2.74017800

H -0.52831200 1.51321900 3.28774000

H -1.68746700 0.19975500 3.48855000

**R: Fe(CO)_2_(PH_2_CH_2_CH_2_CH_2_CH_2_CH_2_PH_2_)**

***E*** = -3381.36

**H** = -3248.97

**G** = -3286.71

***N*_imag_** = 0

H -2.90438800 0.86311700 -1.52478000

P 0.05399000 0.01306000 1.75266400

P -1.05804200 -0.68473300 -1.28129100

O 0.03539800 2.58765100 -1.95431700

H -0.29551900 -1.24100000 2.33392800

H -4.30139500 -0.31039700 0.46958900

H -2.74618800 -0.91146600 0.95487300

O 2.09099500 -1.97984800 -1.50218900

H -1.03825600 -0.77940900 -2.70054500

H 1.26039800 0.16298200 2.50568000

H -1.24792500 -2.07085300 -1.01129000

C -3.22838600 -0.08433900 0.41804400

H -3.86696600 1.85443600 1.10385600

H -2.19559000 1.81126600 0.59635800

C -2.79285800 -0.11751200 -1.04654000

C 1.48169200 -1.11404900 -1.00230800

C 0.23860800 1.66537200 -1.26136500

Fe 0.56041700 0.20798400 -0.36049700

C -2.95675800 1.24377100 1.14195100

H -3.39835700 -0.82967600 -1.62535500

C -2.50614300 1.09123600 2.59764700

C -0.99295700 1.12852500 2.80912400

H -2.91855800 0.16557900 3.02185700

H -2.93111500 1.90272600 3.20396800

H -0.75582100 0.92481400 3.86107400

H -0.61954100 2.13764600 2.58402500

**R: Fe(CO)_2_(PH_2_CH_2_CH_2_CH_2_CH_2_CH_2_CH_2_PH_2_)**

***E*** = -3755.19

**H** = -3604.42

**G** = -3644.58

***N*_imag_** = 0

H -1.81967300 1.78959100 -1.11655500

P 0.22771100 -0.21065000 1.90477600

P -1.12848600 -0.48595700 -1.33099600

H -3.91725800 0.08484700 0.25774400

H -0.25273800 -1.21809900 2.79052600

H -2.33801400 0.06607700 0.98249500

H -4.15841400 2.48734400 0.64053800

C 1.12280200 0.79759900 -1.21660300

H -1.02997500 -0.41792500 -2.74794600

H 1.50592800 -0.11612900 2.53951600

H -1.97135200 -1.63169800 -1.26637600

C -3.00238300 0.68178800 0.36437500

H -3.78511800 1.61138800 2.09799300

H -0.37046500 2.11213700 0.72901000

C -2.36192700 0.84436400 -1.01575600

C 0.79166100 -2.20539800 -0.71240500

Fe 0.63106400 -0.54025500 -0.23579900

O 0.91479200 -3.29186700 -1.12743100

C -0.80708100 2.42136000 1.68704500

H -3.10614800 0.81422100 -1.82220800

C -0.64950600 1.25507500 2.66751600

H -0.10391500 1.54330000 3.57210400

H -1.61659700 0.87414500 3.00995500

H -0.18597200 3.26243000 2.01900200

C -2.22238000 2.95470200 1.43956200

H -2.54530100 3.56184200 2.29670500

O 1.49809700 1.62418900 -1.95866000

H -2.14478800 3.66474100 0.60508500

C -3.34687700 1.94741400 1.14818500

**R: Fe(CO)_2_BN(CH_3_)_2_(PH_2_CH_2_CH_2_CH_2_PH_2_)**

***E*** = -3507.31

**H** = -3361.59

**G** = -3398.87

***N*_imag_** = 0

H 1.31169100 -2.46835500 1.52710300

P 2.23942500 -0.75143000 -1.68745900

P 2.51201500 -0.37514800 1.46137100

C 3.47638900 3.19293500 -1.98373100

H 1.53241700 0.36865800 -2.21703400

H -0.79759700 -1.80986100 0.30817100

H -0.15636100 -0.20077000 0.05856600

H 3.68344900 2.98292500 1.37998200

H 2.90844500 -0.66780900 2.81131200

H 2.41398100 -1.35560000 -2.97913600

H 1.88540100 0.85256900 1.82895200

C 0.14048900 -1.25518400 0.16106100

H 0.01461300 -1.70457400 -1.95149500

H 1.09483300 -2.77999000 -1.05358300

C 0.98246800 -1.42652800 1.42568600

H 4.35598300 3.82850100 -2.16101500

H 3.45549100 2.41303200 -2.74761700

N 3.53310200 2.59007800 -0.66702900

C 0.76340700 -1.73824800 -1.14913100

H 0.37762800 -1.18688500 2.31007400

Fe 3.64822400 -0.59055000 -0.22252600

H 2.72495900 4.17487700 0.46653500

B 3.57846200 1.21683500 -0.47849700

H 4.50054200 4.16976900 0.33842700

O 4.03372600 -3.48883500 0.06796600

H 2.57745000 3.81730300 -2.09112500

C 3.61350500 3.52706200 0.43570200

C 3.87273900 -2.33491900 -0.04724200

**R: CH_4_**

***E*** = -554.09

**H** = -524.26

**G** = -537.54

***N*_imag_** = 0

C 0.00000000 0.00000000 0.00000000

H 0.63276400 -0.63276400 0.63276400

H -0.63276400 -0.63276400 -0.63276400

H -0.63276400 0.63276400 0.63276400

H 0.63276400 0.63276400 -0.63276400

**R: CH_3_Cl**

***E*** = -516.57

**H** = -490.94

**G** = -507.61

***N*_imag_** = 0

C 0.00000000 0.00000000 -2.00508000

Cl 0.00000000 0.00000000 -0.23687800

H -0.51699900 -0.89546800 -2.35691600

H -0.51699900 0.89546800 -2.35691600

H 1.03399700 0.00000000 -2.35691600

**R: C_2_H_6_**

**E** = -933.49

**H** = -884.75

**G** = -900.99

***N*_imag_** = 0

C 0.00000000 0.00000000 -0.76004500

C 0.00000000 0.00000000 0.76004500

H -0.00001600 -1.02019200 -1.16239400

H -0.88350400 0.51011000 -1.16239400

H 0.88352000 0.51008200 -1.16239400

H 0.88352000 -0.51008200 1.16239400

H -0.88350400 -0.51011000 1.16239400

H -0.00001600 1.02019200 1.16239400

**R: C_3_H_6_**

***E*** = -1120.20

**H** = -1067.37

**G** = -1084.94

***N*_imag_** = 0

C -0.11251682 2.88305762 -0.44743193

C -0.14886937 2.28027284 -1.81968870

C -0.19361149 3.76971887 -1.65351974

H -1.05870955 1.77362920 -2.13089157

H 0.76486174 1.83691306 -2.20693578

H 0.82603789 2.85019082 0.09984202

H -0.99776584 2.78677418 0.17586813

H 0.68984441 4.34035623 -1.92790690

H -1.13399599 4.27727367 -1.85187161

**R: Fe(CO)_4_ (90°)**

***E*** = -1644.12

**H** = -1617.29

**G** = -1645.28

***N*_imag_** = 0

Fe 0.00074353 0.00500332 -0.53920926

O -0.00865072 -1.79718585 1.67834010

C -0.00427034 -1.23318437 0.66664769

O 0.00383734 1.74952987 1.72352990

O -2.92858982 0.02217343 -0.74639910

O 2.93051863 0.00560767 -0.74163158

C 1.78016397 0.00597806 -0.65515445

C -1.77834417 0.01530683 -0.65858804

C 0.00209356 1.21028379 0.69838950

**R: Fe(CO)_4_ (95°)**

***E*** = -1647.52

**H** = -1620.61

**G** = -1648.60

***N*_imag_** = 0

Fe 0.00009221 -0.00083406 0.52061160

O 0.00931158 -1.87558918 -1.63535033

C 0.00542898 -1.26813074 -0.64890683

O -0.01002101 1.88722738 -1.62386402

O 2.93024355 0.00675680 0.70037638

O -2.92990495 -0.01640931 0.70191273

C -1.77915134 -0.01053989 0.62706820

C 1.77943170 0.00348293 0.62630223

C -0.00601189 1.27509097 -0.64030658

**R: Fe(CO)_4_ (100°)**

***E*** = -1650.07

**H** = -1622.68

**G** = -1652.50

***N*_imag_** = 0

Fe -0.00859177 0.13242915 -0.10953047

O 0.05266388 -2.60004446 -0.96417976

C 0.02481796 -1.45466779 -0.79156063

O 0.05312916 -0.79823920 2.59687768

O -2.94393489 0.23235153 -0.14046124

O 2.91543273 0.38952088 -0.21800305

C 1.76858896 0.28171516 -0.17259631

C -1.79219103 0.18617143 -0.12527068

C 0.02437762 -0.26239006 1.56988386

**RC: Fe(CO)_4_ + CH_4_**

***E*** = –2211.13

**H** = -2152.64

**G** = -2186.74

***N*_imag_** = 0

Fe 0.35780891 0.03825438 0.48752113

O -1.43802173 -1.12505914 -1.45217119

C -0.66267593 -0.72030439 -0.69282048

O -0.42629797 2.40938870 1.94586288

O 1.31561351 1.80643037 -1.63395533

O -1.02975892 -1.52409692 2.53166218

C -0.45657654 -0.92268086 1.73313339

C 0.96585099 1.09867994 -0.79455246

C -0.03387049 1.46193516 1.40712795

C 2.63288174 -0.90589212 1.00999077

H 3.49023136 -0.75012839 0.35348151

H 2.76932847 -1.78794449 1.63748323

H 2.49816316 -0.02749979 1.64450324

H 1.77347037 -1.16072056 0.32248459

**TS: Fe(CO)_4_ + CH_4_**

***E*** = -2199.43

**H** = -2142.93

**G** = -2175.56

***N*_imag_** = 1, -751.90

Fe 0.34088938 0.01304017 0.46047141

O -1.75076143 -0.80080398 -1.37625394

C -0.92335517 -0.47287970 -0.64254083

O -0.49825553 2.41831727 1.90230645

O 1.69917876 1.48348141 -1.65721901

O -0.63146929 -1.83766027 2.49088409

C -0.26352657 -1.09418265 1.69324896

C 1.14917883 0.91693750 -0.82001466

C -0.16094089 1.47030656 1.33983353

C 2.30114584 -0.40453727 1.22610154

H 3.11113121 -0.34009600 0.49583177

H 2.39620583 -1.34546235 1.77286522

H 2.39720271 0.42297797 1.93088228

H 1.34784361 -1.05951162 0.16991896

**P: Fe(CO)_4_ + CH_4_**

***E*** = -2208.97

**H** = -2151.50

**G** = -2183.59

***N*_imag_** = 0

Fe 0.29982211 -0.00309893 0.42404388

O -2.07990493 -0.37020876 -1.20397145

C -1.13897130 -0.22101296 -0.55807796

O -0.56545804 2.40898995 1.83351285

O 2.02676697 1.02740617 -1.66830284

O -0.22973443 -2.13374035 2.32203604

C -0.05907275 -1.26273353 1.59062569

C 1.31518802 0.66254168 -0.84171635

C -0.23953997 1.45977257 1.27227726

C 2.07286263 0.15490377 1.54879572

H 2.55967332 1.11154984 1.34507853

H 2.73999583 -0.66394612 1.27566563

H 1.84296859 0.10398148 2.61564658

H 0.91467813 -1.22524559 -0.19518736

**RC: Fe(CO)_4_ + CH_3_Cl**

***E*** = -2181.31

**H** = -2126.49

**G** = -2163.02

***N*_imag_** = 0

Fe -0.01936064 -0.15862999 -0.26387674

O -0.05035324 -0.34955885 -3.14878138

C -0.02973485 -0.20042048 -2.00013474

O -0.28789361 -2.56497274 1.31632131

O -2.93782201 -0.03451368 -0.35282162

O 2.85057573 -0.69917106 -0.37188667

C 1.72388824 -0.45826832 -0.31687186

C -1.78551936 -0.05560673 -0.30540754

C -0.17602063 -1.56121316 0.74657031

C 0.23109620 1.97471388 2.43450193

H 0.35118661 3.01126695 2.75725060

H 1.06653635 1.35913434 2.76977676

H -0.72146230 1.56535314 2.77266635

Cl 0.22986139 1.98869686 0.64936339

**TS: Fe(CO)_4_ + CH_3_Cl**

***E*** = -2146.78

**H** = -2093.51

**G** = -2128.57

***N*_imag_** = 1, -495.43 cm^–1^

Fe -0.01429889 -0.11269874 -0.13857699

O -0.06852218 -0.49641017 -3.02548199

C -0.03596406 -0.25567690 -1.89807899

O -0.33404494 -2.82228609 0.79166232

O -2.91481956 0.23119949 0.08394145

O 2.88603516 -0.46463908 0.07135333

C 1.74573416 -0.31735114 0.00222885

C -1.77238968 0.10424892 0.00992306

C -0.20459094 -1.72268538 0.45468050

C 0.14161184 1.15746084 2.12213979

H -0.69828766 1.77643887 2.42327756

H 1.11104070 1.54760304 2.41749445

H 0.01015151 0.11363674 2.37992803

Cl 0.26296998 2.19488517 0.21572376

**P: Fe(CO)_4_ + CH_3_Cl**

***E*** = -2187.36

**H** = -2132.86

**G** = -2167.30

***N*_imag_** = 0

Fe 0.33093818 -0.02881487 0.42087429

O -2.07971223 -0.36596849 -1.20104467

C -1.13011226 -0.24308374 -0.57146966

O -0.55415038 2.34501538 1.79235124

O 1.95097857 1.38156260 -1.56368296

O -0.38352240 -1.88404422 2.56541825

C -0.13022520 -1.15219512 1.72318340

C 1.29477969 0.84115422 -0.79786029

C -0.20994704 1.39624218 1.23977484

C 2.06989184 0.18331183 1.57217398

H 2.56501440 1.13675228 1.37234080

H 2.69916884 -0.65242942 1.26316322

H 1.84822299 0.12773522 2.64077392

Cl 1.18469389 -1.91172218 -0.58711381

**RC: Fe(CO)_4_ + C_2_H_6_**

***E*** = -2589.31

**H** = -2512.40

**G** = -2549.21

***N*_imag_** = 0

Fe -0.04757578 0.12197323 0.39976386

O -2.05325228 -0.66088348 -1.51528489

C -1.18535077 -0.43464220 -0.78187352

O -0.50757653 2.43697079 2.05278637

O 1.13340471 1.87593406 -1.62519506

O -1.41089828 -1.51241064 2.40975412

C -0.85371760 -0.88414754 1.62202266

C 0.68936189 1.17380734 -0.82802158

C -0.22338940 1.49467208 1.44152169

C 4.12323181 -2.01965385 0.77444093

H 4.37797617 -2.91775412 0.19959108

H 4.77527639 -1.99747048 1.65551947

H 4.37806942 -1.15095074 0.15590036

C 2.65555387 -2.01310953 1.17107700

H 2.40526372 -1.11480739 1.74956176

H 2.40111484 -2.88039174 1.79176817

H 2.00501656 -2.04441900 0.28792820

**TS: Fe(CO)_4_ + C_2_H_6_**

***E*** = -2541.25

**H** = -2465.78

**G** = -2499.05

***N*_imag_** = 1, -621.71 cm^–1^

Fe -0.00983488 0.17516124 -0.08517160

O 0.03977797 -2.41684263 -1.37533315

C 0.01829803 -1.35001954 -0.92516537

O 0.04963446 -0.44037329 2.74791143

O -2.93023373 0.06562753 -0.15108061

O 2.90979022 0.22306481 -0.22321677

C 1.75732761 0.21116134 -0.17200543

C -1.77794365 0.11633342 -0.12703553

C 0.02439530 -0.13155813 1.63377413

C -0.06802213 2.44617394 0.15844692

H -0.93305138 2.39110789 0.81850167

H -0.14124653 3.38285705 -0.39280861

H 0.85534800 2.47551268 0.73584725

C -0.06928757 1.74877952 -1.70370198

H 0.80092663 2.35803051 -1.94793869

H -0.99272043 2.27761731 -1.93855186

H -0.03075773 0.85424487 -2.32574463

**P: Fe(CO)_4_ + C_2_H_6_**

***E*** = -2578.97

**H** = -2502.19

**G** = -2536.87

***N*_imag_** = 0

Fe 0.32945709 -0.05469896 0.40141263

O -2.07842475 -0.38647333 -1.19443617

C -1.12452707 -0.25479792 -0.56346469

O -0.57921349 2.34363627 1.77100097

O 2.02402957 1.20595415 -1.59763694

O -0.25456433 -1.99979697 2.48286431

C -0.05513131 -1.21808069 1.66329281

C 1.32854932 0.72875454 -0.81588334

C -0.22171034 1.39346745 1.22810678

C 2.09186231 0.13861601 1.53626919

H 2.57838291 1.09609337 1.33285040

H 2.76410824 -0.67845192 1.26900014

H 1.86838686 0.09567192 2.60540678

C 1.04552014 -1.78562933 -0.55873600

H 2.04297912 -2.00467554 -0.17386477

H 1.09431879 -1.63433301 -1.64021001

H 0.38366888 -2.63432263 -0.36763315

**RC: Fe(CO)_3_PH_3_ + CH_4_**

***E*** = -2213.00

**H** = -2142.08

**G** = -2176.76

***N*_imag_** = 0

Fe -0.05251700 0.11717100 -0.22192500

O -0.08935300 -2.76442200 -0.38179500

C -0.06358100 -1.60067800 -0.40399400

O -2.94194900 0.03113900 -0.10083500

C -1.78738800 0.07957200 -0.17018100

O -0.09171500 1.48284400 2.32458800

C -0.06555200 1.01701200 1.25776200

H 2.93491000 0.75090500 -0.98381200

P 2.12006100 0.02670200 -0.06178300

H 2.76654600 0.47798600 1.11878600

H 2.80723800 -1.21151600 -0.17095200

H -0.89326500 1.73678500 -2.92674200

C -0.00091900 1.59625700 -2.31468700

H 0.90345800 1.80182800 -2.89196800

H -0.04408800 2.26441300 -1.45218200

H 0.03057600 0.49639300 -2.06978400

**TS Fe(CO)_3_PH_3_ + CH_4_**

***E*** = -2200.48

**H** = -2133.09

**G** = -2166.11

***N*_imag_** = 1, -726.26

Fe -0.06286300 0.09215700 -0.26220900

O -0.06458200 -2.79262600 -0.00807000

C -0.05120900 -1.63798200 -0.10806600

O -2.94491100 0.15620900 -0.47080300

C -1.79332400 0.12979300 -0.37908200

O -0.07472500 1.34752500 2.37444800

C -0.05789000 0.86984700 1.31871100

H 2.76804300 -0.29259100 -1.51783000

P 2.10115300 0.11282700 -0.33035600

H 2.80541900 1.33656900 -0.14805800

H 2.86228800 -0.66464600 0.58184900

H -0.88204900 1.79151000 -2.30714600

C 0.02854300 1.66184500 -1.71712200

H 0.87621900 1.67364800 -2.41217500

H 0.12785600 2.51964800 -1.04706000

H -0.03151300 0.12182400 -1.76434300

**P: Fe(CO)_3_PH_3_ + CH_4_**

***E*** = -2210.92

**H** = -2142.56

**G** = -2175.04

***N*_imag_** = 0

Fe -0.21612591 0.44993894 0.31671166

O 1.22441492 -0.46575817 -2.02741602

C 0.65336389 -0.10839309 -1.08860523

O -1.73388240 2.51745579 -1.00801265

C -1.14797520 1.66696308 -0.49418741

O -2.12812843 -1.74554998 0.48330668

C -1.36027642 -0.88698980 0.41076745

H 1.64716639 0.79199675 2.72555313

P 1.25012379 -0.15436903 1.74893959

H 1.00059874 -1.23254180 2.64454350

H 2.55146296 -0.56548470 1.35039107

H -2.19676067 1.38125607 1.86161062

C -1.13330883 1.20432025 2.04572144

H -0.66278584 2.14907687 2.33022853

H -1.07018563 0.50358243 2.88764342

H 0.71205265 1.63789708 0.40251104

**RC: Fe(CO)_3_PH_3_ + CH_3_Cl**

***E*** = -2180.83

**H** = -2115.12

**G** = -2152.02

***N*_imag_** = 0

Fe -0.07120726 0.14749962 -0.20319727

O -0.10296427 -2.73107234 -0.46550029

C -0.07909047 -1.56755988 -0.43440493

O -2.95520938 0.03239403 -0.02314576

C -1.80184445 0.09312711 -0.11911855

O -0.06907498 1.40833760 2.39833775

C -0.06060033 0.96965198 1.31874597

H 2.92262145 0.70338367 -1.01633923

P 2.10151154 0.03616102 -0.05677595

H 2.77344482 0.51509447 1.09992842

H 2.76312182 -1.21952319 -0.11922451

Cl 0.01234295 1.35475730 -2.21406290

C -0.36692961 3.08763714 -2.00796648

H 0.34958724 3.51293988 -1.30396233

H -1.38506225 3.16808571 -1.62489895

H -0.27968894 3.55458590 -2.99167778

**TS: Fe(CO)_3_PH_3_ + CH_3_Cl**

***E*** = -2148.63

**H** = -2084.40

**G** = -2119.87

***N*_imag_** = 1, -494.99

Fe -0.09499386 0.27226498 -0.23990431

O -0.09224465 -2.62612722 -0.09031993

C -0.05646305 -1.47884305 -0.25695506

O -2.99290788 0.35074117 -0.22605230

C -1.83646670 0.33328531 -0.24320645

O -0.10964484 1.02264409 2.54157120

C -0.09515577 0.76936430 1.40646287

H 2.78520597 1.59601030 -0.33652490

P 2.09087664 0.35456299 -0.24021338

H 2.80683802 -0.16515799 0.86922255

H 2.79275736 -0.31909707 -1.27467579

Cl 0.05631838 0.95536118 -2.50375570

C -0.24882452 2.68817867 -1.22472366

H 0.10545993 2.84915605 -0.21247494

H -1.32393275 2.79580219 -1.33144334

H 0.31893819 3.25424171 -1.9568793

**P: Fe(CO)_3_PH_3_ + CH_3_Cl**

***E*** = -2195.41

**H** = -2129.86

**G** = -2164.28

***N*_imag_** = 0

Fe -0.07620264 0.09298671 -0.20675585

O -0.06554259 -2.76932881 0.34763379

C -0.05622334 -1.64455019 0.10579738

O -2.93933132 0.36459563 -0.60869467

C -1.81004439 0.24290093 -0.44120015

O -0.13085169 0.89491236 2.55067642

C -0.10805002 0.56215298 1.44546829

H 2.50859400 1.22595322 -1.42392437

P 2.07939019 0.25677064 -0.49121227

H 2.93079916 0.61317971 0.59840270

H 2.79343489 -0.87542076 -0.94859303

Cl 0.20413352 -0.33597910 -2.46013861

C -0.05446317 2.14966678 -0.56015293

H 0.78695842 2.65201040 -0.06940044

H -0.97016840 2.64100944 -0.21990322

H 0.03167276 2.23795981 -1.64603631

**RC: Fe(CO)_3_PH_3_ + C_2_H_6_**

***E*** = -2591.26

**H** = -2504.15

**G** = -2541.30

***N*_imag_** = 0

Fe -0.11873191 -0.00519340 -0.10958882

O -0.06010021 -2.88419028 -0.05748580

C -0.08208091 -1.72794825 -0.19250978

O -3.00661941 -0.08633931 0.05701438

C -1.85225373 -0.04927594 -0.01940344

O -0.05173014 1.49621186 2.34711638

C -0.07679078 0.98978840 1.29897706

H 2.51608139 0.77955520 -1.48667821

P 2.05525284 0.10832411 -0.31487955

H 2.86865020 0.79874713 0.62283467

H 2.86945346 -1.04953943 -0.43555873

C 0.55563548 1.71430470 -5.08737423

C -0.55706610 1.61827405 -4.05621241

H 1.53971861 1.51813309 -4.64374648

H 0.59693439 2.71122916 -5.54211537

H 0.41465016 0.99009286 -5.89847335

H -0.60343206 0.61895323 -3.60584735

H -1.53809371 1.81441064 -4.50450746

H -0.42115844 2.34561294 -3.24633384

**TS: Fe(CO)_3_PH_3_ + C_2_H_6_**

***E*** = -2541.09

**H** = -2454.20

**G** = -2488.96

***N*_imag_** = 1, -646.93cm^–1^

Fe -0.14240531 0.30371010 -0.22861898

O -0.02098689 -2.59145994 -0.22778153

C -0.04136126 -1.43211958 -0.31412942

O -3.03294423 0.30537321 -0.06098902

C -1.87713102 0.30589968 -0.14012873

O -0.11841121 0.97929818 2.58191540

C -0.11711730 0.78662731 1.43369510

H 2.72193537 1.59829929 0.14406272

P 2.03405235 0.39617722 -0.20372142

H 2.74516649 -0.46830498 0.66646468

H 2.78470891 0.12593703 -1.38558711

H -0.71570650 1.36599142 -3.18316265

C -0.11986564 2.26470215 -1.36917348

H 0.19724493 2.62821273 -0.38945944

H -1.10225132 2.68595645 -1.57995425

H 0.60787012 2.63842221 -2.09380308

C -0.25659358 0.66870804 -2.48042579

H -0.87929028 -0.22541288 -2.49987845

H 0.74733200 0.42891649 -2.83415192

**P: Fe(CO)_3_PH_3_ + C_2_H_6_**

***E*** = -2580.85

**H** = -2493.13

**G** = -2527.77

***N*_imag_** = 0

Fe -0.05726417 0.09252198 -0.18269522

O -0.07383496 -2.76346579 0.32579614

C -0.05844025 -1.62613196 0.12267317

O -2.87193155 0.41945028 -0.75204988

C -1.75366959 0.27804491 -0.50753523

O -0.11886047 0.86176140 2.61372924

C -0.08464562 0.55634408 1.49993401

H 2.46818161 1.03831832 -1.63514453

P 2.05470425 0.31099504 -0.49037338

H 2.87355404 0.97973686 0.46196462

H 2.89368118 -0.82313527 -0.67637109

C 0.04082866 -0.40514898 -2.21836921

C 0.01081317 2.14611990 -0.60648552

H 0.87248861 2.64669821 -0.14548674

H -0.87981770 2.65456251 -0.22502680

H 0.05745233 2.28345354 -1.69060034

H 0.07937616 0.51561370 -2.80748177

H -0.83767476 -0.98179025 -2.52330676

H 0.91491130 -1.02290883 -2.46313661

**RC: Fe(CO)_3_BF + CH_4_**

***E*** = -2099.43

**H** = -2041.90

**G** = -2076.71

***N*_imag_** = 0

Fe 0.06549900 -0.03643300 0.02234400

O -1.26197500 -2.61590000 -0.04780500

C -0.64239100 -1.63881600 -0.01525700

O -0.20805600 0.47240600 -2.83532900

F -1.80093500 2.33736800 -0.04248200

O -0.43067900 0.45049300 2.85417200

C -0.19137500 0.24095400 1.73920600

B -0.93121400 1.38319400 -0.01127200

C -0.05535600 0.25514700 -1.70669700

C 2.56320600 -0.31280900 0.12561500

H 2.14498700 -1.32002500 0.10705500

H 3.12281000 -0.16979400 1.05113400

H 3.19813000 -0.16779600 -0.74959800

H 1.81866200 0.53864700 0.09546400

**TS: Fe(CO)_3_BF + CH_4_**

***E*** = -2085.88

**H** = -2030.39

**G** = -2063.60

***N*_imag_** = 1, -737.98 cm^–1^

Fe 0.02908800 0.00063100 0.03023800

O -1.23930000 -2.63651000 -0.02064200

C -0.74558700 -1.59536300 -0.00132800

O 0.33664500 0.72090100 -2.76759100

F -2.28371200 1.98112200 -0.07397800

O 0.09120800 0.73018300 2.84177900

C 0.05412500 0.40806100 1.73198700

B -1.31372400 1.13900500 -0.03088600

C 0.20388200 0.40186300 -1.66422800

C 1.96409500 -0.89942200 0.12297100

H 2.03415200 -1.51169800 -0.77606600

H 1.93787400 -1.52655900 1.01419100

H 2.85697500 -0.26775000 0.17707200

H 1.35093200 0.66813400 0.08728300

**P: Fe(CO)_3_BF + CH_4_**

***E*** = -2091.73

**H** = -2035.53

**G** = -2068.70

***N*_imag_** = 0

Fe -0.02964000 0.10472400 0.02719400

O -1.36077300 -2.49132300 -0.02934400

C -0.83377000 -1.46662500 -0.00637700

O 0.70962500 0.53614100 -2.74300600

F -2.55847100 1.82154800 -0.07954900

O 0.45880000 0.55259700 2.84967000

C 0.23210800 0.34334800 1.73925000

B -1.51051700 1.08260800 -0.03744500

C 0.38414600 0.33405200 -1.65610800

C 1.84066900 -0.86597300 0.11475200

H 1.96649100 -1.49936200 -0.76582500

H 1.88584800 -1.49522200 1.00606900

H 2.63272400 -0.11703100 0.14907400

H 0.71194700 1.41390500 0.05623300

**RC: Fe(CO)_3_BF + CH_3_Cl**

***E*** = -2069.59

**H** = -2015.74

**G** = -2052.57

***N*_imag_** = 0

Fe 0.09251998 0.03763873 -0.01375150

O -1.00498987 -2.64709280 -0.00528997

C -0.48370052 -1.61216350 -0.00767669

O -0.30260895 0.53041969 -2.86051223

F -1.99170384 2.22893757 -0.05676147

O -0.36355500 0.56535123 2.81757528

C -0.13563595 0.34319072 1.70139591

B -1.04855217 1.34570465 -0.03740257

C -0.09838495 0.32233364 -1.73687103

C 3.21906281 -1.41379573 -0.05504687

Cl 2.42983679 0.18488457 0.01554863

H 4.29655409 -1.23530630 -0.03702130

H 2.91754421 -1.90186695 -0.98252997

H 2.90150772 -1.98915545 0.81527711

**TS: Fe(CO)_3_BF + CH_3_Cl**

***E*** = -2031.86

**H** = -1979.49

**G** = -2015.30

***N*_imag_** = 1, -446.45 cm^–1^

Fe 0.20591801 -0.04358523 -0.01840490

O -1.32788885 -2.50028929 -0.01164738

C -0.66896562 -1.55014509 -0.01345745

O 0.02541022 0.32639616 -2.91139414

F -2.02819283 2.00794441 -0.04245253

O -0.01319631 0.35206322 2.86825735

C 0.09724863 0.18695036 1.72995956

B -1.00414994 1.22657046 -0.03235186

C 0.12113473 0.17115832 -1.77035194

C 2.25315362 -1.53616168 -0.01071949

Cl 2.41894213 0.72984798 -0.00005500

H 3.32937487 -1.42469995 -0.04660261

H 1.85008361 -2.00289176 -0.90408641

H 1.90173398 -1.96016579 0.92482593

**P: Fe(CO)_3_BF + CH_3_Cl**

***E*** = -2084.47

**H** = -2004.60

**G** = -2038.13

***N*_imag_** = 0

Fe 0.18785297 -0.01564250 0.07591665

O -1.39443034 -2.39938103 0.01356918

C -0.75561775 -1.44095387 0.02513388

O -0.18539573 0.06072805 -2.81913274

F -2.19924632 1.93810047 -0.14262153

O -0.57731997 0.09423924 2.90088993

C -0.20785681 0.04207440 1.80996412

B -1.06688178 1.26748389 -0.03690716

C 0.04227527 0.04201794 -1.69179990

H 2.99251014 -0.33575287 -0.22878902

Cl 1.10122996 2.05388212 -0.20905756

H 2.03639140 -1.39567675 0.89493701

H 1.88290166 -1.58665748 -0.90452613

C 2.08704795 -0.91548635 -0.07752415

**RC: Fe(CO)_3_BF + C_2_H_6_**

***E*** = -2479.27

**H** = -2402.95

**G** = -2439.92

***N*_imag_** = 0

Fe 0.07621809 0.01320636 -0.01963495

O -1.13738728 -2.61745552 -0.18290347

C -0.56210542 -1.61521434 -0.11709754

O -0.31325491 0.67582941 -2.83148580

F -1.89588524 2.29587226 0.11658604

O -0.31003354 0.37757183 2.84740048

C -0.11183281 0.21554091 1.71586779

B -0.98588945 1.38087682 0.06020710

C -0.11562896 0.39966270 -1.72250145

C 3.71618165 0.33934803 0.87328764

C 2.65103981 -0.25994182 -0.02929842

H 4.04639114 1.31969129 0.51284981

H 4.59444420 -0.31653387 0.90612647

H 3.35669800 0.46233972 1.89990523

H 2.99744979 -0.38905737 -1.05814083

H 1.81813616 0.51411374 -0.09890234

H 2.30871035 -1.22967593 0.33631497

**TS: Fe(CO)_3_BF + C_2_H_6_**

***E*** = -2428.29

**H** = -2353.19

**G** = -2387.88

***N*_imag_** = 1**,** -592.59 cm^–1^

Fe 0.21750098 -0.00992160 -0.00414602

O -1.20692080 -2.54301597 -0.01028247

C -0.58050584 -1.57122836 -0.00655275

O 0.03808236 0.48531251 -2.87552462

F -2.12917849 1.90923262 -0.05305428

O -0.05913148 0.53334763 2.85060689

C 0.07948983 0.31301569 1.72018872

B -1.07679794 1.16088858 -0.03269748

C 0.13812957 0.28435730 -1.73754936

C 2.21368739 -1.08864960 0.04041116

H 2.85763261 0.92943615 0.93035762

H 3.28616614 -0.90464607 0.06348690

H 1.99406187 -1.65815135 -0.86189044

H 1.95467823 -1.64774910 0.93877652

C 2.25678463 0.92161764 0.02091603

H 1.67254284 1.84223481 -0.00148308

H 2.89195668 0.90795924 -0.86488429

**P: Fe(CO)_3_BF + C_2_H_6_**

***E*** = -2464.65

**H** = -2378.37

**G** = -2411.13

***N*_imag_** = 0

Fe -0.06204425 0.03961473 0.06340802

O -1.37793643 -2.49520331 -0.04302050

C -0.88769417 -1.45422919 -0.00047504

O 0.30965272 -0.31444168 -2.78592081

F -2.51090249 1.87365257 -0.16102357

O 0.02296132 -0.33528858 2.96029345

C -0.02796039 -0.14522947 1.81428062

B -1.36078108 1.26307182 -0.03373135

C 0.13113290 -0.13556976 -1.67204473

C 1.88205676 -0.79293364 0.08213833

C 0.73952934 2.00208231 0.08791886

H 1.64429451 1.46795089 0.36635684

H 0.56761844 2.79224491 0.83095526

H 0.89286901 2.49986694 -0.87478702

H 2.56948791 -0.14194889 -0.47310073

H 1.89025197 -1.77793797 -0.38995628

H 2.25375383 -0.89919321 1.10359256

**RC: Fe(CO)_3_BN(CH_3_)_2_+CH_4_**

***E*** = -3101.50

**H** = -2993.09

**G** = -3032.28

***N*_imag_** = 0

Fe 0.56668400 -0.03356000 0.33965600

C 0.64663100 -1.43973600 -0.64964400

B -1.22647900 -0.18966600 0.09530500

O 0.67263800 -2.28816300 -1.45061400

O 3.43371900 0.58825100 0.14430700

O -0.09554400 2.39064400 -1.05680200

C 0.15469400 1.44783900 -0.41450600

C 2.31455600 0.32879900 0.27618100

N -2.56882000 -0.26497800 -0.18908500

H -2.38197200 -2.12828300 -1.13380900

H -4.26506800 0.40281900 0.86114500

C -3.48457500 0.79348700 0.19433100

H -2.94577200 1.58660600 0.71827200

H -3.96494400 1.22812600 -0.69231500

C -3.15282800 -1.39492500 -0.88809600

H -3.91614700 -1.88219900 -0.26685900

H -3.62294100 -1.06158000 -1.82311100

C 0.62318100 -0.53780000 2.82709100

H 0.94209400 -1.44169400 2.30358900

H -0.25732700 -0.76321500 3.43089700

H 1.44020700 -0.16396000 3.44574500

H 0.31525700 0.32119800 2.16134600

**TS: Fe(CO)_3_BN(CH_3_)_2_ + CH_4_**

***E*** = -3094.71

**H** = -2987.62

**G** = -3025.85

***N*_imag_** = 1, -809.78 cm^–1^

Fe 0.55488668 -0.17613750 0.10867845

C 1.10370511 -1.55500085 -0.83111507

B -1.23242508 -0.02827549 0.16866933

O 1.41252067 -2.46988260 -1.46981403

O 2.82582497 1.41361524 1.05456682

O -0.04203375 1.65017960 -2.04236031

C 0.19807364 0.91778826 -1.17211670

C 1.94864080 0.75397339 0.68877076

N -2.59757732 0.14895199 0.21411102

H -2.94544911 -1.54167477 -0.97125530

H -3.89783757 0.89273658 1.69818986

C -3.20840061 1.25883236 0.92485029

H -2.43625473 1.86433174 1.40605020

H -3.77124758 1.90091122 0.23334601

C -3.50893107 -0.74688799 -0.47617055

H -4.21275183 -1.20616778 0.23161144

H -4.08617554 -0.20353736 -1.23689569

C 0.61895125 -1.39867620 1.91633720

H -0.24253272 -0.25931212 1.40674609

H 0.46366814 -0.92101334 2.89082800

H 1.65881091 -1.71795076 1.86795117

H -0.04675484 -2.25707260 1.82260035

**P: Fe(CO)_3_BN(CH_3_)_2_ + CH_4_**

***E*** = -3108.31

**H** = -2999.65

**G** = -3037.74

***N*_imag_** = 0

Fe 0.30824422 0.25522676 1.88982757

C -0.62955145 1.68971152 2.01611453

B 0.46589175 -0.01474067 0.10815549

O -1.24738697 2.66851404 2.05051926

O 1.51050321 -0.13073000 4.53836417

O -1.98444908 -1.50078410 2.15983846

C -1.05455940 -0.82055542 2.05637120

C 1.02457304 0.01703718 3.50230592

N 0.54227793 -0.14057923 -1.26266522

H -0.95326917 1.29967070 -1.53644507

H 2.14442631 -0.50109473 -2.58489266

C 1.45640638 -1.05031903 -1.92711952

H 2.05022515 -1.59630521 -1.19021084

H 0.90601016 -1.78098809 -2.53589987

C -0.30832700 0.65067082 -2.13349811

H 0.29492645 1.28026156 -2.80226622

H -0.94559655 0.00126781 -2.74959293

C 1.87343875 1.60797465 1.53268013

H 1.18380225 -0.78014005 0.98100444

H 2.80786016 1.03960459 1.52274365

H 1.91752953 2.33647747 2.34679513

H 1.77362316 2.15689063 0.59199855

**RC: Fe(CO)_3_BN(CH_3_)_2_ + CH_3_Cl**

***E*** = -3070.41

**H** = -2965.75

**G** = -3006.68

***N*_imag_** = 0

Fe 0.38722200 0.08484800 0.34956600

C 0.39385500 -1.24257800 -0.72728800

B -1.35375300 -0.24207200 -0.04789100

O 0.43198100 -2.06825800 -1.55632700

O 3.32217200 0.10929100 0.52952500

O 0.13266600 2.58147300 -1.06928900

C 0.23893300 1.61524800 -0.42723900

C 2.16432900 0.10966000 0.50852100

N -2.65584900 -0.49500100 -0.41131500

H -2.41642400 -2.57452300 -0.31645800

H -4.44042300 0.54172200 0.00619800

C -3.58287700 0.58960400 -0.67861400

H -3.08339200 1.55166700 -0.54290100

H -3.95481400 0.53363300 -1.71051400

C -3.18901800 -1.83698200 -0.54428500

H -4.03302900 -1.98787900 0.14250600

H -3.53900400 -2.01146600 -1.57069900

C 0.34442600 -1.29202400 3.49742000

Cl -0.02690700 0.23737200 2.65838000

H 0.09867900 -1.15160800 4.55267300

H 1.40649200 -1.50389300 3.36581600

H -0.26578700 -2.07940500 3.05214700

**TS: Fe(CO)_3_BN(CH_3_)_2_ + CH_3_Cl**

***E*** = -3038.39

**H** = -2934.17

**G** = -2975.14

***N*_imag_** = 1, -496.43 cm^–1^

Fe 0.42595041 -0.00315374 0.30439623

C 0.42801287 -1.14943040 -0.96330196

B -1.33051782 -0.34487840 -0.04204490

O 0.45864710 -1.86787920 -1.88319199

O 3.35002415 0.08063685 0.62190466

O 0.22454527 2.47978521 -1.16287796

C 0.29626506 1.52557514 -0.50654834

C 2.19731532 0.04095563 0.54832710

N -2.64955786 -0.57127444 -0.34486228

H -2.41063159 -2.65580880 -0.37098959

H -4.40346086 0.44152789 0.23472895

C -3.59140708 0.52638100 -0.49969695

H -3.08353271 1.48082730 -0.34426759

H -4.02673805 0.52165104 -1.50797863

C -3.19544148 -1.90818079 -0.50565882

H -3.98787504 -2.09552834 0.23149819

H -3.61774788 -2.03073868 -1.51208872

C 0.42180884 -1.60641671 2.32090837

Cl -0.10667674 0.47522116 2.55727902

H 1.19651368 -1.50662859 3.07549019

H 0.74582253 -2.19503347 1.47043256

H -0.53766078 -1.92906387 2.71554368

**P: Fe(CO)_3_BN(CH_3_)_2_ + CH_3_Cl**

***E*** = -3100.52

**H** = -2995.59

**G** = -3035.23

***N*_imag_** = 0

Fe 0.56211800 -0.03605900 0.30397800

C 0.62154700 -1.22588800 -0.89605900

B -1.34736400 0.01754100 0.47544500

O 0.65902300 -2.04298200 -1.69995200

O 3.42090500 0.18825900 1.00535400

O 0.47750500 2.12917300 -1.64882000

C 0.51183900 1.30468100 -0.82614700

C 2.29289800 0.11985600 0.75337400

N -2.65863200 -0.27018400 0.13172200

H -2.06190900 -1.25723300 -1.62065200

H -4.39871400 -0.77789600 1.19634700

C -3.82935200 0.11252200 0.89589300

H -3.54221300 0.65843400 1.79521000

H -4.49108900 0.75114100 0.29477900

C -2.97206200 -1.01846400 -1.07173000

H -3.48690600 -1.95667200 -0.82249600

H -3.63007300 -0.43142800 -1.72684600

C 0.51705000 -1.73731400 1.51013300

Cl -0.85149600 1.01258600 1.92249900

H 0.67396700 -1.34638000 2.52044800

H 1.31409900 -2.44947800 1.27979300

H -0.43970500 -2.26542500 1.46971200

**RC: Fe(CO)_3_BN(CH_3_)_2_ + C_2_H_6_**

***E*** = -3480.16

**H** = -3350.67

**G** = -3391.39

***N*_imag_** = 0

Fe 1.08591200 0.25739000 -0.81252000

C 0.47711400 -0.96368100 -1.84981300

B -0.46899900 0.03153000 0.10571400

O -0.03975600 -1.65542800 -2.63418900

O 3.46999800 0.90408400 -2.42511300

O -0.15467000 2.81406300 -1.18631600

C 0.39601700 1.81191700 -0.94396600

C 2.56005600 0.63655200 -1.76506000

N -1.68107900 -0.10359800 0.73585100

H -1.84956300 -2.11292000 0.16104900

H -2.45565000 0.71523400 2.51242900

C -2.30522500 0.98907600 1.46015900

H -1.67493500 1.87967000 1.41614500

H -3.28058000 1.23054900 1.01760500

C -2.40475500 -1.36275300 0.72854000

H -2.54630400 -1.73034900 1.75342300

H -3.39043500 -1.23259300 0.26273600

C 2.85094247 -1.42324371 1.57110531

C 2.73371426 0.00191399 2.08548401

H 2.79875325 -2.14938302 2.39037354

H 3.80249967 -1.58762088 1.05172581

H 2.04471401 -1.66681228 0.86898193

H 3.53774671 0.24134780 2.79079328

H 1.78303141 0.16299007 2.60675596

H 2.78863783 0.73218019 1.26968429

**TS: Fe(CO)_3_BN(CH_3_)_2_ + C_2_H_6_**

***E*** = -3431.65

**H** = -3304.82

**G** = -3345.33

***N*_imag_** = 1, -639.86cm^–1^

Fe 0.55294226 -0.01636317 0.22546650

C 0.31526440 -0.96450149 -1.19060961

B -1.26524485 -0.01055689 0.13075886

O 0.18284414 -1.51360364 -2.21065761

O 3.48432764 -0.04674062 0.12041218

O 0.24634590 2.65540468 -0.82019429

C 0.36644392 1.60419087 -0.33269203

C 2.32790558 -0.04721857 0.18572592

N -2.63442130 -0.00649126 0.00144243

H -2.69131161 -1.98393579 -0.69670004

H -4.15216376 0.92517220 1.13112720

C -3.42919189 1.16294113 0.33873672

H -2.78169216 1.96936008 0.69039178

H -3.98059033 1.52196428 -0.54082942

C -3.37536385 -1.16139765 -0.47743129

H -4.09742551 -1.49906089 0.27872255

H -3.92342405 -0.91412875 -1.39675984

C 0.47162230 -1.59400250 1.84795426

C 0.62947774 0.26365149 2.47177825

H 1.14709530 -1.81228955 2.67681847

H 0.77484205 -2.24303724 1.02619022

H -0.55102224 -1.83140149 2.13994343

H 1.64807311 0.23612902 2.85580563

H -0.07220692 -0.03320811 3.25339972

H 0.38340937 1.28944354 2.20026628

**P: Fe(CO)_3_BN(CH_3_)_2_ + C_2_H_6_**

***E*** = -3496.95

**H** = -3304.29

**G** = -3341.52

***N*_imag_** = 0

Fe 0.55294226 -0.01636317 0.22546650

C 0.29850954 -0.86260295 -1.25097206

B -1.17302957 0.11386608 0.79015154

O 0.15584429 -1.33914810 -2.30553242

O 3.45870034 0.04916595 0.62202085

O 0.21414132 2.57326496 -1.00123286

C 0.34663295 1.55956016 -0.44268026

C 2.30871823 0.01084657 0.48916430

N -2.49359902 0.21263948 1.16126280

H -2.93410639 -1.74397845 0.54601829

H -3.43635922 1.22483935 2.75323682

C -3.03130844 1.42701740 1.75215592

H -2.24653637 2.18161581 1.84127802

H -3.83533797 1.83773504 1.12651865

C -3.43514914 -0.88106980 0.98977505

H -3.85927831 -1.18232873 1.95751243

H -4.25808405 -0.58136662 0.32672677

C 0.43029356 -1.90721410 1.14234438

C 0.67222920 0.94048101 2.09662786

H 1.08183628 -2.40303381 1.86385931

H 0.75360958 -2.24201143 0.15651380

H -0.60089627 -2.21652674 1.31084076

H 1.70026880 1.04939805 2.43874290

H -0.00603468 0.92533905 2.95169975

H 0.40626466 1.81125507 1.49868713

**RC: Fe(CO)_4_ + CH_4_(90°)**

***E*** = -2207.48

**H** = -2149.27

**G** = -2183.45

***N*_imag_** = 0

Fe 0.32306800 0.07538900 0.50055300

O -2.18707500 0.11646500 -0.86656700

C -1.17189800 0.09953500 -0.31416900

O -0.54116800 2.47057000 1.95045800

O 1.56471800 0.44107300 -2.13004400

O -0.48961400 -2.54145800 1.54397800

C -0.13023400 -1.51560200 1.14996900

C 1.11564700 0.29012700 -1.07541500

C -0.14864600 1.52725200 1.40914900

C 2.62067700 -0.24419700 1.53427800

H 2.71504900 -0.62083300 2.55436700

H 2.58047000 -1.07592300 0.83433500

H 1.72442100 0.45203500 1.59225200

H 3.44244100 0.42401600 1.27037300

**TS: Fe(CO)_4_ + CH_4_(90°)**

***E*** = -2197.46

**H** = -2141.02

**G** = -2173.40

***N*_imag_** = 1, -861.77cm^–1^

Fe 0.31055481 0.08379419 0.49486009

O -2.11890350 -0.17181135 -1.06573643

C -1.13850483 -0.09893528 -0.46280384

O -0.86372811 2.45601440 1.75671918

O 1.77119044 0.93924685 -1.87285899

O -0.43811878 -2.25379422 2.05899750

C -0.16239688 -1.30830049 1.45892350

C 1.18038583 0.63205020 -0.93124236

C -0.38562221 1.52693488 1.27290901

C 2.12148480 0.22508524 1.63150420

H 2.92825500 -0.50808576 1.50380195

H 1.79378323 0.18242316 2.67000991

H 2.52217658 1.20868594 1.38744258

H 1.55100674 -0.75168171 0.51339049

**P: Fe(CO)_4_ + CH_4_(90°)**

***E*** = -2208.19

**H** = -2151.01

**G** = -2183.14

***N*_imag_** = 0

Fe 0.23769300 0.02872300 0.41048500

O -2.24622200 -0.11093900 -1.09976300

C -1.25574200 -0.07604500 -0.51425300

O -0.80328300 2.41120600 1.75944300

O 1.95470800 1.03688200 -1.70437000

O -0.25555500 -2.16543400 2.24846200

C -0.09912700 -1.26811600 1.54597500

C 1.25074300 0.68524000 -0.86543100

C -0.37749600 1.47767100 1.23984500

C 2.01167700 0.17599200 1.53038500

H 2.67831400 -0.64341300 1.25764700

H 1.77985600 0.12492100 2.59632400

H 2.49827700 1.13198700 1.32605800

H 0.81765400 -1.19048500 -0.24885200

**RC: Fe(CO)_4_ + CH_4_(95°)**

***E*** = -2207.18

**H** = -2148.96

**G** = -2183.34

***N*_imag_** = 0

Fe 0.31629300 0.13166300 0.54442300

O -2.04114700 -0.18818200 -1.04422000

C -1.09119800 -0.05227000 -0.39936200

O -0.67918600 2.55004200 1.87970900

O 1.69948800 0.54919200 -2.00285000

O -0.43175700 -2.42932900 1.75170400

C -0.11237100 -1.41545900 1.29620700

C 1.18239200 0.38991100 -0.98081400

C -0.24802800 1.60213000 1.38010000

C 2.42087900 0.32192900 1.90804000

H 2.67640300 1.32507100 1.57661700

H 3.33410800 -0.27719000 2.00693200

H 1.88470700 0.31930500 2.85335900

H 1.94034400 -0.32083300 1.08887000

**TS: Fe(CO)_4_ + CH_4_(95°)**

***E*** = -2198.54

**H** = -2141.25

**G** = -2173.74

***N*_imag_** = 1, -851.60 cm^–1^

Fe 0.31385466 0.07194864 0.49045653

O -2.03864796 -0.32945944 -1.15317891

C -1.09136230 -0.18991894 -0.50983935

O -0.76379399 2.47451074 1.78024536

O 1.76969890 0.98709714 -1.85821321

O -0.49376086 -2.17979295 2.14881296

C -0.19605530 -1.26764255 1.50965545

C 1.17981544 0.65633232 -0.92450976

C -0.32864244 1.53252730 1.28139225

C 2.15658638 0.14056710 1.59009332

H 2.58369817 1.11266330 1.34472823

H 2.93774458 -0.61632234 1.44325172

H 1.84906229 0.10321766 2.63494630

H 1.53082574 -0.79957062 0.48994360

**P: Fe(CO)_4_ + CH_4_(95°)**

***E*** = -2208.74

**H** = -2151.52

**G** = -2183.65

***N*_imag_** = 0

Fe 0.23058900 0.02668300 0.40818100

O -2.17895200 -0.28062800 -1.19379000

C -1.22462900 -0.16005700 -0.56156600

O -0.68316500 2.43972500 1.79308900

O 1.95464200 1.03282500 -1.70233700

O -0.29562600 -2.12448200 2.28785800

C -0.12725200 -1.24394200 1.56737100

C 1.24692900 0.68105400 -0.86679900

C -0.32902700 1.49168100 1.24694300

C 2.00478400 0.17114400 1.53402700

H 2.50247900 1.11983700 1.32242600

H 2.66276900 -0.65759600 1.26913100

H 1.77224600 0.13168400 2.60034000

H 0.82854000 -1.19899700 -0.21988900

**RC: Fe(CO)_4_ + CH_4_(100°)**

***E*** = -2206.76

**H** = -2148.54

**G** = -2182.65

***N*_imag_** = 0

Fe 0.41553100 0.02275200 0.50736300

O -1.82100900 -0.54364700 -1.20249900

C -0.86175800 -0.41942200 -0.56484000

O -0.95185300 2.32435500 1.57717000

O 1.60265600 1.61211200 -1.65128700

O -0.75442200 -1.73725200 2.53868300

C -0.28494600 -1.04696900 1.74285400

C 1.14552200 0.98407200 -0.79875600

C -0.28437000 1.43801800 1.24434500

C 2.68583100 -0.79502800 1.13100300

H 3.55594500 -0.62930800 0.49426500

H 2.83016600 -1.65232200 1.78992300

H 2.49991900 0.09807500 1.73186200

H 1.86224700 -1.11764000 0.41429200

**TS: Fe(CO)_4_ + CH_4_(100°)**

***E*** = -2198.84

**H** = -2142.34

**G** = -2175.55

***N*_imag_** = 1, -798.16 cm^–1^

Fe 0.33861495 0.03314767 0.47328359

O -1.92511466 -0.51864037 -1.24853165

C -1.01222141 -0.31910877 -0.57174484

O -0.73136247 2.42485063 1.78336299

O 1.72020526 1.34533813 -1.73129410

O -0.59478506 -1.95055863 2.39272037

C -0.24572010 -1.15015035 1.64212252

C 1.15981475 0.84789391 -0.85699406

C -0.27495904 1.48815964 1.29126656

C 2.29371453 -0.27482989 1.33131921

H 3.13366245 -0.17484220 0.63986601

H 2.41760293 -1.19508709 1.90711466

H 2.29980114 0.57286372 2.01761749

H 1.43661866 -0.98038223 0.28174015

**P: Fe(CO)_4_ + CH_4_(100°)**

***E*** = -2208.66

**H** = -2137.72

**G** = -2185.85

***N*_imag_** = 0

Fe 0.31130900 0.02290400 0.44670000

O -2.01291100 -0.45373700 -1.23723400

C -1.10198800 -0.24761400 -0.56428000

O -0.51281300 2.41576100 1.91939500

O 2.01758100 1.09437600 -1.64657600

O -0.21550500 -2.12967200 2.32513700

C -0.04742100 -1.24956200 1.60430700

C 1.31537900 0.71596400 -0.81825300

C -0.21304300 1.47602900 1.32813800

C 2.08664100 0.16998300 1.57475700

H 2.74838600 -0.65213700 1.29942000

H 1.85706100 0.11742100 2.64117400

H 2.57921400 1.12350500 1.37303100

H 0.94241200 -1.18648800 -0.17690300

**RC: Fe(CO)_4_ + CH_3_Cl(90°)**

***E*** = -2179.52

**H** = -2139.64

**G** = -2185.27

***N*_imag_** = 0

Fe -0.02331900 -0.15207600 -0.18824100

O -0.02759000 -0.07002200 -3.11603500

C -0.01919500 -0.05403200 -1.95967800

O -0.34170300 -2.99508200 -0.34272200

O -2.61306300 -0.11038200 1.18191700

O 2.51214500 -0.67767100 1.18336900

C 1.51766800 -0.43109200 0.64349100

C -1.58759900 -0.08649200 0.64420800

C -0.21260800 -1.84758500 -0.28252300

C 0.30608800 2.75094800 1.60326900

H 0.44159800 3.83364700 1.55725900

H 1.15257500 2.26819300 2.09266000

H -0.63446500 2.49171800 2.09039600

Cl 0.23790000 2.18390700 -0.08303100

**TS: Fe(CO)_4_ + CH_3_Cl (90°)**

***E*** = -2144.87

**H** = -2091.45

**G** = -2126.40

***N*_imag_** = 1, -482.63 cm^–1^

Fe -0.01725423 -0.14202730 -0.14397631

O -0.10284152 -0.75649522 -3.00066328

C -0.05479385 -0.40260436 -1.90481036

O -0.35314052 -2.97451838 0.19931616

O -2.88227077 0.17998341 0.39909703

O 2.84534511 -0.50385604 0.38734512

C 1.71761296 -0.34476351 0.20315488

C -1.74942248 0.06911182 0.21099086

C -0.21741023 -1.82967984 0.11003901

C 0.16279057 1.33871799 2.03404751

H -0.68280800 1.97279472 2.28466996

H 1.13110389 1.76308949 2.28335168

H 0.04470637 0.31502678 2.36918338

Cl 0.25946904 2.17125168 0.02883973

**P: Fe(CO)_4_ + CH_3_Cl(90°)**

***E*** = -2186.70

**H** = -2132.49

**G** = -2167.06

***N*_imag_** = 0

Fe -0.00928900 -0.06876000 0.20232900

O -0.05984500 -0.42602200 -2.70436600

C -0.03576200 -0.24762700 -1.57201100

O -0.35712900 -2.91944900 0.46699800

O -2.82477900 0.64670700 0.57543400

O 2.89722500 -0.06624200 0.56247400

C 1.76411000 -0.08305200 0.40390500

C -1.73138100 0.35134200 0.41232600

C -0.21889000 -1.78041400 0.37800400

C 0.01473000 0.07390300 2.29425800

H -0.92380100 -0.28535400 2.72222900

H 0.15179200 1.13614400 2.49978600

H 0.83375000 -0.51148200 2.71798100

Cl 0.27005900 2.20149200 -0.00733000

**RC: Fe(CO)_4_ + CH_3_Cl(95°)**

***E*** = -2179.24

**H** = -2124.35

**G** = -2152.25

***N*_imag_** = 0

O 0.00611400 0.17486400 -3.03725000

C 0.00146000 0.07534100 -1.88571600

O -0.35419600 -3.03294500 -0.29126700

O -2.66087100 -0.18862600 1.14164400

O 2.55233500 -0.78920600 1.13373700

C 1.54076100 -0.52413400 0.63568400

C -1.61609400 -0.15869600 0.64254200

C -0.21955400 -1.88539700 -0.23757600

C 0.32057400 2.52594900 1.93456600

H 0.44028300 3.61010800 1.99132900

H 1.17785300 2.01137100 2.36971600

H -0.61233400 2.20717900 2.40059600

Cl 0.24643100 2.12169600 0.20126100

**TS: Fe(CO)_4_ + CH_3_Cl(95°)**

***E*** = -2146.03

**H** = -2092.60

**G** = -2127.64

***N*_imag_** = 1, (-489.51 cm^–1^

Fe -0.01563198 -0.12634601 -0.14757949

O -0.09473177 -0.70192816 -3.00642659

C -0.04879301 -0.35888883 -1.90660045

O -0.34933361 -2.93020087 0.39730233

O -2.89960276 0.21555512 0.25548069

O 2.86928505 -0.47469447 0.24474694

C 1.73362692 -0.32751218 0.11030863

C -1.76167988 0.09096549 0.11730628

C -0.21422024 -1.79383328 0.22790991

C 0.15265020 1.24977953 2.07544021

H -0.69453841 1.86824785 2.35755879

H 1.11826583 1.65974382 2.35711673

H 0.03330625 0.21062373 2.35839591

Cl 0.26201861 2.18286591 0.12548332

**P: Fe(CO)_4_ + CH_3_Cl(95°)**

***E*** = -2187.15

**H** = -2132.94

**G** = -2167.49

***N*_imag_** = 0

Fe -0.00653200 -0.04484000 0.11853000

O -0.03170200 -0.19989200 -2.80701100

C -0.01989100 -0.12358300 -1.66336900

O -0.35464000 -2.88081900 0.50442000

O -2.82930300 0.65157900 0.47134700

O 2.90168600 -0.06673500 0.46375600

C 1.76771200 -0.07326300 0.31110700

C -1.73257500 0.36443100 0.31643100

C -0.21656100 -1.74925100 0.34656100

C 0.01348200 0.07734600 2.21339700

H -0.92648300 -0.28062300 2.63937300

H 0.15244200 1.13890100 2.42097300

H 0.83115200 -0.51035100 2.63662700

Cl 0.27514800 2.23117800 -0.05228100

**RC: Fe(CO)_4_ + CH_3_Cl(100°)**

***E*** = -2178.63

**H** = -2124.73

**G** = -2159.28

***N*_imag_** = 0

Fe -0.02018700 -0.14600000 -0.20340000

O -0.01554300 -0.00972600 -3.13426300

C -0.01088500 -0.01487100 -1.97900400

O -0.33885500 -2.97776000 0.10846900

O -2.70259100 0.00933900 0.95852500

O 2.62922200 -0.59797500 0.95626900

C 1.58679800 -0.39718900 0.49307600

C -1.64150100 -0.02934800 0.49591100

C -0.21099700 -1.83599000 -0.03156200

C 0.32159100 2.71302500 1.67270300

H 0.45280100 3.79717800 1.65247800

H 1.17246900 2.22138300 2.14527300

H -0.61560300 2.43785800 2.15745700

Cl 0.24656600 2.18568600 -0.02835400

**TS: Fe(CO)_4_ + CH_3_Cl (100°)**

***E*** = -2146.75

**H** = -2093.30

**G** = -2128.31

***N*_imag_** = 1, -493.66 cm^–1^

Fe -0.01519766 -0.11964733 -0.14151694

O -0.08649208 -0.63429073 -3.00852969

C -0.04489027 -0.32415865 -1.89854400

O -0.34105617 -2.87967424 0.61028945

O -2.90916140 0.23158917 0.15806448

O 2.88097297 -0.46197575 0.14804960

C 1.74171929 -0.31901209 0.04959187

C -1.76868626 0.10126653 0.05634777

C -0.20920111 -1.75899283 0.35392606

C 0.14766139 1.19958580 2.10050223

H -0.69539907 1.81717832 2.39609860

H 1.11541927 1.59704448 2.39210931

H 0.02140401 0.15654104 2.36487404

Cl 0.26273005 2.18888702 0.17685673

**P: Fe(CO)_4_ + CH_3_Cl(100°)**

***E*** = -2186.90

**H** = -2132.69

**G** = -2167.19

***N*_imag_** = 0

Fe -0.01488900 -0.11360000 0.15071500

O -0.06089000 -0.23889400 -2.77728700

C -0.04320700 -0.19441000 -1.63223500

O -0.36051700 -2.89567100 0.82126400

O -2.83819900 0.58933900 0.48567700

O 2.90009700 -0.15051500 0.43198600

C 1.76293300 -0.15151900 0.30566400

C -1.74110500 0.29950300 0.33897600

C -0.22462800 -1.79021900 0.53113800

C 0.03359100 0.10399500 2.23869700

H -0.90206700 -0.22450600 2.69675000

H 0.18073500 1.17443800 2.38533700

H 0.85482800 -0.46424300 2.68121800

Cl 0.27439500 2.15580900 -0.11040900

**RC: Fe(CO)_4_ + C_2_H_6_(90°)**

***E*** = -2578.04

**H** = -2510.55

**G** = -2546.36

***N*_imag_** = 0

Fe 0.11710300 -0.02981400 -0.06987000

O 0.13534800 -2.18712800 -2.05110400

C 0.13496200 -1.30213400 -1.30648800

O -0.26768900 -2.00807500 1.95914600

O -2.46513200 1.09548200 0.74307300

O 2.61945800 0.65561700 1.29289200

C 1.63743800 0.41796700 0.73028500

C -1.44062200 0.68788200 0.39374300

C -0.11413500 -1.20793900 1.13892800

C -0.40799194 2.53242597 -3.00154774

H -0.22916194 1.96777497 -3.92227574

H -0.24978294 3.59446397 -3.22334574

H -1.45746494 2.40346197 -2.72187674

C 0.52912806 2.10410997 -1.88949774

H 0.34334706 2.63740397 -0.95749374

H 1.58550506 2.22550897 -2.14807174

H 0.43309606 0.96536397 -1.81812474

**TS: Fe(CO)_4_ + C_2_H_6_(90°)**

***E*** = -2538.34

**H** = -2462.31

**G** = -2496.35

***N*_imag_** = 1, -657.23 cm^–1^

Fe -0.00732832 0.15526367 -0.09575061

O 0.04804761 -2.56859107 -1.06802977

C 0.01989896 -1.43928519 -0.81453850

O 0.04614980 -1.24159540 2.43650725

O -2.93396418 0.19769384 -0.11657449

O 2.91295999 0.34513466 -0.15470934

C 1.76108671 0.26522329 -0.13453133

C -1.77937352 0.17908719 -0.10330055

C 0.02742576 -0.56289259 1.49871375

C -0.01395568 2.41947272 0.07272796

H -0.31212715 2.14590741 1.08551822

H -0.71559530 3.18588226 -0.26005417

H 0.99571710 2.82597637 0.09851881

C -0.11045024 1.73165010 -1.72656113

H 0.54079549 2.56297441 -2.00065898

H -1.13967879 1.96145434 -1.99720845

H 0.22564091 0.87133116 -2.30645964

**P: Fe(CO)_4_ + C_2_H_6_(90°)**

***E*** = -2578.46

**H** = -2501.95

**G** = -2536.73

***N*_imag_** = 0

Fe -0.00624700 0.08413400 -0.03024600

O 0.03391600 -2.64777000 -1.03660700

C 0.01713800 -1.55862300 -0.66333800

O 0.06148900 -0.99728400 2.67251300

O -2.85769300 0.63239900 -0.24126700

O 2.81150200 0.75038500 -0.33322900

C 1.70681400 0.45824000 -0.20317000

C -1.73808900 0.38698900 -0.14707100

C 0.03423700 -0.54838700 1.61252400

C -0.03055700 2.02590700 0.78531300

H -0.90908100 2.16163300 1.42051200

H -0.05329500 2.74792300 -0.03257400

H 0.85774100 2.19297400 1.39911500

C -0.05204200 0.79095000 -2.01208700

H -0.07195100 1.88191200 -1.99348900

H -0.93748500 0.41578500 -2.53073400

H 0.82943100 0.44864700 -2.55955900

**RC: Fe(CO)_4_ + C_2_H_6_(95°)**

***E*** = -2581.01

**H** = -2506.54

**G** = -2539.80

***N*_imag_** = 0

Fe -0.15935700 -2.02060600 1.21305500

O -0.60399700 -4.02690600 -0.78026400

C -0.35190100 -3.12001600 -0.10479700

O -1.41943412 -3.92895684 2.93517394

O -2.70013500 -0.67174400 0.61946600

O 2.52805000 -3.03453500 1.82777500

C 1.47130600 -2.64205900 1.58664100

C -1.70604500 -1.20700200 0.85217700

C -0.89659574 -3.05576273 2.38151548

C 4.20655290 4.27338289 3.58284405

H 5.24550090 4.56345489 3.77847505

H 3.56708590 5.09090389 3.93563305

H 4.08451290 4.20896789 2.49529105

C 3.85786790 2.95948389 4.26205205

H 2.81793390 2.67260389 4.06730005

H 3.98161190 3.02294089 5.34948905

H 4.49662690 2.14192689 3.90797305

**TS: Fe(CO)_4_ + C_2_H_6_(95°)**

***E*** = -2540.12

**H** = -2464.03

**G** = -2498.10

***N*_imag_** = 1, -653.85 cm^–1^

Fe -0.00874923 0.15832017 -0.09364765

O 0.04196371 -2.51681264 -1.19352713

C 0.01556186 -1.40562931 -0.86991774

O 0.05382030 -1.08313874 2.51870585

O -2.93474752 0.17865037 -0.11468811

O 2.91197581 0.32282576 -0.14658094

C 1.75949640 0.25561137 -0.13018420

C -1.78029823 0.17091777 -0.10157475

C 0.02940056 -0.47809341 1.53208983

C -0.02142249 2.42560730 0.07363665

H -0.34144678 2.16684869 1.08302712

H -0.70961570 3.19365921 -0.28209262

H 0.99153380 2.82284150 0.11177287

C -0.10198175 1.72915400 -1.73390699

H 0.54957922 2.56385731 -1.99619380

H -1.13015866 1.95869640 -2.00872447

H 0.23997514 0.87395421 -2.31700567

**P: Fe(CO)_4_ + C_2_H_6_(95°)**

***E*** = -2578.80

**H** = -2502.28

**G** = -2537.10

***N*_imag_** = 0

Fe -0.00870000 0.06733800 -0.06007000

O -0.00158500 -2.53360000 -1.36724600

C -0.00440100 -1.50374500 -0.85240400

O 0.19930200 -1.00816300 2.63694900

O -2.87656800 0.56734200 -0.10496200

O 2.78986300 0.76461000 -0.45682400

C 1.69353000 0.46008600 -0.29056500

C -1.74960500 0.34055400 -0.07659600

C 0.11634400 -0.58279200 1.57030600

C -0.01785000 1.96867300 0.85066100

H -0.86289500 2.06122000 1.53708700

H -0.09544700 2.72581600 0.06886300

H 0.89952000 2.12464200 1.42335700

C -0.15401700 0.89116800 -1.99432000

H -1.06349900 0.54571300 -2.49163800

H -0.17462200 1.97851300 -1.90612200

H 0.69964600 0.58838500 -2.60553200

**RC: Fe(CO)_4_ + C_2_H_6_(100°)**

***E*** = -2583.56

**H** = -2508.00

**G** = -2545.08

***N*_imag_** = 0

Fe -0.15935700 -2.02060600 1.21305500

O -0.60399700 -4.02690600 -0.78026400

C -0.35190100 -3.12001600 -0.10479700

O -1.38842000 -3.76532600 3.12110300

O -2.70013500 -0.67174400 0.61946600

O 2.52805000 -3.03453500 1.82777500

C 1.47130600 -2.64205900 1.58664100

C -1.70604500 -1.20700200 0.85217700

C -0.87134400 -2.94783700 2.48336500

C 4.86661285 5.89653438 1.41233822

H 5.90556085 6.18660638 1.60796922

H 4.22714585 6.71405538 1.76512722

H 4.74457285 5.83211938 0.32478522

C 4.51792785 4.58263538 2.09154622

H 3.47799385 4.29575538 1.89679422

H 4.64167185 4.64609238 3.17898322

H 5.15668685 3.76507838 1.73746722

**TS: Fe(CO)_4_ + C_2_H_6_(100°)**

***E*** = -2541.19

**H** = -2465.06

**G** = -2499.20

***N*_imag_** = 1, -649.65cm^–1^

Fe -0.00852203 0.17139588 -0.06770483

O 0.04015760 -2.50068038 -1.17858786

C 0.01444582 -1.39652878 -0.83208027

O 0.04592144 -0.78815794 2.66242425

O -2.93342854 0.16836564 -0.08257897

O 2.91247741 0.31535020 -0.11860615

C 1.75973004 0.25755389 -0.10318139

C -1.77917884 0.17212697 -0.07299669

C 0.02648556 -0.30852661 1.60915279

C -0.02826199 2.44740195 -0.01086843

H -0.36118967 2.25034757 1.00773462

H -0.71008593 3.19370362 -0.42065972

H 0.98557564 2.84348734 0.01448374

C -0.09304679 1.65697740 -1.79011957

H 0.55008792 2.48853982 -2.08112762

H -1.12224093 1.86285036 -2.07950398

H 0.26394411 0.78387892 -2.33563422

**P: Fe(CO)_4_ + C_2_H_6_(100°)**

***E*** = -2578.45

**H** = -2501.92

**G** = -2536.81

***N*_imag_** = 0

Fe -0.00577800 0.08425600 -0.02855900

O 0.02680100 -2.51358900 -1.34040800

C 0.01407700 -1.49533500 -0.80307900

O 0.05455100 -0.69032000 2.77635000

O -2.86752900 0.58703300 -0.21837500

O 2.82466900 0.70542400 -0.31282000

C 1.71466300 0.43173800 -0.19010000

C -1.74356100 0.36023900 -0.13282500

C 0.03119500 -0.40366000 1.66148900

C -0.03175400 2.04193400 0.75899000

H -0.90898900 2.19198100 1.39295200

H -0.05798400 2.74465200 -0.07501400

H 0.85774700 2.22632600 1.36627100

C -0.05272700 0.80880200 -2.00942000

H -0.07428600 1.89894300 -1.97362500

H -0.93786400 0.44161800 -2.53462800

H 0.82906200 0.47757900 -2.56345700

**RC: Fe(CO)_2_(PH_2_CH_2_PH_2_) + CH_4_**

***E*** = -2415.75

**H** = -2327.10

**G** = -2360.80

***N*_imag_** = 0

H 1.16276600 1.79929300 1.94772600

P -0.98361400 -0.47970400 1.56207000

P -1.08896400 -0.59269200 -0.99514600

O 0.11737400 2.71419800 -1.29560200

H -0.93956900 -1.67055200 2.35429000

Fe 0.48527600 0.19031700 0.10102900

H 2.88589000 1.76218200 1.48406500

O 2.26433000 -1.64463400 -1.28114400

H -1.76363900 0.13108300 -2.02055100

H -1.67713300 0.27213400 2.56576500

H -0.98886300 -1.83212400 -1.68784600

H 2.27629200 0.69804700 2.80293100

H -2.74878100 -1.88781500 0.39049200

C 2.04408100 1.16961000 1.84494800

C -2.31335000 -0.88527400 0.33147200

C 1.59325600 -0.91023600 -0.66908000

C 0.28511800 1.74092900 -0.67286700

H -3.10471800 -0.12932800 0.33134300

H 1.97001500 0.28486300 1.13567700

**TS: Fe(CO)_2_(PH_2_CH_2_PH_2_) + CH_4_**

***E*** = -2401.66

**H** = -2317.39

**G** = -2350.94

***N*_imag_** = 1, -833.20 cm^–1^

H 1.27187182 1.66937786 2.08604174

P -1.01326424 -0.47273896 1.46106326

P -1.06810001 -0.61752172 -1.12823422

O -0.21017396 2.83689915 -0.95519503

H -0.98012543 -1.69185979 2.20664105

Fe 0.50106260 0.21207425 0.04958408

H 2.79275470 1.41017481 1.23816225

O 2.30289093 -1.86843038 -0.87205739

H -1.74061168 0.01682262 -2.21528089

H -1.67513454 0.27578342 2.48525957

H -0.98249634 -1.91323857 -1.71100218

H 2.08863435 0.06504851 2.12774081

H 1.80323550 0.89108256 -0.09120439

C 1.84189660 0.93413844 1.51497340

C -2.34014800 -0.80801421 0.19986955

C 1.57667153 -1.05950989 -0.45504969

C 0.05004177 1.79500595 -0.50265158

H -3.04687407 0.02834338 0.16684869

H -2.88496369 -1.75572986 0.25847435

**P: Fe(CO)_2_(PH_2_CH_2_PH_2_) + CH_4_**

***E*** = -2413.34

**H** = -2328.37

**G** = -2361.95

***N*_imag_** = 0

H 1.39977402 1.69763888 1.88977954

P -0.98143280 -0.43834841 1.39910902

P -1.16838110 -0.68443738 -1.16370375

O -0.15713580 2.96543905 -0.58304613

H -0.89379009 -1.63012450 2.17827559

Fe 0.47564225 0.18561770 -0.09287602

H 2.71037268 1.33538938 0.74710672

O 2.48113079 -1.86205876 -0.51408347

H -1.91656735 -0.13228232 -2.24724252

H -1.54640531 0.37296944 2.42904443

H -1.15997027 -2.02346520 -1.65002970

H 2.20405848 0.11461304 1.93338366

H 1.18615453 0.61954786 -1.33019576

C 1.85036831 0.91313326 1.27305744

C -2.37971690 -0.77273047 0.23830891

C 1.64928786 -1.08222693 -0.30829477

C 0.05027289 1.84873531 -0.34774927

H -3.04208744 0.09926184 0.20863845

H -2.97046865 -1.68414306 0.36871181

**RC: Fe(CO)_2_(PH_2_CH_2_CH_2_PH_2_) + CH_4_**

***E*** = -2805.90

**H** = -2697.80

**G** = -2734.28

***N*_imag_** = 0

H 2.37373800 1.21219200 1.05155400

P -0.27202100 -0.10558600 1.58755700

P -1.30210500 0.00945900 -1.09176100

O 0.94099500 2.53969900 -1.79525100

H 0.03705000 -1.10480800 2.56080300

H -3.56889100 -0.00226500 -0.12737200

H -2.61557600 1.42418400 0.33445300

O 1.05879400 -2.45401000 -1.83115100

H -1.70551200 0.90957400 -2.11433600

H -0.21443100 0.96424000 2.53879400

H -1.80965500 -1.17495000 -1.69661800

C -2.10717100 -0.34706100 1.47565700

H -2.27843100 -1.42884100 1.42057200

H -2.61585100 0.02952600 2.36907000

C -2.57939000 0.33572400 0.20337700

C 0.93684400 -1.47854700 -1.19930000

C 0.84671100 1.55202900 -1.17700100

Fe 0.64274300 0.02425400 -0.36474100

H 3.74795000 0.60147400 0.10066900

H 3.18834800 -0.29690600 1.55855200

C 2.88588700 0.31652400 0.70722600

H 2.31743200 -0.40807600 0.03178400

**TS: Fe(CO)_2_(PH_2_CH_2_CH_2_PH_2_) + CH_4_**

***E*** = -2796.52

**H** = -2692.99

**G** = -2727.53

***N*_imag_** = 1, -811.69 cm^–1^

H 2.50484964 1.03675704 1.26966792

P -0.36078853 -0.03018651 1.54234690

P -1.25903972 -0.03400211 -1.23228591

O 0.83500156 2.78395905 -1.28840735

H -0.06430184 -0.98675055 2.55918793

H -3.57218170 -0.07956047 -0.35055430

H -2.67745632 1.38770136 0.09074689

O 1.14106838 -2.63598486 -1.34467099

H -1.65995010 0.83681920 -2.28428155

H -0.36445124 1.09248296 2.42628649

H -1.71949507 -1.24145329 -1.82957413

C -2.18785719 -0.31844403 1.33551012

H -2.32769771 -1.40655279 1.31779626

H -2.76138226 0.08021198 2.17896658

C -2.60641962 0.29564370 0.00727344

C 0.94110681 -1.57801659 -0.90123831

C 0.75490856 1.70087814 -0.86595032

Fe 0.65877169 0.04668609 -0.34111403

H 3.48616215 0.19310778 0.08151985

H 2.05165088 0.13644220 -0.83182298

C 2.54334916 0.14175446 0.64576049

H 2.59602653 -0.76041751 1.25803389

**P: Fe(CO)_2_(PH_2_CH_2_CH_2_PH_2_) + CH_4_**

***E*** = -2807.98

**H** = -2703.78

**G** = -2738.41

***N*_imag_** = 0

H 2.52202800 1.01191200 1.12154100

P -0.32815700 -0.02300000 1.46331800

P -1.38479800 -0.02993400 -1.24508100

O 1.05373100 2.84718600 -1.01756500

H 0.05170100 -0.96235800 2.46477400

H -3.64610800 -0.08945400 -0.22635000

H -2.73277300 1.37829500 0.16838200

O 1.35946400 -2.68438400 -1.05860600

H -1.85072000 0.84117700 -2.27144300

H -0.28526400 1.12067200 2.31422000

H -1.89320500 -1.22749200 -1.82853100

C -2.15775800 -0.33476500 1.36435000

H -2.28374400 -1.42476700 1.34225200

H -2.68245800 0.04511500 2.24772900

C -2.66090400 0.28756700 0.06988900

C 1.01752100 -1.61221600 -0.77877100

C 0.83269500 1.73941900 -0.75350600

Fe 0.59006600 0.04343200 -0.46827000

H 3.22561400 0.19136100 -0.28650700

H 1.21729200 0.10268300 -1.82207300

C 2.44425400 0.13128700 0.47561700

H 2.62343900 -0.75863200 1.08729200

**RC: Fe(CO)_2_(PH_2_CH_2_CH_2_CH_2_PH_2_) + CH_4_**

***E*** = -3184.53

**H** = -3058.50

**G** = -3095.78

***N*_imag_** = 0

H 1.85243600 2.39678300 0.26843500

P -0.47526700 0.91033400 1.40914100

P -0.75352800 -1.28484000 -0.57467100

O 0.58136700 0.96065200 -2.81461300

H -0.68773500 0.30636500 2.68757800

H -4.05609000 0.27118300 0.38512600

H -3.01704300 -0.74203900 1.36511000

O 2.25628100 -2.40273300 0.64350800

H -0.66604400 -2.00490000 -1.79887000

H -0.15515200 2.19400000 1.94890700

H -0.98949500 -2.40567500 0.27000600

C -3.00468000 -0.00073000 0.55374200

H -2.74110900 1.68166300 1.88339300

H -2.24990200 1.99815500 0.21269300

C -2.46880300 -0.64712200 -0.72223600

C 1.69314800 -1.41110700 0.39006400

C 0.66826700 0.63282400 -1.69978700

Fe 0.79304800 0.00568600 -0.07814300

H 3.52481900 1.91732800 -0.06665700

H -3.10402700 -1.49332200 -1.01564100

C 2.61498800 1.65557200 0.48313400

H 2.82243800 1.57899500 1.54905800

C -2.24917000 1.24261900 1.00837700

H -2.47105400 0.06815800 -1.55400100

H 2.44493200 0.60405700 0.05944100

**TS: Fe(CO)_2_(PH_2_CH_2_CH_2_CH_2_PH_2_) + CH_4_**

***E*** = -3176.38

**H** = -3054.17

**G** = -3090.40

***N*_imag_** = 1, -799.63 cm^–1^

H 1.93146351 2.40802351 0.38022535

P -0.56948997 0.95753579 1.36663792

P -0.65520537 -1.30848637 -0.74771394

O 0.43083824 1.46210734 -2.57617939

H -0.65889769 0.44972259 2.69898528

H -4.05627200 -0.17110511 0.44586857

H -2.82094838 -1.04528236 1.32574729

O 2.14624139 -2.17380682 1.17829187

H -0.55575683 -1.84808004 -2.06553567

H -0.36993826 2.31603821 1.76277412

H -0.77111185 -2.55759630 -0.07496498

C -2.96803335 -0.29017286 0.54008909

H -2.91174409 1.41846841 1.87129387

H -2.49381963 1.79564453 0.19680378

C -2.43327599 -0.81867671 -0.78964373

C 1.58163945 -1.26358591 0.72113151

C 0.54197701 0.93903558 -1.53972725

Fe 0.78013447 0.07695043 -0.04856882

H 3.33931414 1.39140264 0.09895323

H 2.18503197 0.28572218 -0.47855610

C 2.33758079 1.41014874 0.55289795

H 2.47565215 1.22380582 1.61946624

C -2.37727666 1.04526791 0.98978282

H -2.53338830 -0.05692682 -1.57362653

H -3.01112945 -1.69349688 -1.11301645

**P: Fe(CO)_2_(PH_2_CH_2_CH_2_CH_2_PH_2_) + CH_4_**

***E*** = -3187.61

**H** = -3064.61

**G** = -3100.87

***N*_imag_** = 0

H 1.95065400 2.34222135 0.29377371

P -0.53769489 0.88993697 1.32185123

P -0.75811940 -1.36858047 -0.73394381

O 0.55249532 1.72617829 -2.42793678

H -0.62878811 0.31701349 2.62474651

H -4.09543942 -0.03698416 0.44298656

H -2.92054102 -0.97356258 1.34141633

O 2.32593016 -1.94033624 1.36812333

H -0.69266980 -1.96639071 -2.02706946

H -0.26637607 2.21893486 1.76424080

H -0.92146968 -2.58953329 -0.01660026

C -3.01777242 -0.22805819 0.53924887

H -2.83174217 1.48996011 1.84669588

H -2.42078550 1.82181435 0.16186682

C -2.51997761 -0.82076721 -0.77737469

C 1.66203922 -1.16854429 0.81312510

C 0.58182865 1.05037911 -1.48416751

Fe 0.76203238 -0.01567470 -0.12509956

H 3.16886875 1.10801436 -0.09183761

H 1.71427141 -0.59142212 -1.11896473

C 2.25539970 1.30656773 0.47443617

H 2.47822390 1.19902528 1.54122406

C -2.33786240 1.07324804 0.96095410

H -2.59902984 -0.07984097 -1.58373068

H -3.13861094 -1.67926309 -1.06495878

**RC: Fe(CO)_2_(PH_2_CH_2_CH_2_CH_2_CH_2_PH_2_) + CH_4_**

***E*** = -3559.98

**H** = -3414.94

**G** = -3454.60

***N*_imag_** = 0

H -2.25693200 1.57186700 -1.01631500

P 0.01264300 -0.45184400 1.84923800

P -1.00076700 -0.45373100 -0.88571600

H 2.61156100 -0.12944200 0.33404600

H -0.76415300 -1.64254900 1.96049300

H -3.92069700 1.29538700 0.80120800

H -3.21329400 -0.21963700 1.29374600

C 0.80385800 1.48331200 -0.93182400

H -1.09762500 -0.53234500 -2.30272100

H 0.84858700 -0.76406000 2.96393700

H -1.54711600 -1.73787300 -0.59957100

C -2.96434300 0.75961200 0.86267500

H -2.64465500 2.18758500 2.44362400

H -1.39993400 2.20965800 1.21081800

C -2.48299600 0.59115200 -0.57932700

O 1.76722100 -2.38179000 -1.55035200

H 3.46219700 0.18579700 1.84089400

C 1.45170600 -1.45249800 -0.91356400

C -2.03309100 1.53825100 1.80316500

H -3.28272900 0.14807800 -1.18971700

C -1.14325200 0.71054900 2.72469500

H -0.50759900 1.39084000 3.30640200

H -1.74085900 0.13963700 3.44713200

H 2.36426700 1.49038300 1.30497000

Fe 0.86910200 -0.03670700 -0.08487900

C 3.04225500 0.70574500 0.97702500

O 0.74685200 2.45321100 -1.58655700

H 3.82757300 1.13724900 0.35365300

**TS: Fe(CO)_2_(PH_2_CH_2_CH_2_CH_2_CH_2_PH_2_) + CH_4_**

***E*** = -3553.38

**H** = -3410.27

**G** = -3448.42

***N*_imag_** = 1, -799.67cm^–1^

H -2.22078500 1.40622600 -1.17655700

P 0.12985500 -0.44633900 1.85637600

P -1.17676300 -0.74377200 -0.95599800

H 2.98612300 -1.27402400 1.09098300

H -0.67656500 -1.50693600 2.36060200

H -3.75867400 1.38254400 0.82299300

H -3.01290700 -0.08403900 1.39387800

C 0.88421900 1.00647900 -0.91936600

H -1.36337600 -0.85428000 -2.36416400

H 1.10316000 -0.53923900 2.89445100

H -1.88767300 -1.92802700 -0.61722200

C -2.80747900 0.83441600 0.82744100

H -2.27583700 2.48215600 2.11202300

H -1.14770200 2.21962800 0.80687700

C -2.50336200 0.49561600 -0.63328700

C 0.93733500 -2.27087800 -0.50180800

Fe 0.78889700 -0.56883900 -0.18787300

O 1.05162700 -3.39592100 -0.78331300

C -1.75722000 1.69411700 1.54990000

H -3.40455800 0.10457900 -1.12387600

C -0.83146600 0.98787800 2.53656900

H -0.08714200 1.70911600 2.89713500

H -1.38526800 0.64189400 3.41842200

H 2.92559500 0.51425200 0.91098500

H 3.66539400 -0.48809900 -0.32920500

H 2.07669000 -0.62396800 -0.92037400

O 0.99372700 2.01513200 -1.49599700

C 2.84083300 -0.44468100 0.39738300

**P: Fe(CO)_2_(PH_2_CH_2_CH_2_CH_2_CH_2_PH_2_) + CH_4_**

***E*** = -3566.14

**H** = -3422.75

**G** = -3459.41

***N*_imag_** = 0

H -1.96557400 1.99643200 -0.50962600

P 0.28934700 0.08492700 1.93594000

P -1.02256200 -0.16078800 -0.99422700

O 1.39758600 -2.95919700 -0.39667500

H 0.91127200 -0.79435100 2.86333100

H -4.04361400 1.34354700 0.75184800

H -3.52904000 -0.32489100 0.76545800

H 3.01607900 -0.90757300 1.36521500

H -1.08212100 0.04623600 -2.40030300

H 0.66338800 1.28581200 2.60064900

H -1.75603500 -1.38066200 -1.00365300

C -3.14853900 0.70638900 0.77482900

H -3.23973100 1.02337400 2.86825600

H -1.99906000 1.97707000 2.09481300

C -2.39407200 0.98594500 -0.52334400

C 1.16852700 -1.83366400 -0.23125500

Fe 0.91163600 -0.12002400 -0.10847500

C 1.23367300 1.51520700 -0.59508400

C -2.44978400 0.97461300 2.10652400

H -3.10950200 0.96004000 -1.35523500

C -1.43121200 -0.05618700 2.59547400

H -1.34226000 0.01015200 3.68737000

H -1.77802800 -1.07466100 2.37560500

H 3.01225300 0.86321000 1.25584900

C 2.83312800 -0.06099900 0.69526400

H 3.55800700 -0.10879800 -0.12159000

O 1.51498200 2.56800000 -0.99633900

H 1.54857000 -0.27199200 -1.44591100

**RC: Fe(CO)_2_(PH_2_CH_2_PH_2_) + CH_3_Cl**

***E*** = -2384.89

**H** = -2300.60

**G** = -2334.72

***N*_imag_** = 0

H 1.89795800 2.81131900 1.01807800

P -0.29292500 -0.26348200 1.55558800

P -1.43611200 -0.64150800 -0.70085100

O -0.26072500 2.46008200 -1.94399600

H -0.01820100 -1.37608200 2.41444800

Fe 0.50209200 0.08488300 -0.45144600

H 3.67367700 2.54038600 1.22625100

O 1.50024700 -2.13610600 -2.01560600

H -2.43389500 0.01979200 -1.47365100

H -0.47555300 0.64125900 2.65435200

H -1.70043600 -1.96696200 -1.14769300

H 2.49380300 1.69133500 2.29436400

H -2.49009900 -1.54357300 1.41001400

C 2.67667400 2.10088800 1.29986300

C -2.03642000 -0.62288300 1.02881600

C 1.14845400 -1.24664000 -1.34470400

C 0.08901900 1.54470100 -1.30669100

H -2.69270400 0.23126200 1.22281200

Cl 2.65274400 0.79224600 0.09497800

**TS: Fe(CO)_2_(PH_2_CH_2_PH_2_) + CH_3_Cl**

***E*** = -2352.36

**H**  = -2268.40

**G** = -2304.02

***N*_imag_** = 1, -493.33 cm^–1^

C 2.87474500 1.39265700 -0.39126300

P -1.04633100 -0.50635200 1.42625400

P -0.95774000 -0.56171000 -1.16186900

O -0.41760100 2.80282100 -0.80128800

H -1.11650900 -1.79791800 2.02774900

Fe 0.54947600 0.18976700 0.07595100

H 2.93942000 2.45925200 -0.19820100

O 2.14492700 -2.11549100 -0.74476000

H -1.50725400 0.08353100 -2.30748700

H -1.76044800 0.14773700 2.47918700

H -0.88262700 -1.87005900 -1.70961700

Cl 2.12301400 1.00379200 1.59062800

H 3.78955000 0.84368500 -0.18929400

H 2.44161100 1.15922500 -1.35574300

C -2.31318100 -0.66642100 0.07396400

C 1.54588800 -1.18548300 -0.37704500

C -0.00202500 1.78679200 -0.40445700

H -2.91256100 0.24951400 0.03241500

H -2.96563800 -1.54530000 0.06085200

**P: Fe(CO)_2_(PH_2_CH_2_PH_2_) + CH_3_Cl**

***E*** = -2399.22

**H**  = -2314.73

**G**  = -2349.36

***N*_imag_** = 0

H 1.52638700 1.80902600 1.72351600

P -0.83028200 -0.42482500 1.32020300

P -1.18141200 -0.63265300 -1.24745000

O -0.46178600 2.98392800 -0.12667200

H -0.60693300 -1.61027000 2.07047200

Fe 0.49706400 0.23313700 -0.18958300

H 2.70897600 1.43443400 0.43677400

O 2.26171900 -2.08296600 -0.05669900

H -1.99244000 -0.00373800 -2.23042500

H -1.34579500 0.36697600 2.38415300

H -1.16704100 -1.92707500 -1.83109600

H 2.37239100 0.25505600 1.73606200

Cl 1.62495700 0.79272800 -2.11542800

C 1.93497400 1.01896700 1.08616000

C -2.27229000 -0.83146200 0.24095900

C 1.54873800 -1.17966500 -0.12332200

C -0.10265400 1.88821800 -0.16662600

H -3.01730800 -0.02941800 0.27949700

H -2.76223400 -1.79745300 0.39311000

**RC: Fe(CO)_2_(PH_2_CH_2_CH_2_PH_2_) + CH_3_Cl**

***E*** = -2776.92

**H**  = -2673.11

**G**  = -2709.77

***N*_imag_** = 0

Cl 2.06615000 0.34989000 -0.90905300

P -1.70507000 -0.27658900 1.24067100

P -1.02854500 0.12689600 -1.52571500

O 0.33871100 2.47338600 1.86119100

H -1.86415100 -1.30146400 2.21229000

H -3.41932900 -0.24632500 -2.04404300

H -3.13580900 1.18267700 -1.03276200

O 0.86787900 -2.57790300 1.33863600

H -0.95925700 1.19256300 -2.47466300

H -2.28293400 0.77255600 2.00954400

H -0.96511200 -0.89334100 -2.52747900

C -3.08485200 -0.67681300 0.07446000

H -3.00981900 -1.75126400 -0.13404200

H -4.06004000 -0.48569500 0.53770900

C -2.85243400 0.13465200 -1.18814500

C 0.59604900 -1.54022000 0.86988200

C 0.27765000 1.51800400 1.18831000

Fe 0.12103100 0.03183700 0.28808500

H 4.35280400 0.63202700 -0.46970000

H 3.31901200 1.38039700 0.80442800

H 3.54513800 -0.40352800 0.76668200

C 3.47632300 0.50705700 0.17026800

**TS: Fe(CO)_2_(PH_2_CH_2_CH_2_PH_2_) + CH_3_Cl**

***E*** = -2742.73

**H**  = -2642.22

**G**  = -2680.03

***N*_imag_** = 1, -508.11 cm^–1^

Cl 2.17889366 0.38779437 -0.81347506

P -1.54185386 -0.28499714 1.47402058

P -1.15138545 0.08331313 -1.41093472

O -0.00792091 2.80939594 1.23581348

H -1.61556459 -1.32202670 2.44752766

H -3.59568093 -0.29375366 -1.64004731

H -3.19139017 1.15017974 -0.69394885

O 0.62476508 -2.81702449 0.67234558

H -1.18396557 1.11840484 -2.39469511

H -2.05341002 0.75686279 2.29883304

H -1.18355692 -0.98488754 -2.35967591

C -3.02715950 -0.69360385 0.43167960

H -2.97096550 -1.77156874 0.23342129

H -3.95346583 -0.49854117 0.98422415

C -2.93018700 0.09820787 -0.86411380

C 0.47678168 -1.67109211 0.50972662

C 0.09429205 1.71373869 0.85171784

Fe 0.15078398 0.04199323 0.29774749

H 3.25480634 1.05494320 1.24098050

H 1.94400555 0.15386098 2.09962095

H 3.16989041 -0.76353251 1.13383620

C 2.62702363 0.16834611 1.26012243

**P: Fe(CO)_2_(PH_2_CH_2_CH_2_PH_2_) + CH_3_Cl**

***E*** = -2793.72

**H**  = -2689.70

**G**  = -2726.78

***N*_imag_** = 0

Cl 1.82982091 0.38969377 -1.27324032

P -1.47328758 -0.28271819 1.54452951

P -1.10498842 0.11478933 -1.32620928

O 0.26685917 2.90781881 0.93892107

H -1.50028755 -1.28134113 2.55936460

H -3.52853796 -0.38870295 -1.56153435

H -3.19950187 1.06996725 -0.61311691

O 0.85347172 -2.78477489 0.28981756

H -1.11108598 1.21788559 -2.21751867

H -2.00645800 0.77693101 2.33266453

H -1.02667231 -0.91645439 -2.29979878

C -2.93653075 -0.76357554 0.51208357

H -2.82608874 -1.83726297 0.31248455

H -3.87345414 -0.61959796 1.06200049

C -2.88150127 0.03379051 -0.78522912

C 0.59051518 -1.66077847 0.29167259

C 0.23913998 1.78335903 0.68164355

Fe 0.21992059 0.05556445 0.35098816

H 2.55949964 0.13781458 1.45948809

H 1.40991773 0.80409329 2.65317662

H 1.56132519 -0.94740630 2.46860397

C 1.58169981 0.00345667 1.92762404

**RC: Fe(CO)_2_(PH_2_CH_2_CH_2_CH_2_PH_2_) + CH_3_Cl**

***E*** = -3155.56

**H**  = -3033.23

**G**  = -3071.57

***N*_imag_** = 0

H 3.55305100 1.22580400 -1.76988100

P -0.72671100 0.53390700 1.20985400

P -0.60983100 -1.54240000 -0.92108000

O 0.59368500 2.56321000 -1.23831500

H -0.98442000 -0.23818900 2.37799500

H -4.10944400 -0.28645500 -0.24925000

H -3.10433900 -1.29094000 0.77463000

O 2.25732900 -0.75561600 2.31691200

H -0.34728200 -2.14204000 -2.19094800

H -0.55268900 1.78261700 1.87149500

H -0.89199700 -2.78589200 -0.27361200

C -3.06360700 -0.50395200 0.00811700

H -3.04815600 1.11306200 1.45107300

H -2.41722500 1.56410800 -0.13855500

C -2.35878900 -1.03800900 -1.23614500

C 1.67400200 -0.53471400 1.32571000

C 0.66268500 1.46669800 -0.82754900

Fe 0.75858400 -0.10977200 -0.09645800

H 4.60941800 -0.19541700 -2.11166000

H -2.91259800 -1.89259900 -1.64210300

C 3.85674500 0.23024200 -1.44397000

H 4.20535100 0.24603700 -0.41060500

C -2.44615400 0.75772900 0.60482700

H -2.32328500 -0.26521300 -2.01453000

Cl 2.43231900 -0.83668800 -1.52268200

**TS: Fe(CO)_2_(PH_2_CH_2_CH_2_CH_2_PH_2_) + CH_3_Cl**

***E*** = -3124.75

**H** = -3003.34

**G**  = -3042.90

***N*_imag_** = 1, -509.26 cm^–1^

O -4.80497400 3.32174600 1.92489100

H -8.20389900 -0.69945200 1.23068600

H -7.60999900 1.52288100 -2.15450500

H -7.30086300 -0.05601400 -1.46493600

O -4.87735100 -2.40763200 2.02474500

H -3.42818700 0.88168600 -0.29623200

H -8.46632000 1.04654300 2.37981000

H -4.64339800 -0.82824800 -0.60705500

C -7.17740300 1.02045500 -1.27850600

H -9.05466600 1.27453600 -0.23214000

H -7.84067700 2.49415900 0.16497600

C -5.69011500 1.36681000 -1.22680700

C -5.08397800 -1.26071800 2.05025400

C -5.05377200 2.18221600 1.98695700

Fe -5.42232700 0.46486600 2.01419900

H -5.21956000 1.16953900 -2.19835300

C -7.98509100 1.42680000 -0.04636600

Cl -5.68630300 0.50760600 4.35129600

H -3.42355000 -0.28160800 4.65904300

H -3.10155000 0.40300700 3.01941900

H -3.44907200 1.52207500 4.39661000

C -3.60606800 0.53667200 3.96774800

H -5.55139000 2.43348600 -1.01108200

P -7.49462500 0.51121800 1.47954600

P -4.74150900 0.43129900 0.04334300

**P: Fe(CO)_2_(PH_2_CH_2_CH_2_CH_2_PH_2_) + CH_3_Cl**

***E*** = -3173.71

**H**  = -3050.98

**G** = -3090.29

***N*_imag_** = 0

H 2.19648900 2.28541700 0.22340300

P -0.43748600 0.98633300 1.18306200

P -0.69785400 -1.31606000 -0.98268800

O 0.43165500 2.13666600 -2.17108600

H -0.27058400 0.63483300 2.54909100

H -3.87972600 -0.52632600 0.91428100

H -2.39874500 -1.23362400 1.52357500

O 1.85106100 -1.76925300 1.83862300

H -0.74739000 -1.48403300 -2.38666500

H -0.29552900 2.39097200 1.34914200

H -0.56762800 -2.68281800 -0.63160500

C -2.79072300 -0.53200400 0.77360100

H -2.68951900 1.27169500 1.98090600

H -2.57470000 1.55828200 0.24405400

C -2.49001100 -1.03996000 -0.63421100

C 1.42271700 -1.07892500 1.01975400

C 0.56056300 1.28075000 -1.40611800

Fe 0.80587000 0.01814100 -0.20812100

H 3.23970000 0.89564000 -0.18442000

Cl 2.14280900 -1.13461200 -1.70518400

C 2.38508700 1.22370100 0.41133400

H 2.61923200 1.10741900 1.47472000

C -2.26824500 0.87678700 1.04829800

H -2.84547900 -0.31136900 -1.37456400

H -3.02765000 -1.97554800 -0.82803600

**RC: Fe(CO)_2_(PH_2_CH_2_CH_2_CH_2_CH_2_PH_2_) + CH_3_Cl**

***E*** = -3530.87

**H** = -3390.13

**G**  = -3430.11

***N*_imag_** = 0

H -2.21659500 1.63584900 -1.00325300

P -0.03584600 -0.53328800 1.82883900

P -1.02825100 -0.43313400 -0.90842600

O 0.80554000 2.43738700 -1.50905000

H -0.88230900 -1.67936600 1.90231300

H -3.88222900 1.38205200 0.81957100

H -3.21914700 -0.16281700 1.28046400

C 0.83159900 1.44437700 -0.88562800

H -1.12402700 -0.47342500 -2.32781000

H 0.77417400 -0.92097600 2.93934500

H -1.62623900 -1.70253700 -0.66135100

C -2.94281400 0.81554700 0.86431100

H -2.56156300 2.21835600 2.45663200

H -1.31586800 2.19345700 1.22439700

C -2.47597800 0.65569700 -0.58373600

O 1.70894000 -2.48846800 -1.49937300

H 4.07252200 0.86557200 -0.98863600

C 1.39769600 -1.54095800 -0.88511900

C -1.97946700 1.54729600 1.81112000

H -3.29427400 0.25350000 -1.19732800

C -1.12603600 0.67305500 2.72389800

H -0.45239500 1.31743100 3.30373200

H -1.74534100 0.12552800 3.44628100

Fe 0.85358400 -0.09841800 -0.07860500

Cl 2.85027900 0.40034600 0.97261800

H 4.32652100 -0.81902300 -0.41197100

H 5.12879300 0.55394200 0.43885000

C 4.24842900 0.22910100 -0.12055900

**TS: Fe(CO)_2_(PH_2_CH_2_CH_2_CH_2_CH_2_PH_2_) + CH_3_Cl**

***E*** = -3501.47

**H**  = -3362.17

**G**  = -3401.58

***N*_imag_** = 1, -485.84 cm^–1^

H -1.64076800 1.45156500 -0.92473000

Fe 1.02635600 -0.86124800 -0.25443600

P -0.79401600 -0.77262100 -1.28242900

C -2.04021500 0.78734900 1.78632400

H 1.35037500 -0.79388500 2.80809800

H -3.70401200 0.87030800 0.47247900

H -3.08317700 -0.74718100 0.67362700

H -2.85852300 0.38511500 -1.61749300

P 0.68567500 -0.16110500 1.71459700

C 1.33207600 0.76545500 -0.84201600

C 2.49717700 -1.86661800 -2.15299900

C -2.76899800 0.29661700 0.53218700

H -2.81733100 0.96629200 2.54174600

H -1.59682000 1.77440200 1.59367800

C -2.09496100 0.45714500 -0.83145500

C -1.01012500 -0.13026700 2.44864700

H -0.88642900 0.17040000 3.49690000

H -1.37013000 -1.16711500 2.46105300

O 1.49174800 1.86116500 -1.20544600

Cl 3.31381400 -1.41002700 -0.25313800

O 0.23403700 -3.55807800 0.54375000

H -0.79434900 -0.53157200 -2.69357400

H 1.08403100 1.15869900 2.06963600

H -1.61085800 -1.93475200 -1.34576300

C 0.57725000 -2.49395900 0.21572500

H 3.49766200 -1.79849800 -2.57274100

H 1.80063000 -1.23049800 -2.68914400

H 2.15204900 -2.89009400 -2.04332200

**P: Fe(CO)_2_(PH_2_CH_2_CH_2_CH_2_CH_2_PH_2_) + CH_3_Cl**

***E*** = -3550.18

**H**  = -3409.01

**G** = -3449.60

***N*_imag_** = 0

H -1.90893300 1.99855100 -0.55650500

P 0.38556400 0.03534700 1.88851600

P -0.98445900 -0.16594700 -1.03416100

O 1.11391800 -3.02542200 -0.27042500

H 0.99139300 -0.92002900 2.73778700

H -3.95093700 1.37814200 0.76070600

H -3.46024600 -0.29754000 0.77932500

O 1.19145800 2.72002600 -0.77528100

H -1.05029100 0.02220400 -2.44285300

H 0.83375100 1.20385300 2.55188800

H -1.69945600 -1.39476400 -0.99803500

C -3.06540300 0.72812800 0.77362000

H -3.10611700 1.07266300 2.86397200

H -1.85824300 1.98586400 2.05576900

C -2.33440600 0.98743700 -0.54171800

C 1.04675300 -1.87439300 -0.21088900

Fe 0.94610000 -0.12075200 -0.19308300

C 1.08319500 1.59919700 -0.51702100

C -2.33345800 0.99544200 2.08750500

H -3.06200900 0.94385400 -1.36282100

C -1.33297600 -0.05903300 2.56133100

H -1.22301900 0.00544900 3.65164600

H -1.70904100 -1.06919800 2.35190400

H 1.55532900 0.54856100 -2.72780400

Cl 3.02702200 -0.01903200 0.81305000

H 1.48945100 -1.21310400 -2.59559500

H 2.87992400 -0.32633700 -1.91143800

C 1.80178900 -0.29891800 -2.08059700

**RC: Fe(CO)_2_(PH_2_CH_2_PH_2_) + C_2_H_6_**

***E*** = -2793.77

**H** = -2687.24

**G**  = -2724.39

***N*_imag_** = 0

Fe 0.41863300 0.14308500 -0.41702900

C -2.11984600 -0.74323600 0.91226600

P -0.48193300 -0.13656600 1.55019300

P -1.37360400 -0.82834300 -0.75420500

O -0.45827800 2.40154600 -2.02032100

H -0.17264400 -1.14160000 2.52184100

C -0.06490100 1.54926500 -1.32892900

O 1.78067500 -1.94474500 -1.90803600

H -2.36498500 -0.35487600 -1.66344900

H -0.85209500 0.83726100 2.53298300

H -1.43778100 -2.20470500 -1.11192600

C 1.28739700 -1.10450300 -1.27008300

H -2.87615100 0.04392400 0.98226000

H -2.49760100 -1.67773800 1.33952200

H 3.27384900 0.72862900 2.64478600

C 2.94657200 1.39416900 0.59613700

H 3.64188300 0.67934300 0.15276100

H 4.25068300 2.11504500 2.15945600

C 3.26274700 1.65097800 2.05582400

H 2.94167500 2.31212700 0.00338000

H 1.90916500 0.97625900 0.49759100

H 2.52597100 2.32768200 2.50093900

**TS: Fe(CO)_2_(PH_2_CH_2_PH_2_) + C_2_H_6_**

***E*** = -2741.02

**H**  = -2634.66

**G**  = -2670.18

***N*_imag_** = 1, -621.97 cm^–1^

C -2.21826900 -0.62175700 0.20963900

P -0.82485500 -0.42468000 1.42637700

P -0.99784500 -0.57832600 -1.17300100

O -0.65810400 2.77030900 -0.79704900

H -0.80263100 -1.72170200 2.02101100

Fe 0.60895000 0.23336800 -0.08977100

C -0.13299700 1.77354000 -0.48715900

O 2.01741700 -2.23810500 -0.75144000

H -1.70036900 -0.01564800 -2.28034900

H -1.46459500 0.18495300 2.55898900

H -0.98204400 -1.91706200 -1.64781100

C 1.48028100 -1.24306600 -0.46357300

H -2.80263200 0.30516500 0.19293300

H -2.88579200 -1.48493700 0.30277900

H 2.24487500 0.09487400 1.79865600

C 2.54768800 1.25960900 -0.57001900

H 3.51017700 0.75197000 -0.49289100

H 3.12560300 1.55214900 1.41470600

C 2.16006900 1.05950800 1.29650500

H 2.67591500 2.34003800 -0.48675100

H 2.15598700 1.05875700 -1.57210500

H 1.42353300 1.70261600 1.78008500

**P: Fe(CO)_2_(PH_2_CH_2_PH_2_) + C_2_H_6_**

***E*** = -2782.83

**H** = -2676.35

**G**  = -2711.05

***N*_imag_** = 0

C -2.30267900 -0.66786200 0.00122100

P -0.96952600 -0.45249900 1.26189100

P -0.97842900 -0.60325400 -1.29479900

O -0.22054600 2.98881400 -0.11112200

H -0.97140000 -1.71716000 1.92035400

Fe 0.58300600 0.20564000 -0.09297400

C 0.06497400 1.86367900 -0.10807300

O 2.42027300 -2.04831500 -0.12311600

H -1.60246900 -0.01348000 -2.44023900

H -1.56053200 0.27040200 2.33979000

H -0.95463600 -1.92912500 -1.80240800

C 1.65648700 -1.17673500 -0.11105800

H -2.90509400 0.24598100 -0.03769500

H -2.95238700 -1.54592000 0.05992300

H 2.29216400 0.12069800 1.94473900

C 1.57467400 0.84653600 -1.81575300

H 2.57592500 0.42036200 -1.87197300

H 2.79761600 1.34703100 0.75339200

C 1.94248200 0.92198800 1.28544100

H 1.67113200 1.93861800 -1.81207400

H 0.98617100 0.59118500 -2.70383700

H 1.50911100 1.70422600 1.92166100

**RC: Fe(CO)_2_(PH_2_CH_2_CH_2_PH_2_) + C_2_H_6_**

***E*** = -3183.69

**H**  = -3058.93

**G**  = -3094.74

***N*_imag_** = 0

Fe -0.13665900 0.04739700 -0.10293400

C -4.61928900 -0.21041700 1.29553600

H -5.31895600 0.61647700 1.12764400

H -4.80347400 -0.17822700 3.44078200

C -4.01547900 -0.12663800 2.68021200

H -5.17399600 -1.14355800 1.14464600

H -3.85795200 -0.16452800 0.50752400

H -3.31540000 -0.95242600 2.86688100

H -3.46795600 0.81291000 2.82762100

C -0.44011000 -1.54308900 -0.75654100

P 1.04521600 0.20024600 1.70035600

P 1.60688800 0.08675900 -1.18769800

O -0.58792000 -2.57605100 -1.28125200

H 0.88507800 1.18309700 2.72405800

H 3.99891500 0.20269800 -0.60230900

H 3.19061500 -1.24417000 0.03580900

O -0.81808100 2.48998800 -1.53695400

H 1.84608600 -0.84405400 -2.23578500

H 1.21523800 -0.87320100 2.63077900

H 1.94574100 1.26271500 -1.91559700

C 2.81138800 0.53216700 1.22362200

H 2.89802500 1.62016700 1.11364600

H 3.49950800 0.21424900 2.01366400

C 3.09035400 -0.16067000 -0.10633300

C -0.58215400 1.52880500 -0.91586900

**TS: Fe(CO)_2_(PH_2_CH_2_CH_2_PH_2_) + C_2_H_6_**

***E*** = -3135.80

**H**  = -3011.15

**G** = -3046.58

***N*_imag_** = 1, -668.25 cm^–1^

Fe -0.49852190 0.02354540 0.14958853

C -2.65484387 -0.13488998 -0.39013094

H -3.31755731 0.73070861 -0.40837881

H -3.41006926 -0.04029141 1.55229516

C -2.32022131 -0.02007574 1.48559817

H -3.23074460 -1.05631345 -0.29615320

H -2.14908025 -0.17255810 -1.36023261

H -1.96126458 -0.89140007 2.03365753

H -2.00398440 0.90092013 1.97641307

C -0.36214673 -1.72757660 0.11039488

P 1.05155607 0.18026394 1.56355859

P 0.88279130 0.02651225 -1.43492144

O -0.22601314 -2.88550355 0.07455960

H 1.17067451 1.16578874 2.59245862

H 3.35462149 0.22291530 -1.40039051

H 2.75762600 -1.24759849 -0.61197174

O -0.56538149 2.92865754 -0.17747366

H 0.97046182 -0.93167262 -2.49125857

H 1.45454905 -0.91209233 2.39069601

H 1.07837208 1.16744246 -2.26779129

C 2.64470813 0.51260069 0.64863877

H 2.70329450 1.60227281 0.53223577

H 3.51135864 0.19177309 1.23686972

C 2.59440429 -0.16719272 -0.71440723

C -0.56647899 1.77066792 -0.04023409

**P: Fe(CO)_2_(PH_2_CH_2_CH_2_PH_2_) + C_2_H_6_**

***E*** = -3179.40

**H**  = -3053.40

**G**  = -3090.51

***N*_imag_** = 0

Fe -0.47087480 0.01874398 0.17266292

C -2.01064532 -0.08704024 -1.23055539

H -1.96128385 0.73029872 -1.95785817

H -2.76812755 -0.14546230 1.46396802

C -1.74493134 -0.00454189 1.82326640

H -2.96490759 -0.02058911 -0.70076005

H -1.98819797 -1.02865457 -1.78866749

H -1.50651471 -0.82067292 2.51381396

H -1.70143410 0.93231844 2.38809105

C -0.67414150 -1.71062791 0.25365005

P 1.19102567 0.20254142 1.48096028

P 0.95498349 0.02151792 -1.40156081

O -0.85616773 -2.85383829 0.31122830

H 1.24995929 1.19113080 2.50510276

H 3.42771752 0.20971653 -1.49509457

H 2.86124150 -1.24150182 -0.64947825

O -1.18873543 2.82845064 0.10767727

H 0.94607899 -0.94190525 -2.45160498

H 1.58508626 -0.89046156 2.30840765

H 1.09297823 1.16028328 -2.24939331

C 2.77243940 0.54904213 0.56931856

H 2.81235750 1.63655121 0.42636991

H 3.65115507 0.25505001 1.15375992

C 2.69317809 -0.16398933 -0.77341104

C -0.87407269 1.71328194 0.13268980

**RC: Fe(CO)_2_(PH_2_CH_2_CH_2_CH_2_PH_2_) + C_2_H_6_**

***E*** = -3562.01

**H**  = -3417.29

**G**  = -3456.11

***N*_imag_** = 0

O -5.19570600 3.50237700 1.72524100

H -8.04624900 -0.50221700 1.90391500

H -7.70999200 0.79216500 -2.02023100

H -7.13248900 -0.51512300 -1.01090200

O -5.43245500 -2.32620200 1.89772300

H -3.33504800 1.18791900 -0.59078800

H -8.11560900 1.50691600 2.52995200

H -4.13716400 -0.75555200 -0.65998500

C -7.13299400 0.57980100 -1.10955300

H -8.95216600 0.97316500 0.00385600

H -7.79128900 2.30100600 0.03128400

C -5.70620700 1.08293500 -1.34027800

C -5.32194300 -1.16573800 1.91162500

C -5.18900000 2.33676400 1.79302600

Fe -5.20239900 0.58455800 1.87231200

H -5.35356400 0.77807700 -2.33377200

C -7.88221200 1.20791600 0.06820900

P -4.48052400 0.49873100 -0.08175500

H -2.93684600 0.40389600 2.94234800

H -4.69811500 0.67305000 3.57155600

H -3.34879400 1.66210800 4.11773200

C -3.56926900 0.63903100 3.80100100

H -5.68573200 2.18018500 -1.31836400

P -7.26911900 0.67628000 1.73317100

H -3.59613200 -1.37709900 4.60320800

H -2.35157600 -0.33130700 5.30090700

H -4.04122500 -0.13097500 5.77680700

C -3.38301700 -0.35451300 4.92953600

**TS: Fe(CO)_2_(PH_2_CH_2_CH_2_CH_2_PH_2_) + C_2_H_6_**

***E*** = -3516.74

**H**  = -3372.83

**G**  = -3410.99

***N*_imag_** = 1, -663.48 cm^–1^

O -5.10168200 3.39074700 1.87795000

H -7.98578800 -0.69038300 1.58900300

H -7.76317500 1.33730600 -1.94498000

H -7.30890200 -0.17828600 -1.19722600

O -5.28311500 -2.44796700 1.95558400

H -3.36533300 0.96966000 -0.56080000

H -8.16952300 1.11380000 2.65711000

H -4.49685200 -0.79497600 -0.73806800

C -7.20850700 0.91214300 -1.09715000

H -8.97501700 1.21039000 0.12098300

H -7.74017000 2.45298100 0.33449000

C -5.73983100 1.31039500 -1.25212500

C -5.22581300 -1.28376800 1.96761300

C -5.13683100 2.22359100 1.91142300

Fe -5.18003500 0.47077300 1.95441400

H -5.39849400 1.09796500 -2.27261800

C -7.89295600 1.37607000 0.18981000

P -4.61110600 0.45482000 -0.06790600

H -2.82545000 -0.17430000 3.89081800

H -2.90576800 0.16813600 2.18845100

H -2.90623500 1.51677100 3.33665500

C -3.27092700 0.50589500 3.16119900

H -5.61930100 2.38890000 -1.08988000

P -7.26535300 0.53088800 1.70635300

H -4.88197700 -0.56030400 4.57098100

H -4.32707300 1.09357200 4.91371900

H -5.86536300 0.85458700 4.14590200

C -4.84781700 0.45990200 4.19182700

**P: Fe(CO)_2_(PH_2_CH_2_CH_2_CH_2_PH_2_) + C_2_H_6_**

***E*** = -3550.90

**H**  = -3407.97

**G**  = -3442.65

***N*_imag_** = 0

O -4.76477900 3.30160700 2.35218200

H -7.98111400 -0.73078700 1.38260600

H -7.77094800 1.48023300 -2.12217900

H -7.32859500 -0.08551300 -1.51544600

O -4.90039400 -2.40235400 2.42369800

H -3.49752600 0.90172600 -0.46930000

H -8.23958300 1.08743000 2.39834300

H -4.64718100 -0.77103500 -0.67674500

C -7.25475600 0.98194200 -1.29981600

H -9.05777900 1.07556600 -0.07053700

H -7.94437600 2.40963900 0.20438300

C -5.82382100 1.37957100 -1.28686300

C -5.07840700 -1.25535100 2.23595300

C -4.99334600 2.16931800 2.18334700

Fe -5.21616600 0.45831700 1.97650200

H -5.37345500 1.20674300 -2.26975000

C -7.99362800 1.33394500 0.00295500

P -4.76418900 0.46704600 -0.09969700

H -2.89372900 0.50788000 3.25829900

H -2.64239900 -0.38614000 1.74723100

H -2.69282700 1.36808100 1.72211800

C -3.13793000 0.48391000 2.20298900

H -5.74667000 2.43520200 -1.05843600

P -7.30669200 0.52275000 1.49518400

H -6.41152500 -0.05714400 4.42695800

H -4.62093300 0.30538800 4.58769000

H -5.89779100 1.47201800 4.29924900

C -5.54726100 0.47724400 4.03680800

**RC: Fe(CO)_2_(PH_2_CH_2_CH_2_CH_2_CH_2_PH_2_) + C_2_H_6_**

***E*** = -3939.92

**H**  = -3776.76

**G**  = -3817.41

***N*_imag_** = 0

H -1.76207100 1.89688000 -0.63300100

H -2.83920300 1.04954600 2.72476300

H -1.62618100 1.93439000 1.84011000

C -2.04155400 0.92691500 1.97950900

C -2.10991400 0.85663500 -0.66031600

H -3.73143500 0.90736300 0.69011100

H -2.89292700 -0.62023300 0.72173600

H -2.88287600 0.80523800 -1.43891800

C -1.00140300 0.01261500 2.63143100

H -0.92275000 0.25801700 3.69922800

H -1.32499400 -1.03437800 2.57499600

C -2.72585700 0.46509600 0.68486200

O 1.38985700 2.55391800 -0.43038300

H 1.32401200 -0.71952200 2.99128300

O 0.22602000 -3.06857700 0.50518900

H -0.66362100 0.38111500 -2.59975900

H 1.12312300 1.31954700 2.50994600

H -1.41138500 -1.33271800 -1.63645300

C 0.56423100 -1.97728200 0.26623200

C 1.25641200 1.40240000 -0.29580800

Fe 1.00323200 -0.31431900 -0.06261900

P -0.69173900 -0.15952900 -1.27426500

H 2.41554900 -1.04930300 -1.99364100

H 3.75846000 -0.02132300 -1.48149200

C 3.16660500 -0.90137100 -1.21492500

C 4.02740800 -2.13593600 -1.01627000

P 0.72240100 0.06875600 1.96173400

H 3.42384800 -3.01443900 -0.76873400

H 4.58658200 -2.36434400 -1.93148700

H 4.75593400 -1.99480200 -0.20960200

H 2.73108500 -0.63612700 -0.18263400

**TS: Fe(CO)_2_(PH_2_CH_2_CH_2_CH_2_CH_2_PH_2_) + C_2_H_6_**

***E*** = -3893.47

**H**  = -3731.66

**G**  = -3770.45

***N*_imag_** = 1, -659.40 cm^–1^

H -1.88660300 1.98024000 -0.39336800

H -3.01464800 0.81945700 2.92681700

H -1.86842400 1.87210600 2.13883700

C -2.23529500 0.83605900 2.15280100

C -2.28350300 0.96090300 -0.48157100

H -3.90961500 1.03532800 0.86755400

H -3.18194800 -0.54821600 0.78656400

H -3.04810900 0.98824500 -1.26872900

C -1.13170800 -0.10136600 2.64672500

H -1.00464100 0.03769200 3.72820200

H -1.41936000 -1.14974900 2.49649800

C -2.93849500 0.52285000 0.82947500

O 1.31932600 2.57358100 -0.57169500

H 1.27920500 -0.58437200 2.92137700

O 0.27433500 -3.07953300 0.42219600

H -0.95231300 0.37978200 -2.48523000

H 0.83040800 1.40508900 2.42217300

H -1.68148400 -1.26551900 -1.40384500

C 0.55099900 -1.96910500 0.19045300

C 1.18092200 1.42695300 -0.39964000

Fe 0.92366800 -0.28579500 -0.12847700

P -0.91524700 -0.09607800 -1.13455000

P 0.54785400 0.10910900 1.90408900

H 4.02773600 -0.35195800 -0.59736000

H 3.05221500 -0.20759600 0.82643300

H 3.29593400 -1.81134500 0.10778100

C 3.13526900 -0.75730000 -0.11418500

H 2.72266200 0.12970100 -2.28449300

H 3.00461600 -1.61259300 -2.05973500

H 1.38663900 -1.03597800 -2.28536100

C 2.34096400 -0.77824500 -1.81964300

**P: Fe(CO)_2_(PH_2_CH_2_CH_2_CH_2_CH_2_PH_2_) + C_2_H_6_**

***E*** = -3934.58

**H**  = -3771.96

**G**  = -3811.20

***N*_imag_** = 0

H -2.00091000 2.01656900 -0.43215400

P 0.33527600 0.11231000 1.84080200

P -1.02840900 -0.09658900 -1.04661200

O 1.26104200 -2.98582000 -0.13250200

H 1.02975500 -0.70669100 2.77203300

H -4.06157400 1.24437500 0.80825900

H -3.49301400 -0.40548300 0.73698200

O 1.50449100 2.68315300 -0.75059100

H -1.10365800 0.19545700 -2.43734300

H 0.63896100 1.34761800 2.47784000

H -1.75046200 -1.32140000 -1.12147200

C -3.14640200 0.63629500 0.79187000

H -3.21706300 0.82760400 2.90182300

H -2.04324200 1.88923200 2.16505900

C -2.41081700 1.00009800 -0.49585700

C 1.10168500 -1.83739000 -0.13762600

Fe 0.92726800 -0.10492000 -0.19520500

C 1.24093900 1.57793900 -0.51688000

C -2.44031000 0.86490900 2.12599300

H -3.13344300 1.00621700 -1.32181000

C -1.35802700 -0.13257900 2.53964500

H -1.23368900 -0.09881000 3.62974300

H -1.66337900 -1.15878700 2.29551000

H 1.53025200 0.49979000 -2.74299700

C 2.85543600 -0.04816100 0.60161900

H 1.18046600 -1.22787400 -2.63291200

H 2.74263600 -0.60837500 -2.06635400

C 1.67276100 -0.39160800 -2.12360200

H 3.56756200 0.05208000 -0.22180600

H 3.09338000 -0.96271800 1.15437400

H 3.00689400 0.79981700 1.28067300

**RC: Fe(CO)_2_(PH_2_CH_2_CH_2_CH_2_CH_2_CH_2_PH_2_) + CH_4_**

***E*** = -3935.63

**H**  = -3771.14

**G**  = -3813.07

***N*_imag_** = 0

H 2.15926000 1.33248800 1.34212200

C 2.82833800 0.51628500 1.07956300

H -0.33757300 1.55768700 2.89107100

H 2.43810600 -0.26655400 0.35106800

H 3.12572200 -0.03934200 1.97123000

C -1.05870200 0.72957100 2.85936500

H -3.20645600 0.53465800 2.72782600

H -2.62811600 2.13357300 3.12214700

C -2.43383400 1.27176400 2.46983300

H 3.69842100 0.91837200 0.55591800

H -1.10292500 0.31264600 3.87334000

H -2.24546100 1.42778800 -1.69794400

P -0.24736000 -0.53623100 1.75813100

P -1.00324700 -0.60060800 -1.25740600

O 0.86645100 2.24900200 -1.73252000

H -1.20246400 -1.58608500 1.88426500

H -4.24800700 0.80872700 -0.07129900

H -3.16495000 -0.36768300 0.60574900

O 1.70284300 -2.64429400 -1.38218200

H -0.83432200 -0.75312200 -2.66108200

H 0.62051600 -1.05920300 2.76304200

H -1.62751100 -1.85789600 -1.01685500

C -3.18210900 0.60293300 0.09295800

H -3.24850700 2.57488000 0.95800200

H -1.62560300 2.02144500 0.62097400

C -2.51160300 0.45388000 -1.27051000

C 1.34059900 -1.67398800 -0.83694900

C 0.80801300 1.29908200 -1.04850600

Fe 0.71973300 -0.19641600 -0.16502500

C -2.59670900 1.69409700 1.00644700

H -3.18863600 -0.03604200 -1.98351100

**TS: Fe(CO)_2_(PH_2_CH_2_CH_2_CH_2_CH_2_CH_2_PH_2_) + CH_4_**

***E*** = -3927.24

**H**  = -3769.87

**G** = -3809.71

***N*_imag_** = 1, -771 cm^–1^

H -3.25197335 0.44677215 -1.68697144

P 0.09631449 -0.15705609 1.83387050

P -1.10436744 -0.53313757 -1.25123740

H 2.83292109 -1.38011004 1.14966871

C 0.67505549 -2.23679450 -0.28201071

O 0.65569290 -3.39535974 -0.39971295

H 2.99040846 0.38445148 0.84851978

C 1.02582749 1.03401968 -0.96440007

H -1.05757441 -0.43422536 -2.67304237

C 2.79358841 -0.59003471 0.39824924

O 1.25749121 2.00765177 -1.56430604

H 3.61307912 -0.78185510 -0.31028941

H 2.01713198 -0.77076090 -0.89176996

H -2.08853862 1.66026492 -1.17823740

H 0.40341295 2.03475252 2.69579117

C -0.51544030 1.46844189 2.49403760

H -0.99326774 1.29133527 3.46639278

H -0.86496449 -1.03110232 2.41846950

H -2.62969059 -0.28233298 1.00866151

H -3.37579817 2.67430657 0.86141901

C -2.83337883 1.84557696 1.33908082

C -1.40045320 2.31429621 1.58287276

H 1.07698834 -0.39140592 2.84595790

H -1.86184493 -1.73617487 -1.21007164

C -3.03737400 0.59231493 0.48661813

H -3.33096522 1.68725825 2.30697292

H -0.88270728 2.45996546 0.62771212

C -2.47552539 0.66226158 -0.94274719

H -4.11701087 0.40408766 0.42670151

Fe 0.73449638 -0.50097312 -0.19596279

H -1.45828169 3.31496978 2.03423287

**P: Fe(CO)_2_(PH_2_CH_2_CH_2_CH_2_CH_2_CH_2_PH_2_) + CH_4_**

***E*** = -3941.91

**H**  = -3779.42

**G**  = -3819.18

***N*_imag_** = 0

H -3.29551800 0.62397800 -1.60087800

P -0.02107900 -0.51579900 1.76442800

P -1.14421400 -0.35893900 -1.22608300

H 2.83507000 -1.34211100 1.14408900

C 1.03476800 -2.08333500 -0.54040500

O 1.28791300 -3.18333700 -0.80964700

H 2.84091900 0.42817700 1.22538000

C 1.15089400 1.26896200 -0.54907400

H -1.12846400 -0.15066700 -2.63346900

C 2.67540400 -0.42824900 0.56351500

O 1.50898100 2.33472900 -0.84242200

H 3.41698500 -0.39288700 -0.23853900

H 1.39867800 -0.39163400 -1.59027200

H -2.17799700 1.79853700 -0.93087700

H 0.27444600 1.43567300 3.06970100

C -0.64224300 0.94980700 2.71091300

H -1.17123600 0.58732300 3.60186100

H -1.04911200 -1.46024600 2.03948600

H -2.86444400 -0.42311700 0.97558000

H -3.38650600 2.57131200 1.24759500

C -2.91052400 1.64771800 1.60711800

C -1.46423200 1.98897200 1.95424900

H 0.86753200 -1.01404600 2.75875900

H -1.88350800 -1.57453500 -1.26936800

C -3.16751700 0.55093600 0.57369900

H -3.44911400 1.38057100 2.52799300

H -0.91805700 2.27980500 1.04976200

C -2.54557200 0.77192100 -0.81500300

H -4.25530200 0.47069900 0.45177100

Fe 0.75680500 -0.39575800 -0.24952500

H -1.48714700 2.89343200 2.57824400

**RC: Fe(CO)_2_(PH_2_CH_2_CH_2_CH_2_CH_2_CH_2_PH_2_) + CH_3_Cl**

***E*** = -3905.41

**H**  = -3745.59

**G**  = -3789.10

***N*_imag_** = 0

C -0.84655900 1.15142600 2.78371200

Cl 2.80976600 0.44655400 1.03982400

H 4.30789000 -0.87193300 -0.22335600

H -3.00030200 1.17378300 2.62112300

H 5.12862400 0.45381400 0.68384700

C 4.27510900 0.18089700 0.05862500

H -0.04272000 1.89941700 2.80658700

H -2.26786500 2.71413300 2.99357500

H 4.20534800 0.82134400 -0.82111100

H -0.94593500 0.76196300 3.80487500

H -1.90134100 1.87100600 -1.80987700

P -0.15722900 -0.21248400 1.71776400

P -0.85565000 -0.24830300 -1.29405000

O 1.22342900 2.46355200 -1.73281500

H -1.22669600 -1.14861500 1.82527300

H -3.96903400 1.49206800 -0.19664900

H -3.01890200 0.22659900 0.51806100

O 1.71776500 -2.53580400 -1.25028400

H -0.68065300 -0.45141300 -2.69158600

H 0.62375800 -0.81728500 2.74817000

H -1.59961100 -1.43689700 -1.04189400

C -2.93162500 1.18298900 -0.01332800

H -2.80425200 3.17070200 0.80845800

H -1.24353000 2.44221900 0.51256400

C -2.26261300 0.93720300 -1.36362000

C 1.39584400 -1.51665800 -0.76976500

C 1.08508700 1.51402100 -1.05730400

Fe 0.88183300 0.03128200 -0.17100400

C -2.24937800 2.22774100 0.88716600

H -2.97441300 0.49505400 -2.07329700

C -2.15318500 1.82314900 2.36168200

**TS: Fe(CO)_2_(PH_2_CH_2_CH_2_CH_2_CH_2_CH_2_PH_2_) + CH_3_Cl**

***E*** = -3873.49

**H**  = -3718.98

**G**  = -3761.40

***N*_imag_** = 1, -490 cm^–1^

H -3.58490400 -0.21329800 -1.66767100

O 0.22694000 -3.45949800 0.35085000

Fe 0.41440800 -0.57400800 -0.05167800

H -2.68168800 1.15217800 -1.03051400

H -0.41127900 1.98926600 2.74395000

H -2.60233200 2.72479600 2.21261300

H -2.72729700 -0.99082500 0.98561000

H -1.17723700 -0.42278600 -2.67217100

H 0.81885400 -0.19515100 2.97008100

C -1.16941600 1.21101200 2.58558600

H -1.55307300 0.94462500 3.57922100

H -4.28390300 1.63408200 1.02131200

H -4.35710500 -0.72582700 0.43222200

C -3.37605600 -0.24119600 0.51538500

H -3.93921400 0.61365700 2.40197400

C 0.47519700 1.11827300 -0.52274100

C 0.32473500 -2.31095900 0.18191100

H -1.77886100 -2.08346700 -1.51308900

C -2.27411300 1.80202500 1.71299100

C -3.52286300 0.96363600 1.44628700

H -1.83829400 2.13582000 0.76542000

C -2.86128400 0.07946700 -0.89730000

O 0.51594900 2.23385200 -0.85981700

P -1.27116500 -0.77428100 -1.28576600

Cl 2.75470600 -0.62952800 0.17754100

C 2.26345100 -1.05002100 -1.83696100

H 2.18669400 -2.13315800 -1.84180700

H 1.47564000 -0.55981500 -2.39911500

H 3.24605600 -0.69111700 -2.13380700

P -0.19975200 -0.24696500 1.96424100

H -0.92427000 -1.29474300 2.59938900

**P: Fe(CO)_2_(PH_2_CH_2_CH_2_CH_2_CH_2_CH_2_PH_2_) + CH_3_Cl**

***E*** = - 3924.29

**H**  = -3764.58

**G**  = -3806.60

***N*_imag_** = 0

H -3.38958600 -0.50724900 -1.65464400

P -0.35654500 -0.34504100 2.02765200

P -1.16835600 -0.99134700 -0.96657400

Cl 2.55199300 0.11830800 1.17398000

H -1.26626300 -1.37751400 2.38613400

H -2.49692100 2.79494200 2.12176800

H -3.07593100 -0.97390700 1.09763000

O 1.34974100 -3.27164600 0.44309200

H -1.05528200 -1.06195300 -2.38234600

H 0.52023800 -0.52536700 3.12547000

H -1.66603800 -2.30800500 -0.77085000

H -4.16051600 1.85639600 0.76792800

H -4.52776800 -0.45955800 0.30457000

C -3.48985800 -0.15858300 0.49420300

H -4.09691800 0.91560400 2.24084800

C 1.03875600 -2.16645700 0.32583800

Fe 0.60203700 -0.48384400 0.08048600

C 0.58355000 1.18763700 -0.46880200

C -2.23311800 1.83116700 1.66470800

C -3.54287700 1.13037800 1.31568700

H -1.69319400 2.09485600 0.74959400

C -2.76655200 -0.07799700 -0.86055700

H -2.57124500 0.95969700 -1.15517200

H -0.47472400 1.84919900 2.88841800

C -1.27177500 1.14126800 2.62706800

H -1.77006300 0.86779200 3.56633000

H 1.34117300 -0.04891700 -2.47578600

O 0.64723100 2.26370000 -0.88407600

C 1.66197200 -0.74520100 -1.69485200

H 1.55776200 -1.76064000 -2.09024700

H 2.70677600 -0.55671900 -1.44262400

**RC: Fe(CO)_2_(PH_2_CH_2_CH_2_CH_2_CH_2_CH_2_PH_2_) + C_2_H_6_**

***E*** = -4315.30

**H**  = -4132.78

**G**  = -4175.60

***N*_imag_** = 0

H 3.32051600 0.69751600 1.02890400

H 0.49106000 0.74147700 3.97866000

H -0.91756900 2.72908200 3.51527300

H 2.93621200 -0.35983900 -0.46468200

H 4.26201800 0.75803300 -0.45885400

C 3.73078300 0.08700000 0.22478200

C 4.60685100 -1.04156800 0.72796200

H 1.14909600 1.82203400 2.75209500

H 4.06073200 -1.71492000 1.39616100

H 5.00107400 -1.64148900 -0.09937400

H 5.46173600 -0.64020900 1.28553900

H -1.78504800 1.91315600 -1.21748000

P 0.65830000 -0.30266800 1.78539900

P -0.81964200 -0.29391700 -0.99154700

O 1.34948700 2.25250200 -1.90765300

H -0.38009700 -1.17957900 2.21497800

H -3.42204000 1.63193300 0.85785500

H -2.39527400 0.30906600 1.31629700

O 1.41182600 -2.79957600 -1.57295200

H -1.01393600 -0.47134800 -2.38992900

H 1.65445400 -0.92600700 2.59478800

H -1.56935000 -1.42854700 -0.56698700

C -2.39088300 1.26474600 0.77664600

H -1.95451400 3.23330600 1.53380400

H -0.56065000 2.42860500 0.85535600

C -2.09605500 0.99934500 -0.69783600

C 1.32613300 -1.76428300 -1.03322700

C 1.27756800 1.30230400 -1.22533500

Fe 1.14837200 -0.18487400 -0.33225400

C -1.44901700 2.26200900 1.47296900

H -2.99236500 0.62694500 -1.21191300

C -1.00385800 1.83995500 2.87653300

C 0.33709400 1.11115400 2.95721600

H -1.78572400 1.22456700 3.34228600

**TS: Fe(CO)_2_(PH_2_CH_2_CH_2_CH_2_CH_2_CH_2_PH_2_) + C_2_H_6_**

***E*** = -4264.69

**H**  = -4088.40

**G**  = -4130.05

***N*_imag_** = 1, -655 cm^–1^

H -3.55008700 -0.20873600 -1.66672800

P -0.15674200 -0.18546300 1.96824500

P -1.20543000 -0.67307600 -1.29698500

O 0.51123900 2.37909700 -0.68098200

H -0.83667100 -1.26434400 2.60018300

H -2.68595000 2.68378700 2.26379200

H -2.64710500 -1.00485800 0.96711800

O 0.31637700 -3.33408100 0.50207400

H -1.12925900 -0.27942200 -2.67382600

H 0.84582900 -0.08279100 2.99598900

H -1.66593200 -1.99145000 -1.57727000

H -4.32586100 1.54196600 1.05943200

H -4.29033800 -0.80955000 0.42266000

C -3.33394200 -0.27904300 0.51338400

H -3.92928100 0.51240200 2.41964900

H 3.00424200 -1.45666700 -1.58457400

Fe 0.47337800 -0.46981300 -0.03315000

C 0.48959400 1.24588600 -0.39778800

C -2.32194300 1.78588000 1.74386500

C -3.53387400 0.89805400 1.46871100

H -1.90887800 2.15655100 0.79995900

C -2.83685400 0.09553100 -0.89166800

H -2.70499000 1.17828200 -0.99838600

H -0.46113900 2.03657900 2.76490800

C -1.18630700 1.22901000 2.59860400

H -1.55146800 0.93242800 3.59079300

H 2.46629400 0.19779000 -1.96526900

H 1.36166900 -1.18354000 -2.06834300

C 2.18146400 -0.74287000 -1.49563000

C 0.39565700 -2.19122600 0.28068000

H 3.59115600 0.02858600 -0.10865000

H 2.41478300 0.10378600 1.16109100

H 3.01928300 -1.48209700 0.63137500

C 2.71854100 -0.49016500 0.29633700

**P: Fe(CO)_2_(PH_2_CH_2_CH_2_CH_2_CH_2_CH_2_PH_2_) + C_2_H_6_**

***E*** = -4309.79

**H**  = -4128.02

**G**  = -4170.25

***N*_imag_** = 0

H -3.50306800 -0.15560800 -1.69598700

P -0.34132400 -0.52390300 1.89522600

P -1.27756600 -0.79175500 -1.10896300

O 1.02230900 2.29207300 -0.56021100

H -1.27637400 -1.55316900 2.19727800

H -2.27197300 2.72867100 2.30732100

H -3.14355500 -0.87901700 0.99039800

O 1.29567500 -3.32985700 0.04249500

H -1.20098600 -0.71041500 -2.52798800

H 0.51532600 -0.81719200 2.99380100

H -1.85765200 -2.09042500 -1.06854700

H -4.04024200 2.03618600 0.93670400

H -4.58668700 -0.21140600 0.29845800

C -3.52632000 0.00979500 0.47518800

H -4.01558000 0.97140900 2.32512100

H 2.59860800 -0.70533100 -1.75153800

Fe 0.56905200 -0.52629500 -0.04552200

C 0.78230700 1.17928300 -0.33355600

C -2.10000600 1.78509100 1.77112900

C -3.46884600 1.22183500 1.40463300

H -1.55591700 2.07041900 0.86444100

C -2.83788300 0.17367800 -0.88919400

H -2.60348300 1.22462100 -1.09617600

H -0.35658000 1.57141800 2.99196100

C -1.18692700 0.93889000 2.65196300

H -1.70622900 0.59659900 3.55670400

H 1.26970200 0.16437300 -2.54930200

H 1.22344300 -1.59662600 -2.42823100

C 1.51577000 -0.68224900 -1.89995200

C 0.97100800 -2.21700800 0.02357100

H 3.18939400 -0.14324300 0.18529000

H 2.39273800 0.60675300 1.58084800

H 2.64189000 -1.14227900 1.54669300

C 2.39745300 -0.27316500 0.92735200

**RC: Fe(CO)_2_(PH_2_CH_2_CH_2_CH_2_CH_2_CH_2_CH_2_PH_2_) + CH_4_**

***E*** = -4309.84

**H**  = -4125.95

**G**  = -4169.91

***N*_imag_** = 0

H 2.48204100 1.71574000 1.27231300

C 3.14724500 0.97751900 0.83082700

C -3.74862400 1.18928500 1.11589800

H 2.71282600 0.32850100 0.00017000

H 3.54432800 0.30161100 1.58901100

H -3.45273500 2.81323500 2.46731700

H -3.04562600 3.21265100 0.82358000

O 0.92592100 2.63645300 -1.81091400

Fe 0.94216400 0.19665400 -0.22664800

H 3.95278700 1.49474400 0.30241700

H -2.14412300 1.68927700 -1.03515400

P 0.12361000 -0.10610300 1.77563200

P -0.78279400 -0.26068800 -1.31484000

H -3.76436700 -0.64109600 0.00368800

H -0.59617400 -1.30655700 2.03552500

H -2.29448600 -0.34992800 0.87160900

H -4.65104200 1.55003300 0.60273000

C 0.92093500 1.69206600 -1.11716000

H -0.66895900 -0.16267700 -2.72832000

H 1.08371100 -0.36117600 2.80013900

H -1.24996900 -1.60370900 -1.26903100

C -3.04427900 0.14998900 0.24863100

H -4.12060600 0.65007600 1.99788300

H -0.89647500 2.24324100 0.91019700

C -2.37737900 0.62062100 -1.04701600

C 1.56370500 -1.30641100 -0.83651100

C -2.94795700 2.41750300 1.57516800

O 1.94725700 -2.30064200 -1.31972000

C -1.44741400 2.28318600 1.85931400

H -3.01334700 0.44182000 -1.92305500

C -0.95329400 1.11618400 2.72183000

H -0.32249200 1.48643900 3.53710700

H -1.76893100 0.56415800 3.19971000

H -1.13144200 3.22582100 2.32378300

**TS: Fe(CO)_2_(PH_2_CH_2_CH_2_CH_2_CH_2_CH_2_CH_2_PH_2_) + CH_4_**

***E*** = -4295.30

**H**  = -4116.07

**G**  = -4154.90

***N*_imag_** = 1, -819 cm^–1^

H -1.75693300 1.77470800 -0.99522200

P 0.10787900 -0.23393600 1.78220800

P -1.13043600 -0.58746200 -1.05785300

H 2.89995900 -1.27264100 1.13825500

H -0.78677700 -1.21714500 2.30513100

H -2.58455000 0.01002900 1.16635300

H -4.17708800 2.54919700 0.57321000

C 1.04169800 1.03691100 -1.03521400

H -1.28183000 -0.75553200 -2.46690300

H 1.04118300 -0.34179100 2.85821700

H -1.92710200 -1.71073100 -0.68730800

C -3.07290500 0.69391300 0.45160100

H -3.82860200 1.78749800 2.13867100

H -0.36283200 2.23166500 0.56136500

C -2.28596200 0.81542800 -0.86130900

C 0.87582600 -2.22158100 -0.46628300

Fe 0.82448400 -0.49587600 -0.23433900

O 0.92806200 -3.36685500 -0.69302100

C -0.78322000 2.47044100 1.53552400

H -3.01380600 0.77258500 -1.69979300

C -0.63794900 1.29494200 2.50345600

H 0.00807500 1.62175500 3.34762500

H -1.60488400 1.03284300 2.97971700

H 2.95778800 0.50980000 0.87431100

H 3.67392200 -0.59828700 -0.29524500

H 2.06737600 -0.66102900 -0.91559700

O 1.23257100 2.00998100 -1.65485200

C 2.82863400 -0.46873500 0.40183700

C -3.38170100 2.02807400 1.14925300

H -4.04158300 0.19438200 0.22799600

H -0.14811200 3.30559800 1.90610700

C -2.21545000 3.02483000 1.37304600

H -2.45773400 3.63224300 2.27267200

H -2.19997100 3.73585400 0.51836900

**P: Fe(CO)_2_(PH_2_CH_2_CH_2_CH_2_CH_2_CH_2_CH_2_PH_2_) + CH_4_**

***E*** = -4313.11

**H**  = -4132.64

**G**  = -4172.80

***N*_imag_** = 0

H -2.11086000 1.96930900 -0.86638600

P 0.18618700 -0.16409000 1.76247400

P -0.78818200 0.06551800 -1.48361800

H 2.99243800 -1.16674600 1.13894200

H -0.75882600 -1.17915600 2.07997900

H -2.16666300 -0.31771800 0.72654200

H -4.52651100 1.56735800 0.97617900

C 1.46601900 1.56473300 -0.50994300

H -0.59467000 0.65597600 -2.76158800

H 1.12081400 -0.57421100 2.75450500

H -1.33948500 -1.15205800 -1.96800700

C -2.95043200 0.28025100 0.24902500

H -3.76743000 0.56465600 2.18274600

H -0.77309800 2.34578400 0.86105600

C -2.36229200 0.91457200 -1.01322100

C 1.14379100 -1.80210300 -0.48083900

Fe 0.97958100 -0.08785600 -0.26481300

O 1.32454400 -2.92863100 -0.68874800

C -1.20955700 2.36978300 1.86456900

H -3.05467100 0.87211200 -1.86234500

C -0.52610300 1.28177300 2.69616700

H 0.35341300 1.71799900 3.18475000

H -1.16168700 0.89777200 3.50270000

H 3.09113100 0.59766000 1.23829800

H 3.63669900 -0.23830400 -0.22961700

H 1.67770500 -0.14680800 -1.57502900

O 1.88663200 2.61921300 -0.75437300

C 2.88654200 -0.24112500 0.56494700

C -3.54765800 1.19933600 1.31337600

H -3.71347900 -0.45301000 -0.04181300

H -0.90415700 3.34168800 2.27456800

C -2.73811400 2.42247600 1.76864600

H -3.14270300 2.73096500 2.74284200

H -2.96297800 3.26351300 1.09851600

**RC: Fe(CO)_2_(PH_2_CH_2_CH_2_CH_2_CH_2_CH_2_CH_2_PH_2_) + CH_3_Cl**

***E*** = -4278.52

**H**  = -4100.27

**G**  = -4146.02

***N*_imag_** = 0

H -3.26852300 2.98317100 2.34471400

Cl 3.00805900 0.53044600 1.10523100

H 4.53714600 -0.55469900 -0.33036900

H -2.71082800 3.38483000 0.74661600

H 5.30994400 0.70823000 0.70058100

C 4.45503000 0.45788200 0.06764200

C -2.73726600 2.56559600 1.47811900

O 1.35025200 2.55961200 -1.58510700

H 4.32823100 1.18649900 -0.73453900

C -3.61097000 1.42852800 0.92588400

H -1.82350900 1.82920300 -1.09814800

P 0.05489400 -0.23730800 1.80398300

P -0.60814100 -0.22358000 -1.31272200

H -3.71774900 -0.36177900 -0.24428000

H -0.82958500 -1.34858900 1.92690400

H -2.29595300 -0.23605400 0.73546100

H -4.43836100 1.88536600 0.36482300

C 1.22100700 1.59161800 -0.93487400

H -0.38277300 -0.12185200 -2.71304000

H 0.87319400 -0.65957800 2.89456600

H -1.19278500 -1.52168600 -1.32970600

C -2.94582300 0.34963200 0.07636700

H -4.09162400 0.90395700 1.76299100

H -0.66316500 2.23312100 0.95436500

C -2.14233400 0.78412300 -1.15362100

C 1.65055300 -1.44594500 -0.69863100

Fe 1.07404200 0.07573900 -0.09376500

O 2.02454500 -2.44797700 -1.17681700

C -1.27791200 2.29506600 1.86210500

H -2.72183500 0.67480700 -2.07882700

C -0.93723400 1.07285700 2.72191500

H -0.29695700 1.37081400 3.55799000

H -1.81819300 0.60149400 3.17031500

H -0.92114400 3.19696900 2.37557400

**TS: Fe(CO)_2_(PH_2_CH_2_CH_2_CH_2_CH_2_CH_2_CH_2_PH_2_) + CH_3_Cl**

***E*** = -4244.68

**H** = -4072.31

**G**  = -4116.43

***N*_imag_** = 1, -499 cm^–1^

C -3.44332500 1.63326900 2.07191800

O 0.44666300 2.36273500 -0.94630300

P -1.33621700 -0.62424700 -1.21322400

Cl 2.76520800 -0.45366500 0.06572400

C -3.92119600 0.18738100 1.86793100

H -3.81375700 1.95632000 3.05529800

C 2.17931300 -0.92461000 -1.91954300

H 2.21879600 -2.00914900 -1.88302500

H 1.31798500 -0.54465400 -2.45844500

H 3.10119500 -0.47256500 -2.27669000

H -2.95074800 1.02207400 -0.55345500

P 0.00574700 -0.12027300 2.04160300

H -3.96982600 2.27800100 1.35399800

H -3.80433100 -1.60657100 0.69485900

H -0.61514300 -1.19149400 2.74438100

H -2.26416900 -0.98607300 1.20825800

H -5.00103100 0.23430500 1.66776700

C 0.43508500 1.26208900 -0.56260500

H -1.38394900 -0.03253800 -2.51311600

H 1.14888200 -0.07820800 2.90142600

H -1.69026600 -1.93293500 -1.64719100

C -3.23587200 -0.67410700 0.80670500

H -3.83107400 -0.36217300 2.81514900

H -1.64847500 1.96274100 0.90773500

C -2.98658500 -0.07103000 -0.57706700

C 0.33844600 -2.15988600 0.16451400

Fe 0.41436000 -0.42045100 -0.05266800

O 0.25155600 -3.31160300 0.31550700

C -1.96360500 2.01946900 1.95306300

H -3.75530100 -0.34911200 -1.30848700

C -0.89609100 1.32501500 2.80076300

H -0.09030000 2.04567700 2.98866300

H -1.27037500 1.02001000 3.78559200

H -1.92154700 3.09034400 2.19529700

**P: Fe(CO)_2_(PH_2_CH_2_CH_2_CH_2_CH_2_CH_2_CH_2_PH_2_) + CH_3_Cl**

***E*** = -4295.04

**H**  = -4116.86

**G** = -4160.72

***N*_imag_** = 0

C -3.42861100 1.67202700 2.07849800

O 0.66472100 2.36705400 -1.02004300

P -1.36191000 -0.71268700 -1.06704000

Cl 2.71829400 -0.07485000 0.52991200

C -3.90463300 0.21292900 2.00007100

H -3.75258900 2.05474500 3.05640500

C 1.20148600 -0.86367600 -2.02134500

H 0.76724300 -1.78240000 -2.43434500

H 1.02118900 -0.05204800 -2.73263000

H 2.27718100 -1.00180300 -1.89928400

H -3.04796100 0.88546900 -0.48324000

P -0.00511400 -0.07375900 1.99699300

H -4.00008300 2.26481600 1.35096700

H -3.89979800 -1.63584600 0.91509100

H -0.70488000 -1.11380200 2.66559500

H -2.32583100 -1.09096300 1.38273300

H -4.99132400 0.24895300 1.84272000

C 0.58416200 1.28422200 -0.62790200

H -1.45420600 -0.18725600 -2.38170700

H 1.12336500 -0.08428200 2.85282900

H -1.63859500 -2.06427900 -1.40047100

C -3.27685400 -0.73457900 0.97516600

H -3.77792300 -0.26402200 2.98119900

H -1.69829400 1.97734300 0.80726200

C -3.02215700 -0.20700400 -0.43874700

C 0.74180800 -2.11538900 0.19574000

Fe 0.51737400 -0.39845900 -0.10914500

O 0.90548500 -3.24472400 0.36677400

C -1.96356000 2.06946100 1.86434900

H -3.76913300 -0.56138300 -1.15968800

C -0.84996800 1.42394500 2.68754900

H -0.02622800 2.14423600 2.77264900

H -1.16179300 1.19349700 3.71376800

H -1.92257700 3.14926400 2.06085200

**RC: Fe(CO)_2_(PH_2_CH_2_CH_2_CH_2_CH_2_CH_2_CH_2_PH_2_) + C_2_H_6_**

***E*** = -4688.87

**H** = -4487.98

**G** = -4532.53

***N*_imag_** = 0

H 3.63978401 -0.07638630 1.34903673

H 3.63588201 -2.52832330 0.68916273

H 4.64935301 -2.15296630 -0.70754627

H 3.00374001 -0.27267530 -0.28991127

H 4.65696701 0.29715170 -0.04772227

C 3.89761901 -0.39756130 0.33247873

C 4.39487401 -1.83347530 0.30993573

H 5.29278701 -1.95854330 0.92681173

H -2.10527400 1.96563300 -1.22069800

P -0.22386300 -0.25192300 1.76037400

P -1.55395300 -0.34027000 -1.49652100

H -4.32256100 0.35889400 0.07998400

H -0.78712600 -1.25926000 2.59603600

H -2.75701800 0.21541500 0.81991700

H -4.41193800 2.75918000 0.53376800

C 0.76284400 0.80973000 -1.32306700

H -1.43829200 -0.23948700 -2.90978300

H 1.04439200 -0.26770400 2.41979800

H -2.46422300 -1.43393800 -1.46999600

C -3.37232600 0.89144300 0.21433600

H -4.11677600 1.81608200 1.96688100

H -0.65835400 2.13783500 0.65620800

C -2.70648900 1.05359400 -1.15385200

C 0.26353700 -2.18199900 -0.91550800

Fe 0.18773500 -0.52799400 -0.38663900

O 0.34076000 -3.25979900 -1.36346700

C -1.08720600 2.44455100 1.61851500

H -3.44175800 1.09403900 -1.96803900

C -1.01782400 1.24012500 2.56244400

H -0.46730200 1.46453400 3.48246600

H -2.01215200 0.91080400 2.88101600

H -0.41746500 3.23263600 1.98448800

C -2.46162100 3.07517100 1.37134300

H -2.75732800 3.67421500 2.24384600

O 1.18438100 1.63386600 -2.04273900

H -2.32695100 3.80505400 0.56144200

C -3.64429700 2.15213300 1.03366100

**TS: Fe(CO)_2_(PH_2_CH_2_CH_2_CH_2_CH_2_CH_2_CH_2_PH_2_) + C_2_H_6_**

***E*** = -4635.84

**H** = -4441.75

**G**  = -4485.09

***N*_imag_** = 1, -652 cm^–1^

C -3.34802400 1.56230300 2.12209000

O 0.71868200 2.44772700 -0.68407200

P -1.23685800 -0.47311900 -1.25237200

H 3.00294700 -1.57031000 0.46510700

C -3.80460300 0.11277100 1.89272800

H -3.68434700 1.85353400 3.12771500

H 2.82649600 -1.59867700 -1.76897200

C 2.08470200 -0.80563200 -1.64622900

H 1.19588300 -1.16958700 -2.16847300

H 3.66685300 -0.16893300 -0.39768500

H -2.96540700 1.02819800 -0.53137600

P 0.14096900 -0.12396600 2.02052200

H -3.91312600 2.21617300 1.44286100

H -3.65761200 -1.65401000 0.68203900

H -0.45849300 -1.20240200 2.73074900

H -2.12651800 -0.99113800 1.17164300

H -4.88830000 0.14339700 1.71059500

C 0.63243400 1.31771900 -0.40535300

H -1.30596200 0.28053900 -2.46384600

H 1.27636800 -0.04830300 2.90005000

H -1.54780500 -1.71928500 -1.87327000

C -3.11755100 -0.70548000 0.79899300

H -3.68851500 -0.45765800 2.82473900

H -1.59706900 1.90785000 0.89972700

C -2.91903700 -0.06416500 -0.57528300

C 0.33190500 -2.11539500 0.23601500

Fe 0.51893300 -0.39923200 -0.05784600

O 0.19199300 -3.25522700 0.44087600

C -1.88120300 1.97522900 1.95337300

H -3.67451700 -0.38126000 -1.30406800

C -0.77764300 1.31183400 2.77931000

H 0.01535500 2.05178200 2.94594700

H -1.12199500 1.00380000 3.77420200

H -1.85097700 3.04985000 2.18025500

H 2.42975600 0.09915600 -2.14518600

C 2.78089600 -0.56694300 0.10333100

H 2.61739500 0.09827600 0.95385900

**P: Fe(CO)_2_(PH_2_CH_2_CH_2_CH_2_CH_2_CH_2_CH_2_PH_2_) + C_2_H_6_**

***E*** = -4680.75

**H** = -4480.47

**G** = -4524.93

***N*_imag_** = 0

H -2.95332000 0.91060900 -0.65324700

C -3.02905900 -0.16863200 -0.49170200

H 2.68789600 -1.44691700 1.46014900

H 0.58432400 -2.26146900 -2.12941600

C 2.46124400 -0.61832000 0.78172300

H -3.75064900 2.56330500 0.94781100

H 3.14381100 -0.67213700 -0.07007600

H 2.66227800 0.31363400 1.32200300

H -4.11455300 -1.33854600 0.96040000

C 1.20443100 -1.45730100 -1.71282000

C 0.56772700 -2.38829700 0.56530800

C -1.78693000 2.17310000 1.61923100

H -2.50779500 -0.91505400 1.44561000

H -5.01983300 0.75586900 1.61640700

H -3.92620300 0.23934700 2.87038900

C -3.30132400 2.00088600 1.77793400

H -1.48015200 1.89970300 0.60495900

C -3.39761300 -0.50785700 0.95451100

H -3.77323200 -0.53461500 -1.20894300

C -3.95315500 0.61165300 1.83730400

H -3.62168500 2.54140000 2.68007500

C -0.82854200 1.49495300 2.59736100

H 0.05933600 2.13051300 2.70594200

H -1.25235100 1.38993800 3.60364700

H -1.60456600 3.25443300 1.67923700

P -0.10358300 -0.14145700 2.09810600

P -1.38521400 -0.88366300 -0.95128300

O 1.26250800 1.88709500 -0.93557100

H -0.93193200 -1.03687700 2.82869900

O 0.66634400 -3.50713500 0.85233800

H -1.35083400 -0.46842300 -2.30910300

H 0.94660400 -0.15493000 3.05899800

H -1.79748600 -2.21810200 -1.21238500

H 2.20388800 -1.86728700 -1.54798100

Fe 0.49085500 -0.71171700 0.10426100

C 0.91019200 0.86872800 -0.50625100

H 1.27352500 -0.66790900 -2.46756400

**RC: Fe(CO)_2_BN(CH_3_)_2_(PH_2_CH_2_CH_2_CH_2_PH_2_) + CH_4_**

***E*** = -4069.82

**H**  = -3857.84

**G** = -3898.47

***N*_imag_** = 0

C -1.82380492 0.40542232 -1.32533406

H -2.32769788 3.82695331 -4.66588084

Fe -0.39599615 0.41993049 -2.41415086

H 2.39465508 2.01363581 -6.03149248

B 0.97781934 0.49941552 -3.60462638

H 3.63271048 1.83593940 -4.75966157

O -2.78160829 0.40588247 -0.67895134

H 2.31710711 -0.54651001 -6.30262284

C 2.55307332 1.83996370 -4.95907088

H -3.36680586 2.81712996 -3.64943744

P -1.62649519 -0.38842931 -4.12248914

P -1.04469245 2.41094000 -3.22414512

C 2.47774710 -0.61012425 -5.21839715

H -0.77678162 -0.68989330 -5.23571957

H -3.50211813 2.22671683 -6.08889752

H -1.78955602 1.86318967 -6.11382747

H 2.09720375 2.66267767 -4.40428290

H -1.03361860 3.58049248 -2.40730093

H -2.16136146 -1.71356256 -4.02619814

H 0.02560474 2.78823529 -4.08943620

C -2.67951108 1.84474604 -5.46870647

H -3.21250150 -0.19165497 -5.97137548

H -3.88639656 0.36995620 -4.43069311

C -2.48262191 2.80333784 -4.29878055

H 3.55418407 -0.71388524 -5.02808453

H 1.96904701 -1.50117601 -4.84407109

N 1.95867976 0.57628160 -4.56179055

C -2.99992977 0.40395743 -5.07621104

H 0.87454630 0.33798438 -1.04510956

C 1.06004921 -0.73148981 -0.73249992

H 0.42412291 -1.47542275 -1.21721192

H 0.88116572 -0.75133357 0.34374116

H 2.10649512 -0.93715024 -0.96201101

**TS: Fe(CO)_2_BN(CH_3_)_2_(PH_2_CH_2_CH_2_CH_2_PH_2_) + CH_4_**

***E*** = -4056.85

**H**  = -3889.87

**G**  = -3932.50

***N*_imag_** = 1, -751.53 cm^–1^

Fe 0.39138500 -0.26384800 0.12124600

P 0.18824200 0.88632900 -1.60417400

B -1.37021600 0.03166600 0.13282900

H 2.46930700 -2.61851000 -2.90940000

O 2.48042100 1.53292800 1.06555700

C 2.25289900 -1.65041100 -2.44189600

P 1.19982000 -1.91995400 -0.94808100

C 1.68554800 0.75739600 0.69523500

N -2.72635000 0.35135200 0.14119400

H -3.24082500 -1.42521100 -0.82270700

H -3.94017500 1.37673700 1.52984200

C -3.23035700 1.58145000 0.71482300

H -2.40096000 2.16627300 1.11980600

H -3.74885300 2.18978400 -0.04153000

C -3.72506200 -0.53119900 -0.42080100

H -4.45250300 -0.84533900 0.34243600

H -4.27962100 -0.03953600 -1.23469600

C 0.40247800 -1.46040900 1.94442200

H -0.46472000 -0.46347900 1.36226600

H 0.13134900 -1.01280700 2.90991100

H 1.47163400 -1.66720100 1.98326800

H -0.15970300 -2.38998000 1.82631300

C 1.43820500 0.72948000 -2.95841400

H -0.97720000 0.82476400 -2.42220700

H 2.27609000 -0.67007500 -4.35037300

H 0.66862100 -1.10311400 -3.80822500

H 3.20559500 -1.24115300 -2.08092700

H 2.04998500 -2.85165400 -0.27455800

H 0.20251400 2.30903500 -1.50089100

H 0.34849200 -2.92931500 -1.49543600

C 1.63071800 -0.69844000 -3.46147500

H 1.12388500 1.38606800 -3.77981400

H 2.38280000 1.12576000 -2.56344100

**P: Fe(CO)_2_BN(CH_3_)_2_(PH_2_CH_2_CH_2_CH_2_PH_2_) + CH_4_**

***E*** = -4079.16

**H**  = -3903.33

**G**  = -3944.67

***N*_imag_** = 0

Fe 0.08688300 0.39840100 1.33716700

H -1.67132200 -2.12710000 1.48240800

B 0.64946900 0.05326000 -0.33405300

H -1.39829200 2.82969800 0.22055700

O 0.58288500 -0.16478300 4.15794600

H -3.99487800 -0.94687200 1.85552700

H -2.94544300 -0.39646300 3.16859700

C 0.35763000 0.08725700 3.04127300

N 1.11475800 -0.20324600 -1.62020400

H -0.05154100 1.34305200 -2.39825300

H 3.01118500 -0.78504800 -2.33919600

C 2.08759700 -1.22564700 -1.93438900

H 2.34531200 -1.78892800 -1.03390700

H 1.69239400 -1.93058400 -2.68075100

C 0.68376800 0.60839600 -2.73649900

H 1.52910800 1.14959400 -3.18734600

H 0.21964500 -0.00954900 -3.51944000

C 1.79192500 1.59476600 1.25549000

H 1.00991700 -0.75820300 0.71438800

H 2.68476400 0.96630700 1.34400200

H 1.78644100 2.26976700 2.11914300

H 1.90269600 2.21531700 0.35831500

C -3.62264200 1.18685100 1.85195600

P -1.67892000 -0.73705000 1.15437500

H -3.04870300 3.25084700 2.17553100

C -2.62374000 2.24789700 2.30676300

P -1.00442500 2.17892800 1.42753100

C -3.18321800 -0.25337500 2.10664600

H -2.30875500 -0.91779200 -0.11254700

H -4.57418100 1.35797800 2.37440100

H -3.84391900 1.32142000 0.78297200

H -2.40264300 2.12558100 3.37516800

H -0.40004800 3.32981800 2.01461800

**RC: Fe(CO)_2_BN(CH_3_)_2_(PH_2_CH_2_CH_2_CH_2_PH_2_) + CH_3_Cl**

***E*** = -4033.44

**H**  = -3860.01

**G** = -3905.38

***N*_imag_** = 0

C -1.87025100 -0.40971800 -2.05277400

H -2.83655400 3.81818400 -3.87180800

Fe -0.52086600 0.35539100 -2.87089800

H 2.77392500 1.71246300 -6.11550000

B 0.94482600 0.24727700 -3.93546300

H 3.82227000 1.44828000 -4.70088600

O -2.78736500 -0.96361400 -1.57436300

H 2.65256600 -0.85600800 -6.43693600

C 2.77558800 1.49722500 -5.03598800

H -3.52478600 2.35482800 -3.15799800

P -1.31343700 -0.23584700 -4.67062600

P -1.13044000 2.35834300 -2.92155900

C 2.64773300 -0.92672800 -5.33849300

H -0.61405400 -0.12213500 -5.90751600

H -3.72557800 2.48990600 -5.67587300

H -1.98057100 2.44047700 -5.81337900

H 2.28789600 2.32531500 -4.51634400

H -1.39099600 3.14808900 -1.75361400

H -1.71533500 -1.59173800 -4.88397400

H -0.34113600 3.37778400 -3.53648900

C -2.82039500 2.10045900 -5.18901100

H -3.13289600 0.21196500 -6.21277200

H -3.67389900 0.21991200 -4.52828400

C -2.71757700 2.73019100 -3.80002300

H 3.68539900 -1.08064200 -5.00626200

H 2.05986000 -1.80233400 -5.05144500

N 2.07793500 0.26178600 -4.74391300

C -2.88763100 0.57536100 -5.20624200

H 0.12735100 -1.76089700 -0.13618000

Cl 0.85452300 0.30288900 -1.03100700

C 1.09717300 -1.36997500 -0.44783400

H 1.79769400 -1.33134800 0.39039400

H 1.49747000 -1.95370500 -1.27841000

**TS: Fe(CO)_2_BN(CH_3_)_2_(PH_2_CH_2_CH_2_CH_2_PH_2_) + CH_3_Cl**

***E*** = -4004.92

**H**  = -3832.02

**G** = -3874.80

***N*_imag_** = 1, -524.34 cm^–1^

Fe 0.42595041 -0.00315374 0.30439623

H 0.74582253 -2.19503347 1.47043256

B -1.33051782 -0.34487840 -0.04204490

H -0.53766078 -1.92906387 2.71554368

O 3.35002415 0.08063685 0.62190466

Cl -0.10667674 0.47522116 2.55727902

H 1.19651368 -1.50662859 3.07549019

C 2.19731532 0.04095563 0.54832710

N -2.64955786 -0.57127444 -0.34486228

H -2.41063159 -2.65580880 -0.37098959

H -4.40346086 0.44152789 0.23472895

C -3.59140708 0.52638100 -0.49969695

H -3.08353271 1.48082730 -0.34426759

H -4.02673805 0.52165104 -1.50797863

C -3.19544148 -1.90818079 -0.50565882

H -3.98787504 -2.09552834 0.23149819

H -3.61774788 -2.03073868 -1.51208872

C 0.42180884 -1.60641671 2.32090837

C 1.26052085 1.74771584 -2.44493757

H -0.88475985 2.25008485 -1.26935987

H 1.35427623 0.71206556 -4.31424560

H -0.16670855 0.49144341 -3.47731654

H 2.50128583 -0.75655116 -2.59355516

H 1.21407623 -2.68061093 -1.17382807

H 0.83754429 3.03155376 -0.34596277

H -0.61179670 -1.89400939 -1.86168376

C 0.92045521 0.53970433 -3.31959652

H 1.02422913 2.67654920 -2.97830221

H 2.33528933 1.76681664 -2.22466266

C 1.42818835 -0.81232625 -2.81575977

P 0.37484113 1.75974975 -0.82520073

P 0.58621495 -1.39428765 -1.27926861

H 1.29932844 -1.57551425 -3.59292295

**P: Fe(CO)_2_BN(CH_3_)_2_(PH_2_CH_2_CH_2_CH_2_PH_2_) + CH_3_Cl**

***E*** = -4069.46

**H**  = -3897.04

**G**  = -3939.29

***N*_imag_** = 0

Fe 0.49431900 -0.19425600 0.18668500

H 0.33517800 -1.69150600 2.29620000

B -1.36842800 -0.06757900 0.57851000

Cl -0.62478500 0.86279000 2.00766500

O 3.36588100 -0.04172300 0.70585700

H -0.55420400 -2.57188700 1.03875100

H 1.21387300 -2.61042300 1.06987000

C 2.22172600 -0.10930400 0.49337500

N -2.75301600 -0.23116400 0.47262400

H -2.52489200 -1.20637400 -1.35985400

H -4.30498200 -0.66196700 1.82799300

C -3.74818600 0.19926300 1.43009000

H -3.27765500 0.72028600 2.26432700

H -4.47404400 0.87843500 0.95884800

C -3.31651300 -0.92243600 -0.66720000

H -3.84914300 -1.83345200 -0.35582900

H -4.03295800 -0.28037000 -1.20142600

C 0.34039800 -1.96798800 1.23515500

H 2.43603200 1.69507800 -2.25246200

P 0.37409200 1.54468300 -1.02557900

H 1.48642400 -1.65405700 -3.71451200

C 1.58601500 -0.87288700 -2.95008800

P 0.64523400 -1.42219600 -1.46277300

C 1.38431600 1.65397800 -2.56324000

H -0.86752900 1.96687100 -1.58625400

H 1.71897200 0.66548500 -4.43746300

H 0.09954700 0.44175200 -3.80854400

H 2.64321900 -0.84199500 -2.65597700

H 1.26238100 -2.69528600 -1.29692700

H 0.69369500 2.82163100 -0.47036800

H -0.51522500 -1.91884000 -2.12774100

C 1.15780300 0.47897200 -3.51134100

H 1.15311500 2.59977600 -3.06723200

**RC: Fe(CO)_2_BN(CH_3_)_2_(PH_2_CH_2_CH_2_CH_2_PH_2_) + C_2_H_6_**

***E*** = -4441.35

**H**  = -4242.03

**G**  = -4284.34

***N*_imag_** = 0

H 3.52635337 -0.44295750 -0.37820119

H 2.70833481 1.82523588 0.90137217

H 1.58773750 1.78239665 -0.45981351

H 3.32743922 1.77608008 -0.74929237

H 1.76526136 -0.46204458 -0.72066430

C 2.54167877 1.43473647 -0.09167163

C 2.54385299 -0.10455512 -0.04501698

H 2.34364899 -0.40602678 0.98642914

H 2.06314398 -1.26839362 -6.57140596

P 0.74361851 1.87935226 -4.99264725

P 0.65771754 -1.27919382 -4.60893970

C -2.33856390 2.01803904 -2.22555675

H -0.59172339 2.35009120 -5.16635385

H 0.53296527 -0.10679853 -8.20779081

H -0.67343850 0.08014353 -6.95387586

H -1.90940117 -1.33026465 -2.14665837

H 1.14948794 -2.60976838 -4.37898073

H 1.30650239 3.19845159 -5.07666713

H -0.70134615 -1.70836248 -4.67066559

C 0.41240628 0.02787490 -7.12305957

H 0.67263127 2.15633067 -7.41054524

H 2.13921627 1.32048218 -6.88127544

C 0.97532443 -1.21706582 -6.43690395

H -2.55828174 2.17855031 -1.16018378

H -1.67339745 2.81510103 -2.56386579

N -1.70774286 0.72991706 -2.43331541

C 1.05082393 1.36592251 -6.74885073

H 0.54625759 -2.11883429 -6.89310304

Fe 1.24944742 0.42702048 -3.65417977

H -3.43194556 -0.48244780 -2.51666276

B -0.43292863 0.60246449 -2.96435468

H -2.69500531 -0.33139823 -0.90320813

O 4.05558564 0.26905585 -4.51073614

H -3.28427264 2.08633578 -2.78271986

C -2.47551786 -0.41176653 -1.97779436

C 2.93525755 0.33140362 -4.17653626

**TS: Fe(CO)_2_BN(CH_3_)_2_(PH_2_CH_2_CH_2_CH_2_PH_2_) + C_2_H_6_**

***E*** = -4395.96

**H**  = -4202.20

**G**  = -4244.30

***N*_imag_** = 1, -671.16 cm^–1^

Fe 0.50723600 -0.03690800 0.12780000

B -1.31696300 -0.03026400 0.16210300

H 0.91142000 -0.88085100 -4.01647100

O 3.42435700 -0.19522100 -0.06905400

P 0.20613300 -0.98896400 -1.68568700

P 0.40298800 1.87726700 -0.66923200

C 2.25771900 -0.13680700 0.01456400

N -2.70530800 -0.03786800 0.17030500

H -2.81951000 -2.04281400 -0.41421100

H -4.11348500 0.88897200 1.43755600

C -3.48073200 1.11966300 0.56814000

H -2.81261700 1.94053700 0.83903600

H -4.13480600 1.45827800 -0.24898300

C -3.48403500 -1.21319000 -0.16213700

H -4.11170200 -1.52062400 0.68682500

H -4.14409000 -1.02230500 -1.02099400

C 0.47544400 -1.56449500 1.77271700

C 0.72052400 0.22314700 2.35239300

H 1.22259700 -1.85502100 2.51558600

H 0.67355500 -2.19079000 0.89879500

H -0.51701600 -1.79994600 2.15522600

H 1.76006900 0.20743100 2.67636100

H 0.07114900 -0.00169100 3.20194900

H 0.46635600 1.23896500 2.04286400

C 1.32361000 2.14092100 -2.25254500

H -0.81461100 2.49029800 -1.08967600

H 1.41593600 1.47195200 -4.28540800

H -0.17726200 1.32720700 -3.57531200

H 2.22425800 -0.46952600 -2.90350700

H 0.47911000 -2.38518400 -1.90862800

H 0.85214400 3.05478700 0.02791200

H -1.05383400 -1.04254000 -2.35332000

C 0.89234000 1.18141400 -3.36367000

H 1.17907100 3.17771300 -2.58098600

H 2.38985700 2.01103900 -2.02969100

C 1.16115200 -0.30414100 -3.11689000

**P: Fe(CO)_2_BN(CH_3_)_2_(PH_2_CH_2_CH_2_CH_2_PH_2_) + C_2_H_6_**

***E*** = -4456.08

**H**  = -4232.63

**G**  = -4272.90

***N*_imag_** = 0

Fe 0.49431900 -0.19425600 0.18668500

H 0.33517800 -1.69150600 2.29620000

B -1.38956084 -0.09964075 0.47207069

C 0.15021521 0.86201906 1.92858958

O 3.36588100 -0.04172300 0.70585700

H -0.55420400 -2.57188700 1.03875100

H 1.21387300 -2.61042300 1.06987000

C 2.22172600 -0.10930400 0.49337500

N -2.77580822 -0.24201544 0.35760036

H -2.55094877 -1.22660733 -1.47025844

H -4.34310477 -0.64422093 1.70408730

C -3.77037977 0.20694723 1.30714331

H -3.29724441 0.72329847 2.14281076

H -4.48248273 0.89579052 0.82900341

C -3.34255401 -0.92814174 -0.78372104

H -3.89132724 -1.82974676 -0.47299111

H -4.04541226 -0.27675091 -1.32466455

C 0.34039800 -1.96798800 1.23515500

H 2.43603200 1.69507800 -2.25246200

P 0.37409200 1.54468300 -1.02557900

H 1.48642400 -1.65405700 -3.71451200

C 1.58601500 -0.87288700 -2.95008800

P 0.64523400 -1.42219600 -1.46277300

C 1.38431600 1.65397800 -2.56324000

H -0.86752900 1.96687100 -1.58625400

H 1.71897200 0.66548500 -4.43746300

H 0.09954700 0.44175200 -3.80854400

H 2.64321900 -0.84199500 -2.65597700

H 1.26238100 -2.69528600 -1.29692700

H 0.69369500 2.82163100 -0.47036800

H -0.51522500 -1.91884000 -2.12774100

C 1.15780300 0.47897200 -3.51134100

H 1.15311500 2.59977600 -3.06723200

H 0.95659877 0.68191478 2.62408078

H -0.78236557 0.53903810 2.36719693

H 0.09653241 1.91727132 1.70507039
